# Imaging and Hemodynamic Mechanisms of Cardiac Remodeling with SGLT2 Inhibitors Across Heart Failure Phenotypes: A Systematic Review

**DOI:** 10.3390/biomedicines14091961

**Published:** 2026-08-31

**Authors:** Diana Evelyne Buzzi, Teodora Mateoc-Sîrb, Samuel Ardelean, Andrei-Catalin Zavragiu, Elena-Larisa Zimbru, Călin Muntean, Minodora Andor

**Affiliations:** 1 Doctoral School of Medicine, “Victor Babes” University of Medicine and Pharmacy, 2nd Eftimie Murgu Square, 340001 Timisoara, Romania; diana.mailat@umft.ro (D.E.B.); teodora.mateoc@umft.ro (T.M.-S.); samuel.ardelean@umft.ro (S.A.); andrei.zavragiu@umft.ro (A.-C.Z.);; 2 Department of Cardiology, Institute of Cardiovascular Diseases Timisoara, Str. Gheorghe Adam No. 13A, 300310 Timisoara, Romania; 3 Multidisciplinary Heart Research Center, “Victor Babes” University of Medicine and Pharmacy, 300041 Timisoara, Romania; andor.minodora@umft.ro; 4 First Department of Internal Medicine, Medical Semiology II, “Victor Babes” University of Medicine and Pharmacy, 300041 Timisoara, Romania; 5 Department of Functional Sciences—Medical Informatics and Biostatistics, “Victor Babes” University of Medicine and Pharmacy, 300041 Timisoara, Romania

**Keywords:** SGLT2 inhibitors, cardiac remodeling, heart failure, echocardiography, cardiac magnetic resonance, hemodynamics, HFrEF, HFpEF

## Abstract

**Background:** Sodium-glucose cotransporter 2 (SGLT2) inhibitors, originally developed as glucose-lowering agents for type 2 diabetes mellitus, have been shown in large, randomized trials to improve clinical outcomes in heart failure (HF) across the ejection fraction (EF) spectrum. The imaging-derived and hemodynamic changes that accompany these benefits remain incompletely characterized, and it is not known whether the patterns of cardiac remodeling observed differ between HF with reduced ejection fraction (HFrEF, left ventricular ejection fraction [LVEF] ≤ 40%) and HF with preserved ejection fraction (HFpEF, LVEF ≥ 50%). This systematic review synthesized randomized evidence on the effects of SGLT2 inhibitors on cardiac remodeling parameters and described the patterns observed in each phenotype. **Methods:** PubMed/MEDLINE, Scopus and Embase were searched for randomized controlled trials (RCTs) published between February 2016 and March 2026. Eligible studies enrolled adults with chronic HF receiving an SGLT2 inhibitor, included a non-SGLT2-inhibitor comparator and assessed cardiac structure, function or loading by echocardiography, cardiac magnetic resonance (CMR) or cardiac catheterization over at least three months. Risk of bias was assessed with Cochrane RoB 2, separately for each outcome domain and targeting the effect of assignment to intervention, and certainty of evidence with GRADE, separately by phenotype and imaging modality. A structured narrative synthesis following Synthesis Without Meta-analysis (SWiM) guidance that prioritized the direction, magnitude and precision of randomized between-group treatment effects was performed. **Results:** Fourteen publications from thirteen unique RCTs (1232 participants) were included: four trials enrolled HFrEF (LVEF ≤ 40%), five enrolled mixed reduced and mildly reduced EF cohorts (LVEF < 50% or 35–49%), four enrolled HFpEF (LVEF ≥ 50%) and one enrolled a mixed ejection-fraction population. No trial enrolled an exclusively HFmrEF population. In reduced EF, the direction of effect on LV volumes favored SGLT2 inhibition in four of eight trials reporting volumes, but the estimates were not compatible in magnitude, and the one adequately powered trial at low risk of bias (EFFORT) was neutral; the largest volumetric estimates came from EMPA-TROPISM, which published no adjusted between-group estimate. The only prespecified, adjusted and confidence-interval-bearing volumetric estimates were those of SUGAR-DM-HF (left ventricular end-systolic volume index −6.0 mL/m^2^, 95% CI −10.8 to −1.2; end-diastolic volume index −8.2 mL/m^2^, 95% CI −13.7 to −2.6). The global longitudinal strain improved in the trial with blinded core-laboratory reading (EFFORT −1.44%, 95% CI −2.42 to −0.46), but effect sizes in the open-label trials without stated reader blinding were two to three times larger, and the CMR-derived strain was unchanged. In HFpEF, volumes, EF, mass and strain were unchanged throughout. The most secure finding of the review was invasive: in CAMEO-DAPA, dapagliflozin reduced pulmonary capillary wedge pressure at rest by 3.5 mmHg (95% CI −6.6 to −0.4) and at peak exercise by 5.7 mmHg (95% CI −10.8 to −0.7). NT-proBNP results were directionally inconsistent, and the trials in which NT-proBNP was a prespecified primary endpoint (Empire HF, CANDLE) were neutral. Certainty of evidence was very low for 18 of 20 evidence profiles and low for the remaining two. **Conclusions:** Randomized imaging and hemodynamic studies suggest phenotype-related differences in the cardiac remodeling associated with SGLT2 inhibitor therapy. In studies enrolling patients with reduced EF, treatment was associated predominantly with reverse remodeling of left ventricular volumes and modest improvements in myocardial function, whereas in preserved EF, the observed changes were mainly confined to filling pressures and diastolic function, with no consistent evidence of left ventricular volumetric remodeling. Given the limited number of studies, substantial heterogeneity, and the overall low certainty of evidence, these findings should be considered hypothesis-generating and warrant confirmation in adequately powered phenotype-specific mechanistic trials.

## 1. Introduction

Heart failure (HF) remains a leading cause of morbidity and mortality worldwide, affecting over 64 million individuals globally [1]. The growing coexistence of HF with type 2 diabetes mellitus (T2DM) and other metabolic disorders has highlighted the complex interplay between metabolic dysfunction and adverse cardiovascular remodeling, underscoring the need for therapies that target both metabolic and cardiac pathways. The pathophysiology of HF involves progressive cardiac remodeling—alterations in ventricular geometry, volume, mass, and function that drive disease progression [2]. Reverse remodeling, defined as the normalization of these structural and functional parameters, has emerged as a surrogate marker of therapeutic efficacy and is associated with improved clinical outcomes [3].

Sodium-glucose cotransporter 2 (SGLT2) inhibitors, initially developed as glucose-lowering agents for T2DM have fundamentally transformed HF treatment. Landmark trials including DAPA-HF [4], EMPEROR-Reduced [5], EMPEROR-Preserved [6], and DELIVER [7] demonstrated that SGLT2 inhibitors reduce cardiovascular death and HF hospitalizations across the EF spectrum, regardless of diabetes status, leading to guideline recommendations as foundational HF therapy [8].

Despite unequivocal clinical benefits, the mechanisms by which SGLT2 inhibitors improve HF outcomes remain incompletely understood. Proposed mechanisms include natriuresis and plasma volume contraction, improved myocardial energetics through ketone utilization, inhibition of the sodium-hydrogen exchanger (NHE1/NHE3), reduction in epicardial adipose tissue and direct effects on cardiomyocyte calcium handling [9,10]. Recent reviews of preclinical and translational evidence have further highlighted the attenuation of oxidative stress, inflammation and profibrotic signaling as potential contributors to the cardioprotective effects of SGLT2 inhibitors. Experimental studies suggest that these agents reduce reactive oxygen species generation, improve mitochondrial function and redox homeostasis [11], and attenuate inflammatory and fibrotic remodeling pathways involved in cardiometabolic disease [12]. Collectively, these mechanisms provide a biologically plausible framework through which SGLT2 inhibitors may influence cardiac structure and function, although their relative contribution to cardiac remodeling in patients with heart failure remains to be fully elucidated.

Cardiac imaging—echocardiography, speckle-tracking echocardiography (STE) and cardiac magnetic resonance (CMR)—provides a non-invasive window into the structural and functional changes underlying reverse remodeling. Key parameters include left ventricular end-diastolic volume (LVEDV), end-systolic volume (LVESV), ejection fraction (LVEF), mass index (LVMi), global longitudinal strain (GLS), diastolic function indices (E to e′ ratio [E/e′]), and left atrial volume index (LAVI) [13]. Invasive hemodynamic assessments, particularly pulmonary capillary wedge pressure (PCWP), offer complementary insights into loading conditions and ventricular compliance [14]. N-terminal pro-B-type natriuretic peptide (NT-proBNP) serves as an established biomarker of myocardial wall stress [15].

Importantly, the mechanisms of cardiac remodeling may differ fundamentally between HFrEF and HFpEF. In HFrEF, ventricular dilatation with eccentric hypertrophy predominates, and reverse remodeling involves reduced chamber volumes, improved systolic function, and regression of hypertrophy [3]. In HFpEF, concentric remodeling with diastolic dysfunction, left atrial enlargement, and elevated filling pressures are central features [16]. Whether SGLT2 inhibitors engage distinct remodeling pathways in these two phenotypes has not been systematically examined.

Previous meta-analyses have pooled remodeling data [17,18] but largely combined HF phenotypes without explicitly comparing imaging and hemodynamic mechanisms across the EF spectrum. The aim of this systematic review was to evaluate the imaging, biomarker and hemodynamic changes in cardiac remodeling reported in randomized trials of SGLT2 inhibitors across heart failure phenotypes, and to describe the patterns observed in reduced ejection fraction (HFrEF, LVEF ≤ 40%; HFmrEF, LVEF 41–49%) compared with preserved ejection fraction (HFpEF, LVEF ≥ 50%).

## 2. Materials and Methods

This systematic review was conducted following PRISMA 2020 [19] and Synthesis Without Meta-analysis (SWiM) [20] reporting guidelines. The PRISMA S-Checklist is given in Supplementary Table S5. The protocol was prospectively registered in PROSPERO registration number: CRD420261372805.

### 2.1. Eligibility Criteria

Eligibility criteria were defined according to a PICO framework. Studies were included if they met the following criteria: randomized controlled trial design, with all analyzed participants randomly allocated; enrollment of adult patients (≥18 years) with chronic heart failure across the spectrum of LVEF; evaluation of an SGLT2 inhibitor in addition to guideline-directed medical therapy; inclusion of a comparator group receiving placebo, standard care or an active control that did not contain an SGLT2 inhibitor; and assessment of cardiac remodeling using echocardiography, speckle-tracking echocardiography (STE), CMR or cardiac catheterization with a minimum follow-up of three months. Heart failure phenotypes were defined a priori according to contemporary ESC/AHA criteria as HFrEF (LVEF ≤ 40%), HFmrEF (LVEF 41–49%) and HFpEF (LVEF ≥ 50%). Studies were required to report at least one primary outcome; secondary outcomes were extracted when available. Both blinded and open-label RCTs were eligible, and the adequacy of outcome ascertainment was assessed within the risk-of-bias appraisal rather than used as an eligibility filter. Primary outcomes were changes in core left ventricular reverse remodeling parameters: left ventricular end-diastolic volume (LVEDV or LVEDVi), left ventricular end-systolic volume (LVESV or LVESVi) and LVEF. Secondary outcomes reflected complementary domains of cardiac remodeling: structural (left ventricular mass index [LVMi] and left atrial volume index [LAVI]); functional (global longitudinal strain [GLS] and E/e′ ratio); and hemodynamic and neurohormonal (pulmonary capillary wedge pressure [PCWP], where available, and N-terminal pro-B-type natriuretic peptide [NT-proBNP]). Outcomes were analyzed with consideration of heart failure phenotype to describe patterns of remodeling across the EF spectrum.

Studies were excluded if they were (1) not randomized controlled trials; (2) duplicate publications; (3) meta-analysis studies; (4) ongoing or unpublished study; (5) observational, cohort or prospective but not randomized studies; (6) studies not published in English; (7) studies on non-human subjects; (8) mechanistic imaging without remodeling endpoints (e.g., Phosphorus-31 Magnetic Resonance Spectroscopy energetics or T1/T2 mapping without volumetric data); (9) cardiac remodeling studies on post-acute myocardial infarction patients or acute heart failure patients.

### 2.2. Search Strategy

A systematic search of PubMed/MEDLINE, Scopus and Embase was conducted for the period February 2016 to March 2026. The search strategy is based on a three-concept structure—SGLT2 inhibitors, heart failure, and remodeling or imaging or haemodynamic outcomes—while adding controlled vocabulary (MeSH and Emtree), truncated free-text variants, and the Cochrane Highly Sensitive Search Strategy for identifying randomized trials in its sensitivity-maximizing form. An English-language restriction was applied. The complete line-by-line strategy for each database, with platform, search date, controlled vocabulary, free-text terms, Boolean operators, field and language restrictions, the RCT filter and the yield of every line, is provided as a Supplementary Materials (Table S6a).

The strategy was adapted for Scopus and Embase while preserving the same three concepts, using Emtree controlled vocabulary and the Cochrane Embase filter for Embase, and title-abstract-keyword field searching for Scopus, which has no thesaurus. Reference lists of included articles and of relevant systematic reviews were screened for additional studies; as reported in Section 3.1 and Supplementary Table S6b, this yielded no publication that had not already been retrieved by the database search. Only English-language randomized controlled trials were eligible.

### 2.3. Study Selection

Two independent reviewers (D.E.B. and T.M.-S.) independently screened the title and abstract of all records identified through the systematic database search. Records were classified as “Include”, “Exclude” or “Maybe” against the predefined eligibility criteria. Records classified as “Maybe” by either reviewer were retained for full-text assessment. Because the review’s outcomes are frequently reported as secondary or substudy endpoints, any record describing a randomized trial of an SGLT2 inhibitor in chronic heart failure that mentioned cardiac imaging, invasive haemodynamic assessment or a cardiac functional assessment was retained for full-text assessment even when the abstract reported only a non-imaging primary endpoint.

Full-text articles of all potentially eligible studies were retrieved and assessed independently by both reviewers against all inclusion and exclusion criteria. For each excluded study, the primary reason for exclusion was recorded and categorized according to a pre-specified taxonomy: (A) outcome trial without imaging endpoints; (B) symptom or quality-of-life trial without imaging; (C) mechanistic study without remodeling data; (D) population without heart failure diagnosis; (E) sub-analysis with non-remodeling focus; (F) protocol or design paper only. When a study met multiple exclusion criteria, the most proximal reason was recorded.

Disagreements between reviewers were resolved through a structured consensus process. At both screening stages, discordant decisions were first discussed by the two reviewers with reference to the eligibility criteria. When consensus could not be reached through discussion, a third reviewer (M.A.) was consulted. The third reviewer assessed each discrepant case independently, blinded to the identity of the dissenting reviewer, and the majority decision was adopted. All discrepancies, their categories, and resolution methods were documented in a Discrepancy Log (Supplementary Table S4).

Inter-rater reliability was quantified using Cohen’s kappa (κ) coefficient, calculated from 2 × 2 agreement tables at each screening stage [21]. For this purpose, decisions were dichotomized as “retain for full-text assessment” (Include or Maybe) versus “exclude”, since that is the operational consequence of the title/abstract decision. Agreement was almost perfect at both stages: κ = 0.994 (95% BCa CIs 0.983–0.998) at the title/abstract stage and κ = 0.937 (95% BCa CIs 0.758–1.000) at the full-text stage. The number of discordant decisions in each agreement table is recorded in the Discrepancy Log (Supplementary Table S4).

### 2.4. Data Extraction

Two investigators independently extracted the outcome data (LVEDV/i, LVESV/i, LVEF, GLS, E/e′, LAVI, PCWP, LVMi, NT-proBNP). Because indexed and non-indexed measures of the same structure are not interchangeable, absolute and body-surface-area-indexed volumes, masses and atrial volumes were recorded and synthesized separately. Additional data extraction was performed using a standardized form: first author, study acronym, year, sample size, demographics, comorbidities, HF phenotype, SGLT2 inhibitor type/dose, imaging modality, treatment duration. When multiple publications originated from the same trial, data were extracted separately by outcome domain while accounting for overlapping populations to avoid double counting. In studies that included mixed HF population and reported separated outcomes for HFrEF (EF ≤ 40%), HF with reduced and mildly reduced EF (EF < 50%) and HFpEF (EF ≥ 50%), data were also extracted separately for each outcome.

### 2.5. Risk of Bias and GRADE Assessment

**Figure 1:**
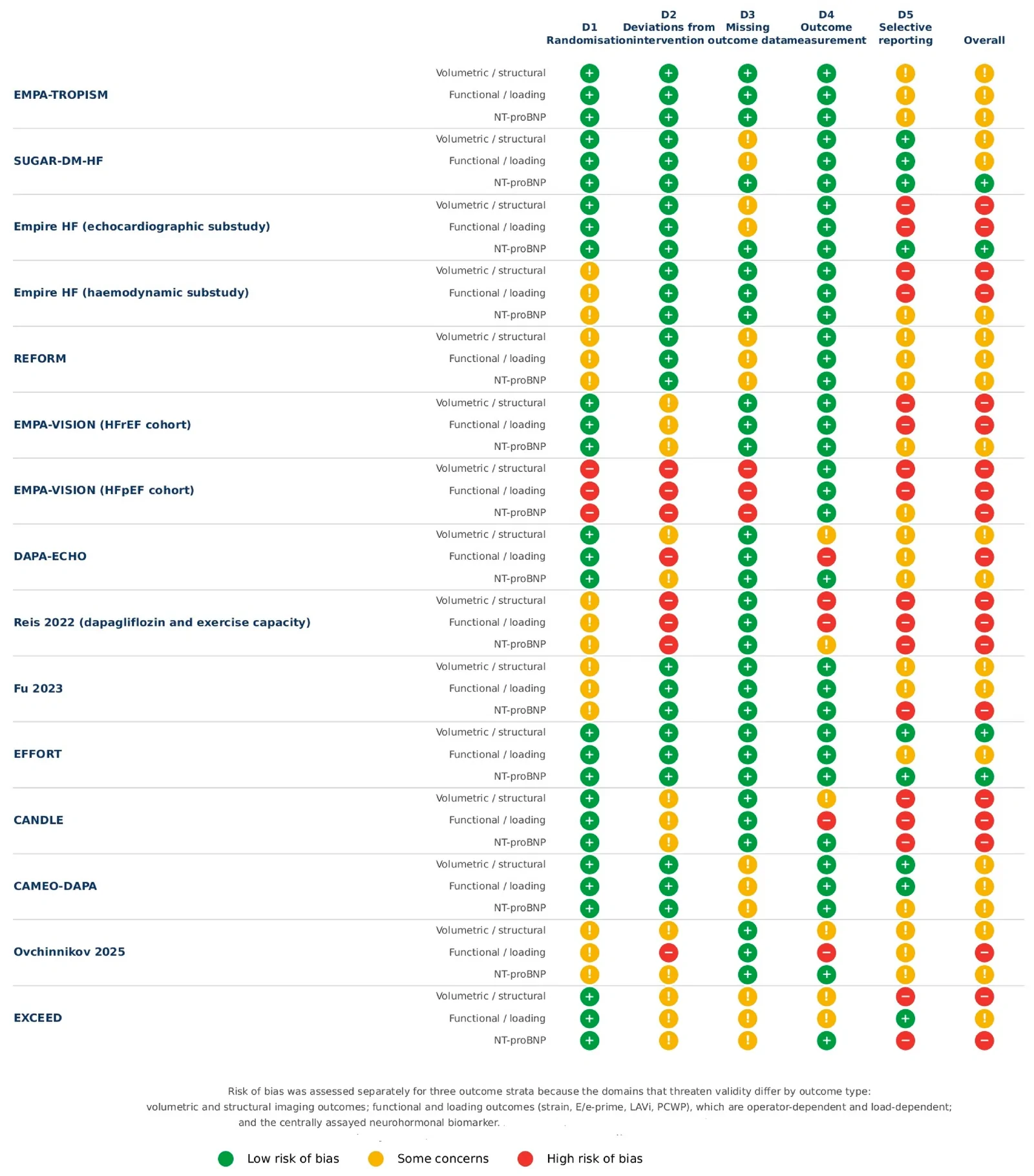
Cochrane RoB 2 assessment, by study and outcome domain. The figure includes EMPA-TROPISM [24], SUGAR-DM-HF [25], Empire HF echocardiographic substudy [26], Empire HF haemodynamic substudy [27], REFORM [28], EMPA-VISION [29], DAPA-ECHO [30], Reis et al. [31], Fu et al. [32], EFFORT [33], CANDLE [34], CAMEO-DAPA [35], Ovchinnikov et al. [36], and EXCEED [37]. The target of measurement is the effect of assignment to intervention (intention-to-treat effect) for every study and every outcome. Complete signaling-question responses and written justifications are given in Supplementary Table S8.

Risk of bias was assessed using the Cochrane RoB 2 tool [22] across its five domains, with the effect of assignment to intervention (intention-to-treat effect) defined as the effect of interest. When analyses were restricted to participants with complete paired imaging, this was considered within the missing outcome data domain and, when relevant, the deviations from intended interventions domain. Risk of bias was evaluated separately for three outcome strata: volumetric and structural imaging outcomes; functional and loading outcomes (strain, E/e′, LAVI, and PCWP); and NT-proBNP. Overall judgements are presented in Figure 1, while signaling-question responses and study-specific justifications are provided in Supplementary Table S7. Certainty of evidence was assessed using the GRADE framework [23] across risk of bias, inconsistency, indirectness, imprecision, and publication bias. Evidence profiles were assessed separately by heart failure phenotype and, where applicable, by imaging modality (CMR and echocardiography). GRADE assessments are summarized in Table 1, with complete evidence profiles and domain-specific justifications provided in Supplementary Tables S3 and S3b.

### 2.6. Data Synthesis Strategy

**Figure 2:**
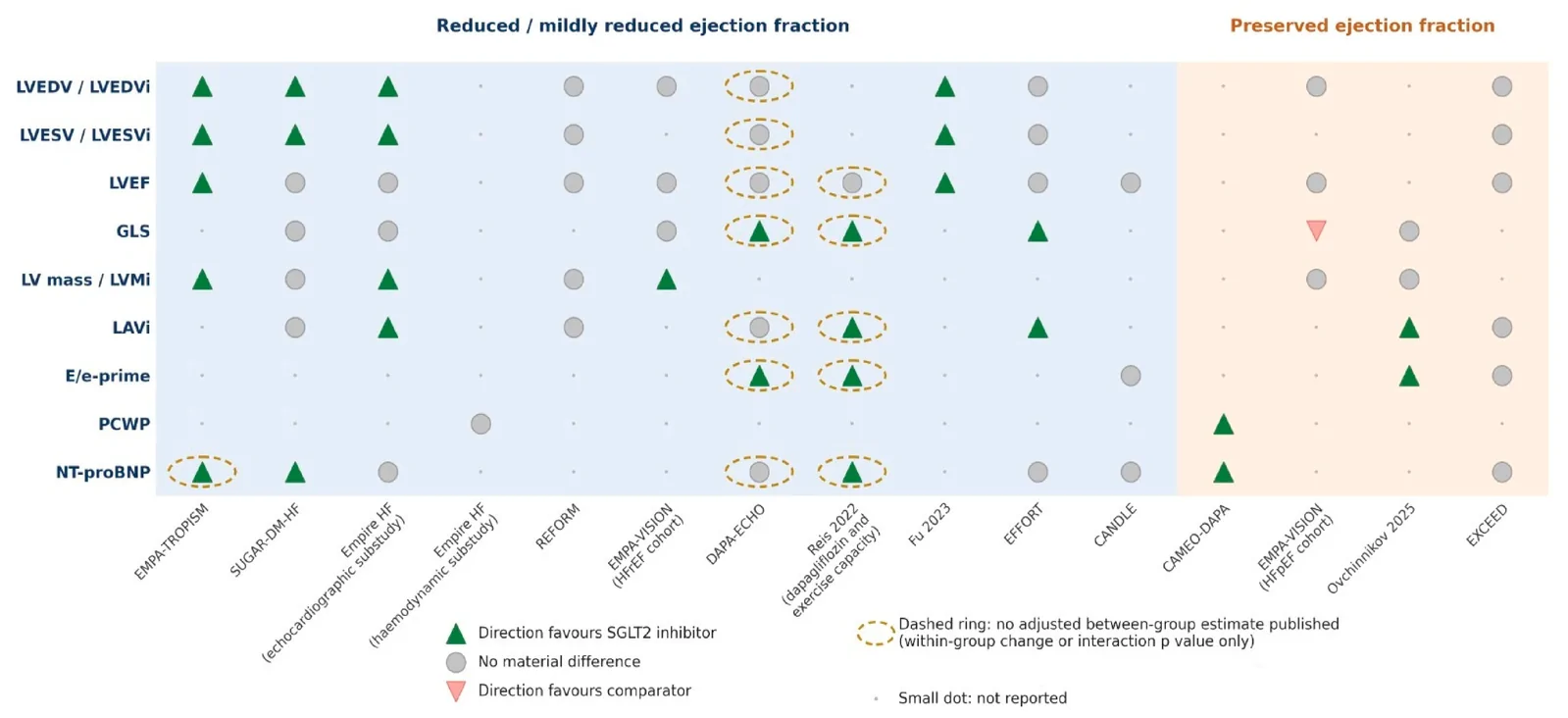
Effect-direction plot of randomized between-group treatment effects by outcome and study. Glyph shape indicates the direction of effect interpreted clinically; a dashed ring indicates that the source publication reported no adjusted between-group estimate. Where a trial reported an outcome both as a prespecified primary endpoint and in post hoc analyses, the prespecified primary result is plotted.

Formal meta-analysis was not performed; the reasons are set out separately for each principal outcome in Section 4.6. from Discussion. A narrative synthesis adhering to the SWiM guideline [20] based primarily on the direction and magnitude of randomized between-group treatment effects and their 95% confidence intervals was performed. For each study and outcome, Supplementary Table S2 reports the effect direction, point estimate, confidence interval, *p* value, imaging modality, follow-up duration, heart failure phenotype, risk-of-bias judgment, and type of reported contrast. When an adjusted between-group estimate was unavailable, this was explicitly indicated; where arm-level change data permitted calculation of a between-group difference, the derived estimate was identified accordingly. Effect direction was interpreted according to the clinical meaning of each outcome, accounting for differences in sign conventions, particularly for myocardial strain. Interpretation considered study size, risk of bias, outcome prespecification, and precision of the effect estimate. Study-level findings are summarized visually in the effect-direction plot (Figure 2).

## 3. Results

### 3.1. Study Selection (PRISMA Flow)

**Figure 3:**
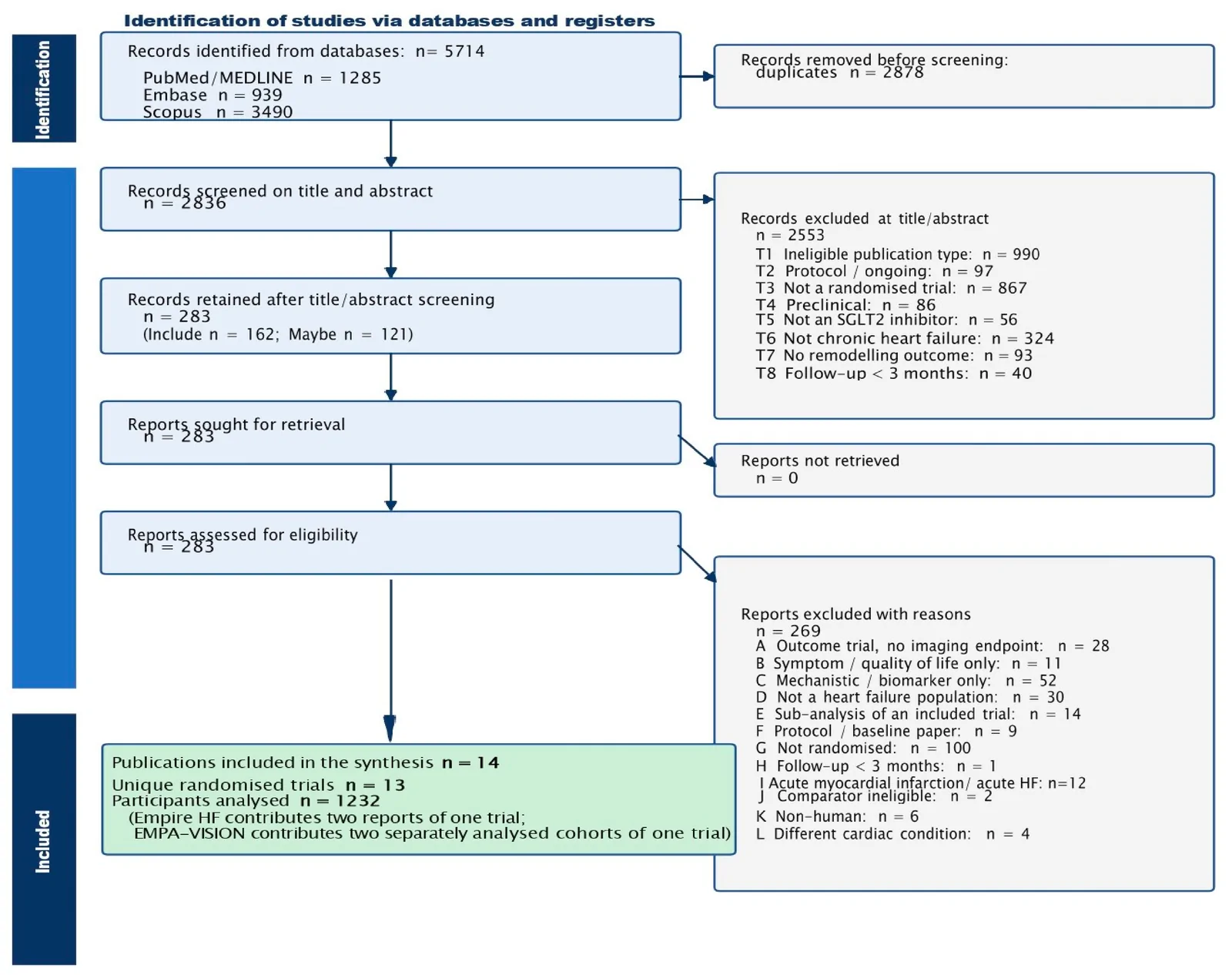
PRISMA 2020 flow diagram. The search was last executed on 31st July 2026 for publication dates 1st February 2016 to 31st March 2026, restricted to English language, with the Cochrane sensitivity-maximizing randomized controlled trial filter applied. The full line-by-line strategy is given in Supplementary Table S6a, and the retrieval of each included study by each database is given in Supplementary Table S6c.

A total of 5714 English-language records published between February 2016 and March 2026 were retrieved from PubMed, Embase and Scopus database. Of these, 2878 duplicates were identified and removed using the Rayyan systematic review platform, 2553 were excluded at the title/abstract stage and 283 were retained and retrieved in full. All 283 reports were assessed for eligibility, and 261 were excluded with documented reasons: not a randomized controlled trial (*n* = 98); mechanistic or biomarker study without remodeling data (*n* = 52); population without chronic heart failure (*n* = 29); event-driven outcome trial or secondary analysis without an imaging endpoint (*n* = 25); sub-analysis of an already represented trial (*n* = 14); post-acute myocardial infarction or acute heart failure population (*n* = 12); symptom or quality-of-life report only (*n* = 10); protocol or baseline-characteristics paper (*n* = 9); non-human study (*n* = 6); a distinct cardiac condition outside the review population (*n* = 3); ineligible comparator in which both randomized arms received an SGLT2 inhibitor (*n* = 2); and follow-up shorter than three months (*n* = 1). Fourteen publications from thirteen unique randomized trials (1232 participants analyzed) are included in the synthesis (Figure 3). Reference-list screening yielded no publication that had not already been retrieved by the database search. Because no randomized trial enrolled an exclusively HFmrEF population, a separate phenotype-specific synthesis for HFmrEF was not feasible; the single trial spanning the HFmrEF range (EFFORT, LVEF 35% to <50%) is interpreted within the broader reduced EF evidence while being identified throughout as a mixed cohort.

### 3.2. Study Characteristics

Table 2 presents the characteristics of the 14 included publications, which report 13 unique randomized trials. Sample sizes ranged from 38 to 233 participants analyzed. Five SGLT2 inhibitors were studied: empagliflozin (six publications from five trials), dapagliflozin (four trials), ertugliflozin, canagliflozin and ipragliflozin (one trial each). Treatment duration ranged from 12 weeks to 12 months. Eight studies were double-blind placebo-controlled trials and five were open-label trials. Based on the classification by ESC/AHA criteria, four trials enrolled HFrEF (LVEF ≤ 40%: SUGAR-DM-HF, Empire HF, the EMPA-VISION HFrEF cohort and Fu et al.), five enrolled mixed reduced and mildly reduced EF cohorts (EMPA-TROPISM and Reis et al. at LVEF < 50%, DAPA-ECHO at LVEF < 50% with 38% HFmrEF, EFFORT at LVEF 35% to <50%, and REFORM, which states no numerical EF threshold), four enrolled HFpEF (LVEF ≥ 50%: the EMPA-VISION HFpEF cohort, CAMEO-DAPA, Ovchinnikov et al. and EXCEED) and one enrolled a mixed-ejection-fraction population (CANDLE, mean LVEF 57.6 ± 14.6%, 71% with LVEF ≥ 50%). EFFORT is therefore not a pure HFmrEF cohort and is not described as one, and CANDLE is not assigned exclusively to either phenotype. A study-publication map distinguishing unique trials from publications, and identifying overlapping populations and phenotype-specific cohorts is given in Supplementary Table S10.

### 3.3. Risk of Bias Assessment

Of the 45 study-by-outcome-stratum judgements, four were low risk, 21 raised some concerns and 20 were high risk. Only EFFORT achieved low risk of bias for its volumetric and biomarker outcomes, and only SUGAR-DM-HF and the Empire HF parent trial achieved low risk for NT-proBNP [25,33]. The recurring reasons for high-risk judgements were domain 5, selection of the reported result, and domain 4, measurement of the outcome. The echocardiographic outcomes of the Empire HF substudy were, by the authors’ own statement, not prespecified in the parent trial and are described there as an exploratory post hoc analysis, and the statistical significance of its end-systolic volume index and left atrial volume index depends on which prespecified adjustment is applied [26]. Every CMR outcome of EMPA-VISION is labeled exploratory in the source tables, analyzed on the per-protocol set, and drawn from a large panel of correlated measures without control for multiplicity; in its HFpEF cohort only 24 of 36 randomized participants were analyzed, with marked baseline imbalance, giving high risk of bias in three domains [29]. In the Empire HF hemodynamic substudy, the prespecified primary endpoint was neutral and the reported reduction in PCWP derives from a pooled across-exercise-load analysis that the trialists describe as post hoc. In CANDLE and EXCEED, the prespecified primary endpoints were not met and the results carried into this review from EXCEED are post hoc subgroup analyses [34,37]. Neither DAPA-ECHO, Reis et al. nor Ovchinnikov et al. reports that echocardiographic or strain readers were blinded to allocation, and EXCEED performed echocardiography at each participating site rather than in a core laboratory, which its authors identify as a limitation [30,31,36,37]. Because strain, E/e′ and left atrial volume are operator-dependent and load-dependent, and because an unblinded treating team can adjust diuretic therapy with knowledge of allocation, domain 4 was rated high risk for the functional and loading outcomes of these trials. In Reis et al., the control arm deteriorated across nearly every measure over six months, a pattern not expected in stable ambulatory heart failure on optimal therapy, so its large apparent effects are attributable in substantial part to control-arm deterioration [30,31,34,36,37,38]. No study was excluded on the grounds of risk of bias, but the judgements are reflected in the certainty ratings and in the weight given to each study in the narrative synthesis.

### 3.4. GRADE Certainty of Evidence

Certainty of evidence was assessed using the GRADE framework separately for each outcome, with distinct evidence profiles prepared according to heart failure phenotype and, where applicable, imaging modality. Across the 20 evidence profiles, certainty was rated as very low for 18 outcomes and low for two; no outcome was rated as high certainty. The lower certainty ratings primarily reflected study limitations related to risk of bias, inconsistency, indirectness arising from heterogeneous patient populations and imaging modalities, imprecision due to small sample sizes, and the limited number of contributing studies. Publication bias was considered for all outcomes but could not be formally assessed because no comparison included more than eight studies. Summary GRADE assessments are presented in Table S3a, while the complete rationale for each domain judgment is provided in Supplementary Table S3b.

### 3.5. Narrative Synthesis by HF Phenotype

#### 3.5.1. Reduced and Mildly Reduced EF (LVEF ≤ 40% and Mixed Cohorts with LVEF < 50%)

Nine publications from eight trials enrolled populations with reduced or mildly reduced EF (787 participants analyzed). Four of these trials restricted entry to LVEF ≤ 40% and four enrolled mixed cohorts spanning the HFrEF and HFmrEF ranges.

Volumetric remodeling (LVEDV, LVESV). The direction of effect on left ventricular volumes favored SGLT2 inhibition in four of the eight trials reporting volumes, but the estimates are not compatible in magnitude and the pattern tracks study quality. The only prespecified, adjusted, confidence-interval-bearing estimates come from SUGAR-DM-HF [25], in which empagliflozin reduced LVESVi by 6.0 mL/m^2^ (95% CI −10.8 to −1.2, *p* = 0.015, co-primary endpoint) and LVEDVi by 8.2 mL/m^2^ (95% CI −13.7 to −2.6, *p* = 0.004) by CMR. EMPA-TROPISM reported the largest changes, but publishes only arm-level change scores with a between-arm *p* value and no adjusted estimate or confidence interval for any outcome: LVEDV changed by −25.1 ± 26.0 mL with empagliflozin against −1.5 ± 25.4 mL with placebo and LVESV by −26.6 ± 20.5 against −0.5 ± 21.9 mL (both *p* < 0.001), giving derived between-group differences of −23.6 mL and −26.1 mL [24]. The Empire HF echocardiographic substudy reported reductions in LVEDVi of 5.5 mL/m^2^ (95% CI −10.6 to −0.4, *p* = 0.03) and LVESVi of 4.3 mL/m^2^ (95% CI −8.5 to −0.1, *p* = 0.04) at 12 weeks, but these outcomes were not prespecified in the parent trial and the LVESVi result becomes non-significant under an alternative prespecified adjustment (−4.0 mL/m^2^, 95% CI −8.2 to 0.2, *p* = 0.06) [26]. Fu et al. reported reductions in LVEDV of 6.0 mL (95% CI −8.07 to −3.87) and LVESV of 8.1 mL (95% CI −11.07 to −5.14), both *p* < 0.001, with confidence intervals that are strikingly narrow for a trial of 60 participants [32].

Four trials were neutral, and they include the largest and least biased. REFORM, whose primary endpoint this was, found no volumetric change (LVESV adjusted difference +4.82 mL, 95% CI −13.28 to 22.93, *p* = 0.594; LVEDV +7.83 mL, 95% CI −15.05 to 30.70, *p* = 0.495) [28]. EMPA-VISION reported no change in LVEDV in its HFrEF cohort (−7.00 mL, 95% CI −21.98 to 8.85, *p* = 0.39) and did not report end-systolic volume at all [29]. DAPA-ECHO found no change in either volume index, and EFFORT, the only adequately powered multicenter trial at low risk of bias for this outcome, reported no significant between-group difference in left ventricular volume indices [30,33].

LVEF. Seven of the nine estimates of LVEF are null and mutually consistent: SUGAR-DM-HF +0.3 percentage points (95% CI −1.7 to 2.3, *p* = 0.75), REFORM +0.69 (95% CI −3.32 to 4.69, *p* = 0.732), EMPA-VISION HFrEF +1.87 (95% CI −1.60 to 5.40, *p* = 0.29), the Empire HF echocardiographic substudy +1.2 (95% CI −1.2 to 3.6, *p* = 0.32), DAPA-ECHO +1.2 (*p* = 0.38), Reis et al. +1.7 (interaction *p* = 0.512) and EFFORT, which reported no significant difference [25,28,29,30,31,33]. Two trials are discrepant: EMPA-TROPISM, with a derived between-group difference of +6.1 percentage points (arm-level changes +6.0 ± 4.2 against −0.1 ± 3.9, *p* < 0.001) [24], and Fu et al., with +2.5 percentage points (95% CI 1.00 to 4.06, *p* = 0.002, its prespecified primary endpoint) [32]. The EMPA-TROPISM estimate lies outside the confidence intervals of every other trial.

GLS. In EFFORT, the only contributing trial with blinded core-laboratory strain analysis, ertugliflozin improved GLS by 1.44 percentage points (95% CI −2.42 to −0.46, *p* = 0.004) [33]. The two open-label trials that do not state whether strain readers were blinded reported considerably larger effects: DAPA-ECHO recorded a within-group change of −2.5 percentage points in the dapagliflozin arm against −0.3 in the comparator arm, with a significant treatment-by-time effect (*p* < 0.0001) but no adjusted between-group estimate or confidence interval [30], and Reis et al. recorded a derived between-group difference of −3.8 percentage points (interaction *p* < 0.001) in which the treated arm improved from −8.2% to −10.4% while the control arm deteriorated from −8.4% to −6.8% [31]. Strain measured by CMR feature tracking was unchanged in both trials that reported it: SUGAR-DM-HF, where GLS was a co-primary endpoint, found +0.35 percentage points (95% CI −0.25 to 0.95, *p* = 0.25) [25], and EMPA-VISION HFrEF found +1.95 (95% CI −0.60 to 4.50, *p* = 0.12); in both, the positive sign denotes strain becoming less negative [29]. The Empire HF echocardiographic substudy also found no change (+0.4, 95% CI −0.3 to 1.0, *p* = 0.31) [26].

Left ventricular mass (LVM). Two of the four CMR trials reported a reduction and two did not. EMPA-TROPISM reported arm-level mass changes of −17.8 ± 31.9 g against +4.1 ± 13.4 g (*p* < 0.001), a derived between-group difference of −21.9 g in absolute mass [24]. EMPA-VISION HFrEF reported reductions in mass of 9.65 g (95% CI −17.50 to −1.80) and in mass index of 4.46 g/m^2^ (95% CI −8.42 to −0.50), both exploratory outcomes [29]. By contrast SUGAR-DM-HF found no change in mass index (−1.9 g/m^2^, 95% CI −4.7 to 0.8, *p* = 0.17) and REFORM found none (+2.5 g/m^2^, 95% CI −3.95 to 8.95, *p* = 0.44) [25,28]. The Empire HF echocardiographic substudy reported a reduction in mass index of 9.0 g/m^2^ (95% CI −17.2 to −0.8, *p* = 0.03), an outcome that was not prespecified [26].

LAVI. The Empire HF echocardiographic substudy reported −2.5 mL/m^2^ (95% CI −4.8 to −0.1, *p* = 0.04), falling to −2.2 mL/m^2^ (95% CI −4.6 to 0.2, *p* = 0.07) under alternative prespecified adjustment [26]. EFFORT reported −6.0 mL/m^2^, although its published 95% CI (−12.16 to 0.15) crosses zero despite a stated *p* of 0.005 [33]. Reis et al. reported a derived difference of −13.1 mL/m^2^ (interaction *p* < 0.001), generated largely by a rise in the control arm from 41.9 to 48.1 mL/m^2^ [31]. DAPA-ECHO (−1.0 mL/m^2^ within group, *p* = 0.49), SUGAR-DM-HF (−2.4 mL/m^2^, 95% CI −6.5 to 1.8, *p* = 0.26) and REFORM (−2.6 mL/m^2^, 95% CI −9.67 to 4.48, *p* = 0.464) were all neutral [25,28].

E/e′. Only two trials in reduced EF reported this outcome, neither prespecified it, and neither published an adjusted between-group estimate. DAPA-ECHO reported a within-group change of −1.7 (95% CI −3.2 to −0.25, *p* = 0.022) in the dapagliflozin arm against +0.6 (95% CI −0.91 to 2.09, *p* = 0.43) in the comparator arm [30]. Reis et al. reported a derived between-group difference of −2.9 (interaction *p* = 0.026), with the treated arm falling from 13.3 to 11.9 and the control arm rising from 11.8 to 13.3 [31]. Both trials were open-label, single-center and without placebo, and neither states that readers were blinded to allocation; since E/e′ is load-dependent and diuretic therapy could be adjusted with knowledge of allocation, certainty is very low.

NT-proBNP. The direction of effect is inconsistent, and the two trials at low risk of bias for this outcome disagree with each other. SUGAR-DM-HF, the only trial providing a prespecified adjusted estimate, reported a between-group reduction of 28% (95% CI −47% to −2%, *p* = 0.038) on the log scale [25]. EFFORT, also at low risk of bias, found no significant difference [33], and the Empire HF parent trial, in which NT-proBNP was the registered primary endpoint, was likewise neutral [26]. EMPA-TROPISM reported an 11.5% decrease against an 8.5% increase with placebo (*p* = 0.01) without an adjusted estimate [24]. Reis et al. reported a derived difference of −868 pg/mL (interaction *p* = 0.007), driven by a rise of 650 pg/mL in the control arm rather than by the fall of 218 pg/mL in the treated arm [31]. DAPA-ECHO found a numerically greater median reduction with dapagliflozin (−560 against −90 pg/L) that was not statistically significant [30].

Invasive hemodynamics (PCWP). One trial provided invasive data in reduced EF, and its prespecified primary endpoint was neutral. In the Empire HF hemodynamic substudy, the registered primary endpoint, the ratio of PCWP to cardiac index at peak exercise, showed no treatment effect (0.13 mmHg/L/min/m^2^, 95% CI −1.60 to 1.34, *p* = 0.86), and resting PCWP did not differ between groups (−1.47 mmHg, 95% CI −3.86 to 0.82, *p* = 0.23). PCWP considered across the full range of exercise loads was reduced (−2.40 mmHg, 95% CI −3.96 to −0.84, *p* = 0.003), but the trialists describe that pooled analysis as post hoc, and the trial reports significant baseline imbalance between arms in peak-exercise PCWP. Empagliflozin did reduce plasma volume (−164.9 mL, 95% CI −236.14 to −93.65, *p* < 0.001) [27].

#### 3.5.2. Preserved EF (LVEF ≥ 50%)

Four trials enrolled dedicated HFpEF populations with LVEF ≥ 50% (the EMPA-VISION HFpEF cohort, CAMEO-DAPA, Ovchinnikov et al. and EXCEED; 212 participants analyzed) [29,35,36,37]. CANDLE enrolled a mixed-ejection-fraction population; however, its post hoc analysis of participants with LVEF ≥ 50% provides phenotype-specific echocardiographic data for LVEF and E/e′ and is included in the corresponding outcome-specific synthesis [34]. Three of the four dedicated trials enrolled entirely or almost entirely diabetic cohorts, and EXCEED excluded atrial fibrillation and valvular disease, which materially restricts the population to which these findings apply [37].

Volumetric remodeling and systolic function. Volumes, EF, mass and strain were unchanged in every trial, and the estimates are mutually compatible. EXCEED found no change in LVEDV (−4.9 mL, 95% CI −14.9 to 5.1, *p* = 0.33), LVESV (−1.7 mL, 95% CI −6.4 to 3.1, *p* = 0.48) or LVEF (+1.3 percentage points, 95% CI −1.4 to 4.0, *p* = 0.34) [37]. EMPA-VISION HFpEF found no change in LVEDV (−2.74 mL, 95% CI −20.44 to 14.95, *p* = 0.75), LVEF (+1.47, 95% CI −2.89 to 5.83, *p* = 0.49) or mass index (−1.18 g/m^2^, 95% CI −7.06 to 4.71, *p* = 0.68) [29]. Ovchinnikov et al. likewise found no change in LVEDV (−0.2 mL, *p* = 0.93), LVEF (−0.1 percentage points, *p* = 0.58), GLS (−0.5, 95% CI −1.4 to 0.3, *p* = 0.22) or mass index (−2.8 g/m^2^, 95% CI −7.9 to 2.3, *p* = 0.28) and CANDLE HFpEF subgroup showed no change in LVEF (+0.31 percentage points, 95% CI −1.96 to 2.58, *p* = 0.789) [34,36].

Diastolic function and E/e′. Findings for E/e′ in HFpEF were inconsistent across the three contributing datasets. In EXCEED, where E/e′ and e′ were registered co-primary endpoints analyzed with multivariable adjustment, there was no effect (E/e′ −0.04, 95% CI −1.3 to 1.2, *p* = 0.95; e′ −0.3 cm/s, 95% CI −0.9 to 0.3, *p* = 0.33) [37]. Similarly, the post hoc LVEF ≥ 50% subgroup of the mixed-ejection-fraction CANDLE trial showed no significant between-group difference in E/e′ change (−0.60, 95% CI −1.90 to 0.70, *p* = 0.365) [34]. In contrast, Ovchinnikov et al., an open-label single-center trial in which E/e′ was a secondary endpoint and reader blinding is not stated, E/e′ fell by 1.8 (95% CI −2.6 to −1.1, *p* < 0.001) and LAVI by 3.4 mL/m^2^ (95% CI −5.1 to −1.6, *p* < 0.001) at rest, with improvements in e′ velocity, left atrial reservoir strain and, during exercise diastolic stress testing, in diastolic, atrial and chronotropic reserve [36]. EXCEED also found no change in absolute left atrial volume (−7.1 mL, 95% CI −14.4 to 0.2, *p* = 0.06).

LVMi. All three overall estimates in HFpEF were null and mutually compatible: EMPA-VISION HFpEF −1.18 g/m^2^ (95% CI −7.06 to 4.71, *p* = 0.68) [29], Ovchinnikov et al. −2.8 g/m^2^ (95% CI −7.9 to 2.3, *p* = 0.28) and EXCEED non-significant overall [36]. The only positive result is a post hoc subgroup of 38 participants aged 70 years or over within EXCEED (−19.9 g/m^2^, 95% CI −38.0 to −1.75, *p* = 0.033), in a trial whose two co-primary endpoints were both neutral and which enrolled fewer participants than its own power calculation required [37].

Invasive hemodynamics (PCWP). CAMEO-DAPA provides the most secure finding in this review. Its prespecified composite primary endpoint, the change in PCWP at rest and during exercise, was met (likelihood ratio test *p* < 0.001): dapagliflozin reduced resting PCWP by 3.5 mmHg (95% CI −6.6 to −0.4, *p* = 0.029) and peak-exercise PCWP by 5.7 mmHg (95% CI −10.8 to −0.7, *p* = 0.027), measured by invasive high-fidelity micromanometry under double-blind conditions. Exercise right atrial pressure fell by 4.1 mmHg (95% CI −7.2 to −1.0, *p* = 0.011) and exercise mean pulmonary artery pressure by 5.7 mmHg (95% CI −10.7 to −0.8, *p* = 0.024), alongside reductions in plasma volume of 285 mL (95% CI −510 to −60, *p* = 0.014) and in body weight of 3.5 kg (95% CI −5.9 to −1.1, *p* = 0.006 [35].

NT-proBNP. The direction of effect is inconsistent in HFpEF and the significant results are the more fragile ones. In CAMEO-DAPA, NT-proBNP was an exploratory outcome analyzed as a median change with a rank-sum test, and the difference (*p* = 0.037) reflects chiefly a rise in the placebo arm (+22 pg/mL, IQR 1 to 67) rather than a fall with dapagliflozin (−7 pg/mL, IQR −75 to 7) [35]. Ovchinnikov et al. reported a significant between-group difference (*p* = 0.038) in an open-label trial [36]. EXCEED found no overall effect (−118.9 pg/mL, 95% CI −278.4 to 40.6, *p* = 0.14), with a reduction confined to a post hoc subgroup of 16 participants whose baseline NT-proBNP was 400 pg/mL or above (−690.2 pg/mL, 95% CI −1224.7 to −155.7, *p* = 0.016) [37]. CANDLE did not meet its prespecified non-inferiority primary endpoint for NT-proBNP (ratio of percentage change 0.48, 95% CI 0.13 to 1.59, *p* = 0.226), and in its post hoc LVEF ≥ 50% subgroup the difference was −58.3 pg/mL (95% CI −127.6 to 11.0, *p* = 0.098), which is not statistically significant [34]. Taken together these results are compatible with a reduction in NT-proBNP that is larger when baseline concentrations are higher, but they do not clearly establish one. 

### 3.6. Synthesis by SGLT2 Inhibitor Agent

Empagliflozin was studied in six publications from five trials. The estimates are heterogeneous and their magnitude tracks study design: EMPA-TROPISM reported the largest volumetric changes but published no adjusted between-group estimate; SUGAR-DM-HF reported prespecified, adjusted reductions in both volume indices with no change in EF or strain; the Empire HF substudies reported volume, mass and atrial volume reductions that were not prespecified, and a neutral haemodynamic primary endpoint [24,25,26]; EMPA-VISION was neutral for its primary endpoint in both cohorts, with exploratory reductions in LVM in the HFrEF cohort only; and in HFpEF, Ovchinnikov et al. reported diastolic improvements in an open-label trial while EMPA-VISION HFpEF showed no change [36].

Dapagliflozin was studied in four trials. Fu et al. reported reductions in both volumes and an increase in EF [32]; DAPA-ECHO reported improvements in strain and E/e′ as within-group changes without an adjusted between-group estimate [30]; Reis et al. reported large interaction effects in which the control arm deteriorated markedly [31]; and REFORM, the only double-blind CMR trial of dapagliflozin, was neutral for every remodeling parameter [28]. In HFpEF, CAMEO-DAPA provided the review’s only prespecified and met invasive haemodynamic endpoint [35].

Ertugliflozin was studied in EFFORT, a multicenter double-blind trial in patients with LVEF 35% to <50%—a mixed reduced and mildly reduced EF population, not a pure HFmrEF cohort—who all had substantial functional mitral regurgitation. It met its primary endpoint, reducing effective regurgitant orifice area by 0.077 cm^2^, and reduced regurgitant volume by 11.2 mL (95% CI −16.1 to −6.3) and GLS by 1.44 percentage points (95% CI −2.42 to −0.46, *p* = 0.004), with no significant change in volume indices, EF or NT-proBNP. It is the trial at lowest risk of bias in the review and its strain estimate is the smallest of those reported [33].

Canagliflozin was studied in CANDLE, a mixed-ejection-fraction trial with an active comparator (glimepiride) that did not meet its prespecified non-inferiority primary endpoint for NT-proBNP (ratio 0.48, 95% CI 0.13 to 1.59, *p* = 0.226); the overall adjusted between-group difference was also non-significant (*p* = 0.087). Nominal significance was reached only in post hoc subgroups defined by baseline NT-proBNP, and the LVEF subgroup boundary used in reporting (50%) differs from the boundary used to stratify randomization (40%). Echocardiographic outcomes were also neutral overall, with no significant between-group differences in LVEF (+0.93 percentage points, 95% CI −1.15 to 3.02, *p* = 0.377) or E/e′ (−0.20, 95% CI −1.51 to 1.11, *p* = 0.759). Phenotype-stratified analyses were likewise neutral for both outcomes [34].

Ipragliflozin was studied in EXCEED, in which both co-primary diastolic endpoints and every secondary echocardiographic outcome were neutral; the only positive results were post hoc subgroup analyses in participants aged 70 years or over and in those with higher baseline NT-proBNP [37].

### 3.7. Synthesis by Imaging Modality

CMR-based trials (EMPA-TROPISM, SUGAR-DM-HF, REFORM and EMPA-VISION) and echocardiography-based trials produced broadly similar patterns rather than systematically different ones: within each modality, two trials reported volumetric reductions and two were neutral [24,25,28,29]. CMR offers greater measurement reproducibility for volumes and mass, which is why separate certainty assessments were prepared for each modality, but no trial compared modalities within the same participants and the two neutral CMR trials include the one whose primary endpoint was left ventricular end-systolic volume. It would therefore not be supportable to conclude from these data that CMR detects reverse remodeling that echocardiography misses. Strain findings differed by technique in a manner that is more readily explained by risk of bias than by physics: speckle-tracking echocardiography in open-label trials without stated reader blinding produced the largest effects, whereas CMR feature tracking, performed in double-blind trials, showed no change. Invasive studies (the Empire HF haemodynamic substudy and CAMEO-DAPA) provided the only direct measurements of filling pressure, and only CAMEO-DAPA met its prespecified haemodynamic endpoint.

## 4. Discussion

### 4.1. Principal Findings

This systematic review synthesized 14 publications from 13 randomized controlled trials (1232 participants analyzed) reporting imaging, biomarker and haemodynamic measures of cardiac remodeling during SGLT2 inhibitor treatment. The pattern of findings differed between phenotypes in the character of the changes reported: in reduced and mildly reduced EF, the changes reported were volumetric and strain-based, with inconsistent effects on LV volumes and mass and no consistent change in EF, whereas in preserved EF, LV volumes, EF, mass and strain were unchanged throughout and the only reproducible change was a reduction in filling pressures demonstrated invasively in one trial.

### 4.2. Reverse Remodeling in HFrEF

Reductions in left ventricular volumes were reported in half of the trials that measured them in reduced or mildly reduced EF, but the estimates are not compatible in magnitude and tone of the trials that were neutral include REFORM [28]; but the trial publishes no adjusted between-group estimate or confidence interval for any outcome, which limits how much weight its magnitude can bear.

Global longitudinal strain is a more sensitive marker of subclinical dysfunction than EF and independently predicts outcome [39,40], which is why an early and consistent strain signal would be biologically plausible. In these data, however, the size of the reported strain effect corresponds closely to the risk of bias of the trial reporting it: the only estimate derived from blinded core-laboratory analysis (EFFORT, −1.44 percentage points) is the smallest, the two largest estimates come from open-label trials that do not state whether strain readers were blinded, and strain measured by CMR feature tracking in double-blind trials did not change at all [33]. Strain analysis involves operator decisions about tracking and segment acceptance and is therefore susceptible to expectation when allocation is known. DAPA-ECHO additionally reported improvements in left atrial reservoir strain and right ventricular free-wall strain, which would be consistent with biventricular and atrial change, but reports them as within-group changes without an adjusted between-group estimate [30]. Overall, the evidence supports a modest improvement in longitudinal strain in reduced EF, but the available data do not permit a precise estimate of its magnitude.

Where volumes fell without a corresponding change in EF, as in SUGAR-DM-HF and the Empire HF echocardiographic substudy, the likeliest explanation is arithmetic: end-diastolic and end-systolic volumes fell in similar proportion, leaving their ratio unchanged. A longer treatment period might be required before a change in the ratio becomes detectable, and the follow-up of the contributing trials ranged from 12 weeks to 12 months [25,26].

### 4.3. Hemodynamic Unloading and Diastolic Improvement in HFpEF

The haemodynamic data from CAMEO-DAPA are the most secure findings in this review. Reductions in resting PCWP of 3.5 mmHg and in exercise PCWP of 5.7 mmHg, measured invasively under double-blind conditions against a prespecified and met primary endpoint [35], offer a plausible haemodynamic correlate of the clinical benefits observed in EMPEROR-Preserved [1] and DELIVER [4]. They occurred alongside plasma volume contraction and weight loss, which is consistent with a contribution from preload reduction. The estimates are nonetheless imprecise, their lower confidence bounds approach zero, they derive from a single trial of 38 participants, and no second invasive HFpEF trial exists against which to check them, so they establish that filling pressures fall rather than by how much.

Ovchinnikov et al. contributed the only exercise diastolic stress data in HFpEF, reporting improvements at rest in E/e′, e′ velocity and left atrial volume index, and during exercise in diastolic, atrial and chronotropic reserve [36]. These observations raise the possibility that SGLT2 inhibition improves the capacity to augment diastolic function under load, which would not be captured by resting measurements. Two considerations temper that reading. The trial was open-label and states that readers were blinded to data, without clear specification, leaving place for interpretation, and every outcome concerned is load-dependent in a setting where diuretic therapy could be adjusted with knowledge of allocation. More importantly, EXCEED—in which E/e′ and e′ were registered co-primary endpoints—found no effect, with a confidence interval that excludes the effect reported by Ovchinnikov et al. entirely [37]. The post hoc LVEF ≥ 50% subgroup of the mixed-ejection-fraction CANDLE trial was similarly neutral, with no significant between-group difference in E/e′ change [34]. Thus, the available evidence does not demonstrate a consistent effect of SGLT2 inhibition on echocardiographic indices of diastolic function in HFpEF, and the positive findings from Ovchinnikov et al. should be interpreted as hypothesis-generating pending confirmation in adequately powered, blinded trials with prespecified diastolic endpoints.

The absence of volumetric changes in HFpEF aligns with its pathophysiology, where ventricular dilatation is not the primary abnormality and in which a ceiling effect is expected when baseline EF is normal. The available evidence suggests that SGLT2 inhibitors may primarily reduce filling pressures and improve diastolic function rather than induce geometric reverse remodeling. However, this interpretation remains hypothesis-generating, as the HFpEF evidence is limited by small sample sizes, predominantly diabetic study populations, and methodological limitations, including incomplete analysis of randomized participants in the EMPA-VISION HFpEF cohort [29].

### 4.4. Role of EMPIRE-HF Substudies

The EMPIRE-HF trial merits special discussion as it generated complementary echocardiographic [26] and hemodynamic [27] data from overlapping populations, and its two substudies are counted here as one trial contributing 190 unique participants. The echocardiographic substudy reported reductions in LV volume indices, LVMi, and LAVI; however, these analyses were exploratory, as echocardiographic outcomes were not prespecified in the parent trial, and statistical significance was not maintained under an alternative prespecified adjustment. The hemodynamic substudy did not meet its prespecified primary endpoint, whereas reductions in PCWP and left ventricular chamber stiffness were observed in post hoc analyses. Collectively, these findings suggest that empagliflozin may promote both volumetric unloading and improved ventricular compliance in HFrEF. However, given the exploratory nature of several analyses and the absence of a significant between-group effect for some hemodynamic outcomes, these observations should be interpreted cautiously.

### 4.5. NT-proBNP Across Phenotypes

NT-proBNP responses to SGLT2 inhibition were heterogeneous across both heart failure phenotypes. In reduced ejection fraction, SUGAR-DM-HF reported an adjusted 28% reduction relative to placebo, whereas EFFORT was neutral, as was Empire HF, in which NT-proBNP was the prespecified primary endpoint. In preserved ejection fraction, favorable findings in CAMEO-DAPA and Ovchinnikov et al. were not reproduced in EXCEED, while the LVEF ≥ 50% subgroup of CANDLE also showed no significant difference (−58.3 pg/mL, 95% CI −127.6 to 11.0; *p* = 0.098) [25,33,34,35,36,37].

Taken together, these findings do not support a consistent reduction in NT-proBNP as a correlate of the cardiac effects of SGLT2 inhibition in either phenotype. Positive findings were accompanied by neutral results from trials with prespecified endpoints or lower risk of bias, and several favorable estimates arose from exploratory analyses, post hoc subgroups, or changes driven partly by deterioration in the comparator arm. Differences in the form of reporting—absolute change, percentage change, and log-transformed ratios—further limit direct comparison across trials. Accordingly, the certainty of evidence for NT-proBNP was rated very low in both reduced and preserved ejection fraction.

### 4.6. Justification for the Absence of Meta-Analysis

A quantitative meta-analysis was not performed. Three sources of heterogeneity applied across all outcomes and are described as such:

Heterogeneity of populations. The trials enrolled fundamentally different populations: non-diabetic reduced EF (EMPA-TROPISM, DAPA-ECHO, Reis et al.), diabetic reduced EF (SUGAR-DM-HF, REFORM, Fu et al.), a mixed reduced and mildly reduced cohort defined by functional mitral regurgitation (EFFORT), preserved EF with invasively confirmed exercise-induced elevation of filling pressure (CAMEO-DAPA), diabetic preserved EF (EXCEED, Ovchinnikov et al.) and mixed EF (CANDLE). Baseline LVEF ranged from 26% to 61%, type 2 diabetes prevalence from 0% to 100%, and follow-up from 12 weeks to 12 months.

Heterogeneity of what was measured and reported. Outcomes were reported as absolute or indexed volumes, as absolute or percentage change, and as between-group or within-group contrasts. Strain was measured by speckle-tracking echocardiography in some trials and CMR feature tracking in others, with opposite sign conventions in different publications. Five trials published no adjusted between-group estimate or confidence interval for any outcome, reporting only arm-level change scores or interaction *p* values.

Heterogeneity of endpoint status. In four trials, the prespecified primary endpoint was not met, and the remodeling outcomes synthesized here are explicitly labeled exploratory, post hoc or not prespecified. Pooling exploratory outcomes of neutral trials with prespecified outcomes of positive trials would produce a summary estimate whose susceptibility to selective reporting cannot be quantified.

An outcome-specific reasoning is offered in the Supplementary Material Table S9.

For these reasons a SWiM-compliant narrative synthesis based on the direction, magnitude and precision of randomized between-group treatment effects was the most appropriate approach [19].

### 4.7. Comparison with Previous Meta-Analyses

Three previous meta-analyses pooled remodeling outcomes across heart failure phenotypes. Fan et al. (2023) pooled 15 RCTs (*n* = 1343) and reported significant improvements in LVESV, LVEF, LVMi, E/e′ and LAVi [17]; Carluccio et al. (2023) pooled 9 RCTs (*n* = 1385) and reported reductions in LVEDV, LVESV, LVMi and NT-proBNP [18]; and Savage et al. (2024) evaluated 7 placebo-controlled RCTs (*n* = 657) and reported improvements in LVEDV, LVESV, LVEF and LVMi [41]. Zhang et al. reported that benefits in the overall heart failure population were driven mainly by reduced EF, with LVMi and LAVI not significantly improved in HFpEF which is directionally consistent with the pattern described here [42]. Unlike previous meta-analyses, this review synthesized the evidence according to heart failure phenotype and, where appropriate, imaging modality, while prioritizing randomized between-group treatment effects. This approach was chosen because of substantial clinical and methodological heterogeneity across the available studies, including differences in patient populations, imaging techniques, outcome definitions, follow-up duration, and the reporting of treatment effects. Although this strategy does not provide pooled effect estimates, it facilitates interpretation of the available evidence within clinically comparable settings and highlights the current limitations of the randomized imaging literature.

### 4.8. Strengths and Limitations

Strengths of this review include prospective PROSPERO registration, adherence to PRISMA 2020 and SWiM reporting guidelines, and the exclusive inclusion of randomized controlled trials. A phenotype-specific synthesis was performed by evaluating HF with reduced and mildly reduced EF and HFpEF separately, while integrating evidence from multimodality imaging, invasive hemodynamic assessment, and neurohormonal biomarkers to provide a comprehensive evaluation of cardiac remodeling. Treatment effects were synthesized using randomized between-group comparisons whenever available, and the certainty of evidence was assessed using structured RoB 2 and GRADE methodologies with phenotype- and modality-specific evidence profiles.

Limitations include (1) relatively small individual sample sizes (median ~70 patients); (2) risk of bias was influenced by five open-label trials with potential performance bias, absence of any statement that outcome assessors were blinded, exploratory or post hoc analyses, and incomplete reporting of adjusted between-group treatment effects; (3) substantial clinical and methodological heterogeneity—including differences in imaging modalities, outcome definitions, follow-up duration, mixed ejection-fraction populations, and the absence of dedicated HFmrEF randomized trials—limited phenotype-specific inference and precluded quantitative meta-analysis; (4) HFpEF evidence derives from four dedicated trials supplemented by post hoc phenotype-specific data from CANDLE, limiting generalizability and invasive hemodynamic data derive from only two studies; (5) EMPIRE-HF substudies share overlapping populations; (6) variable T2DM prevalence (0–100%), which may limit generalizability; (7) CANDLE used an active comparator (glimepiride) rather than placebo; (8) missing or individual-participant data could not be obtained from the original study authors; (9) eligibility was restricted to English-language publications, introducing potential language bias. These limitations should be considered when interpreting the findings and highlight the need for larger, adequately powered randomized trials using standardized imaging protocols and phenotype-specific analyses.

## 5. Conclusions

This systematic review of 14 publications from 13 randomized controlled trials identified distinct patterns of cardiac remodeling associated with SGLT2 inhibitor therapy across heart failure phenotypes. In studies enrolling patients with reduced or mildly reduced EF, treatment was associated primarily with reductions in LV volumes, while changes in LVM, EF, and myocardial deformation were less consistent. In studies of preserved EF, consistent left ventricular structural remodeling was not observed, whereas improvements were reported predominantly in cardiac filling pressures and other markers of diastolic function. The findings of this review should be interpreted as descriptive and hypothesis-generating. Although distinct patterns of cardiac remodeling were observed across heart failure phenotypes, the available evidence is limited by the small number of randomized studies, substantial clinical and methodological heterogeneity, and the overall low certainty of evidence. Consequently, these observations should be interpreted as potential phenotype-related differences that warrant further investigation. Future adequately powered, phenotype-specific randomized trials with prespecified remodeling endpoints, standardized imaging protocols, blinded image analysis and longer follow-up are needed to clarify the structural and functional effects of SGLT2 inhibitors and their relationship with clinical outcomes.

## Figures and Tables

**Table 1:** GRADE assessment. Certainty was assessed for every outcome, with separate evidence profiles by heart failure phenotype and, where both modalities contributed, by imaging modality. k—number of contributing studies; *n*—participants analyzed. The full rationale for every domain judgment is given in Supplementary Table S3b.

Outcome	Phenotype	Modality	k	*n*	Risk of Bias	Inconsistency	Indirectness	Imprecision	Publication Bias	Certainty	Summary of Effect
LVEDV/LVEDVi	Reduced EF (LVEF ≤ 40% and mixed <50% cohorts)	CMR	4	263	serious	serious	serious	serious	cannot be assessed	Very low	Direction inconsistent. Reduction of roughly 8 to 24 mL (or 8 mL/m^2^ indexed) in two trials, no change in two others.
LVEDV/LVEDVi	Reduced EF (LVEF ≤ 40% and mixed <50% cohorts)	Echo	4	466	serious	serious	serious	serious	cannot be assessed	Very low	Direction inconsistent. Reduction of about 5 to 6 mL or mL/m^2^ in two trials, no change in two.
LVESV/LVESVi	Reduced EF (LVEF ≤ 40% and mixed <50% cohorts)	CMR	3	228	serious	serious	serious	serious	cannot be assessed	Very low	Direction inconsistent. Reduction in two trials, no change in the one trial for which this was the prespecified primary endpoint.
LVESV/LVESVi	Reduced EF (LVEF ≤ 40% and mixed <50% cohorts)	Echo	4	466	serious	serious	serious	serious	cannot be assessed	Very low	Direction inconsistent.
LVEF	Reduced EF (LVEF ≤ 40% and mixed <50% cohorts)	CMR and echo	8	640	serious	very serious	serious	not serious	cannot be assessed	Very low	Probably little or no average effect on EF, with one strongly discrepant trial.
GLS	Reduced EF (LVEF ≤ 40% and mixed <50% cohorts)	CMR feature tracking	2	127	serious	not serious	not serious	serious	cannot be assessed	Low	Probably little or no effect on CMR-derived longitudinal strain.
GLS	Reduced EF (LVEF ≤ 40% and mixed <50% cohorts)	Speckle-tracking echo	4	446	very serious	serious	serious	serious	cannot be assessed	Very low	Longitudinal strain may improve modestly, but the effect size is strongly confounded with risk of bias, and the single low-risk-of-bias estimate is the smallest effect.
LV mass/LVMi	Reduced EF (LVEF ≤ 40% and mixed <50% cohorts)	CMR	4	263	serious	serious	serious	serious	cannot be assessed	Very low	Direction inconsistent. Reduction in two of four CMR trials.
LAVi	Reduced EF (LVEF ≤ 40% and mixed <50% cohorts)	Echo	4	446	very serious	serious	serious	serious	cannot be assessed	Very low	Left atrial volume index may fall, but no estimate is both precise and free of serious bias.
E/e-prime	Reduced EF (LVEF ≤ 40% and mixed <50% cohorts)	Echo	2	128	very serious	not serious	serious	very serious	cannot be assessed	Very low	Very uncertain. E/e-prime may fall, but the evidence comes only from unblinded trials without between-group estimates.
PCWP	Reduced EF (LVEF ≤ 40%)	Invasive right heart catheterization	1	66	very serious	not applicable	not serious	serious	cannot be assessed	Very low	Very uncertain. The prespecified haemodynamic primary endpoint was neutral; the reported PCWP reduction derives from a post hoc pooled analysis.
NT-proBNP	Reduced EF (LVEF ≤ 40% and mixed <50% cohorts)	Central laboratory assay	6	588	serious	serious	serious	not serious	cannot be assessed	Very low	Direction inconsistent. NT-proBNP fell in three of six reports, including the one trial at low risk of bias with a prespecified adjusted estimate, but
LVEDV/LVEDVi	Preserved EF (LVEF ≥ 50%)	CMR and echo	3	162	very serious	not serious	serious	serious	cannot be assessed	Very low	Probably little or no effect on left ventricular volumes in HFpEF, but the certainty is very low.
LVEF	Preserved EF (LVEF ≥ 50%)	CMR and echo	4	306	very serious	not serious	serious	serious	cannot be assessed	Very low	Probably little or no effect.
GLS	Preserved EF (LVEF ≥ 50%)	CMR feature tracking and speckle tracking	2	94	very serious	not serious	serious	very serious	cannot be assessed	Very low	No evidence of improvement in longitudinal strain in HFpEF. The previously reported favorable trend rests on a direction error.
LV mass/LVMi	Preserved EF (LVEF ≥ 50%)	CMR and echo	3	162	very serious	not serious	serious	serious	cannot be assessed	Very low	Probably little or no effect overall; an isolated subgroup finding in older patients.
LAVi	Preserved EF (LVEF ≥ 50%)	Echo	2	138	very serious	serious	serious	serious	cannot be assessed	Very low	Left atrial volume index may fall in HFpEF, but the evidence rests on one open-label trial.
E/e-prime	Preserved EF (LVEF ≥ 50%)	Echo	3	259	very serious	very serious	serious	serious	cannot be assessed	Very low	Highly uncertain. The one trial in which E/e-prime was a prespecified co-primary endpoint found no effect.
PCWP	Preserved EF (LVEF ≥ 50%)	Invasive exercise hemodynamics	1	38	not serious	not applicable	not serious	serious	cannot be assessed	Low	Dapagliflozin probably reduces PCWP at rest by about 3.5 mmHg and during exercise by about 5.7 mmHg in HFpEF. This is the most secure single finding i
NT-proBNP	Preserved EF (LVEF ≥ 50%)	Central laboratory assay	4	325	very serious	serious	serious	serious	cannot be assessed	Very low	Direction inconsistent and very uncertain. NT-proBNP may fall, but the significant results are exploratory or post hoc.

**Table 2:** Characteristics of Included Randomized Controlled Trials.

#	Study (Year)	Acronym	Design	n (SGLT2i/Ctrl)	HF Phenotype	EF Criteria	SGLT2i (Dose)	Imaging	Duration	T2DM (%)
1	Santos-Gallego (2021) [24]	EMPA-TROPISM	DB, PC, SC	84 (42/42)	Mixed reduced/mildly reduced EF	LVEF < 50% (mixed: admits HFrEF and HFmrEF)	Empagliflozin 10 mg	CMR	6 mo	0
2	Lee (2021) [25]	SUGAR-DM-HF	DB, PC, MC	105 (52/53)	HFrEF	LVEF ≤ 40%	Empagliflozin 10 mg	CMR	36 wk	78
3	Omar (2021) [26]	EMPIRE-HF echo	DB, PC, MC	190 (95/95)	HFrEF	LVEF ≤ 40%	Empagliflozin 10 mg	Echo	12 wk	13
4	Omar (2020) [27]	EMPIRE-HF hemo	DB, PC, SC	70 (35/35)	HFrEF	LVEF ≤ 40%	Empagliflozin 10 mg	Echo + RHC	12 wk	17
5	Singh (2020) [28]	REFORM	DB, PC, SC	56 (28/28)	Reduced EF, threshold not stated	Documented reduced LVEF; no numeric threshold reported	Dapagliflozin 10 mg	CMR	12 mo	100
6	Hundertmark (2023) [29]	EMPA-VISION	DB, PC, SC	72 (35/37)	HFrEF cohort and HFpEF cohort, analyzed separately	LVEF ≤ 40%/LVEF ≥ 50%	Empagliflozin 10 mg	CMR	12 wk	13
7	Pastore (2024) [30]	DAPA-ECHO	OL, SC	88 (44/44)	Mixed reduced/mildly reduced EF (62% HFrEF, 38% HFmrEF)	LVEF < 50% (mixed)	Dapagliflozin 10 mg	STE	6 mo	0
8	Reis (2022) [31]	DAPA-peakVO2	OL, SC	40 (20/20)	Mixed reduced/mildly reduced EF	LVEF < 50% (mixed: admits HFmrEF)	Dapagliflozin 10 mg	Echo	6 mo	0
9	Fu (2023) [32]	--	DB, PC, SC	60 (30/30)	HFrEF	EF ≤ 40%	Dapagliflozin 10 mg	Echo	12 mo	100
10	Kang (2024) [33]	EFFORT	DB, PC, MC	128 (63/65)	Mixed reduced/mildly reduced EF	LVEF 35% to <50% (spans HFrEF 35–40% and HFmrEF 41–49%)	Ertugliflozin	Echo	12 mo	13
11	Tanaka (2020) [34]	CANDLE	OL (PROBE), MC	233 (113/120)	Mixed EF—assigned to neither phenotype	Any LVEF (mean 57.6 ± 14.6%; 71% LVEF ≥ 50%); randomization stratified at 40%	Canagliflozin 100 mg	Echo	24 wk	100
12	Borlaug (2023) [35]	CAMEO-DAPA	DB, PC, SC	38 (21/17)	HFpEF	LVEF ≥ 50%	Dapagliflozin 10 mg	Echo + RHC	24 wk	18
13	Ovchinnikov (2025) [36]	--	OL, SC	70 (35/35)	HFpEF	LVEF ≥ 50%	Empagliflozin 10 mg	Echo (rest + ex)	6 mo	100
14	Akasaka (2022) [37]	EXCEED	OL, MC	68 (36/32)	HFpEF	LVEF ≥ 50%	Ipragliflozin 50 mg	Echo	24 wk	100

DB, double-blind; OL, open-label; PC, placebo-controlled; SC, single-center; MC, multicenter; PROBE, prospective randomized open blinded-endpoint; RHC, right heart catheterization; STE, speckle-tracking echocardiography. Phenotypes are classified according to ESC/AHA HF definitions and cohorts spanning more than one category are identified as mixed and are not assigned to a single phenotype. Studies 3 and 4 are two reports of one trial (Empire HF, NCT03198585) with overlapping populations, counted once (*n* = 190) in the participant total. Study 6 (EMPA-VISION, NCT03332212) enrolled separate HFrEF (*n* = 36 randomized, 35 analyzed) and HFpEF (*n* = 36 randomized, 24 analyzed) cohorts, analyzed separately. Randomized and analyzed denominators differ in several trials and are reported separately in Supplementary Table S1.

## Data Availability

The original contributions presented in this study are included in the article/Supplementary Materials. Further in-quiries can be directed to the corresponding author.

## Supplementary Materials

The following supporting information can be downloaded at: https://www.mdpi.com/article/10.3390/biomedicines14091961/s1, Table S1: detailed study characteristics, ejection-fraction inclusion ranges and phenotype transparency; Table S2: randomized between-group treatment effects by outcome and study; Table S3: GRADE evidence profiles by outcome, phenotype and imaging modality; Table S3b, GRADE appendix with the full rationale for every domain judgement; Table S4: discrepancy log; Table S5: Prisma S Checklist; Table S6a: search appendix with complete line-by-line strategies for PubMed, Embase and Scopus; Table S6b: citation searching log; Table S6c, validation table of retrieval by database; Table S7: Cohen’s kappa; Table S8: RoB 2 signaling-question responses and justifications; Table S9: Justification for the Absence of Meta-Analysis- outcome specific reasoning; Table S10: Study-publication map.

## Abbreviations

CI	confidence interval
CMR	cardiac magnetic resonance
Cochrane RoB 2	Cochrane Risk of Bias 2 tool
EF	ejection fraction
GLS	global longitudinal strain
GRADE	Grading of Recommendations Assessment, Development and Evaluation
GWE	global work efficiency
GWI	global work index
HF	heart failure
HFmrEF	heart failure with mildly reduced ejection fraction
HFpEF	heart failure with preserved ejection fraction
HFrEF	heart failure with reduced ejection fraction
LA	left atrial
LAV	left atrial volume
LAVI	left atrial volume index
LV	left ventricular
LVEDV	left ventricular end-diastolic volume
LVEDVi	left ventricular end-diastolic volume index
LVEF	left ventricular ejection fraction
LVESV	left ventricular end-systolic volume
LVESVi	left ventricular end-systolic volume index
LVMi	left ventricular mass index
NHE1	sodium-hydrogen exchanger 1
NHE3	sodium-hydrogen exchanger 3
NT-proBNP	N-terminal pro-B-type natriuretic peptide
PALS	peak atrial longitudinal strain
PCWP	pulmonary capillary wedge pressure
PICO	Population, Intervention, Comparator, Outcome
PRISMA	Preferred Reporting Items for Systematic Reviews and Meta-Analyses
RCT	randomized controlled trial
STE	speckle-tracking echocardiography
SGLT2	sodium-glucose cotransporter 2
SGLT2i	sodium-glucose cotransporter 2 inhibitor
SWiM	Synthesis Without Meta-analysis
T2DM	type 2 diabetes mellitus
