# Supplementary material for: Imaging and Hemodynamic Mechanisms of Cardiac Remodeling with SGLT2 Inhibitors Across Heart Failure Phenotypes: A Systematic Review
Source: Biomedicines. 2026 Aug 31;14(9):1961. doi: 10.3390/biomedicines14091961 (PMC13604603; doi:10.3390/biomedicines14091961)
Supplement: Supplementary file 1 [file biomedicines-14-01961-s001.zip › biomedicines-4458284-supplementary.pdf]

Supplementary Material

Imaging and Haemodynamic Mechanisms of Cardiac Remodelling With SGLT2 Inhibitors Across Heart Failure Phenotypes: A Systematic Review

Contents

Item	Content
Table S1	Detailed study characteristics, ejection-fraction inclusion ranges and phenotype transparency
Table S2	Randomised between-group treatment effects by outcome and study
Table S3	GRADE evidence profiles by outcome, phenotype and imaging modality
Table S3b	GRADE appendix: full written rationale for every domain judgement
Table S4	Discrepancy log: all independent reviewer disagreements before consensus
Figure S5	Prisma S Checklist
Table S6a	Search appendix: complete line-by-line strategies for PubMed, Embase and Scopus
Table S6b	Citation searching log
Table S6c	Validation table: retrieval of each included study by each database
Table S7	Cohen's kappa
Table S8	RoB 2 signalling-question responses and written justifications
Table S9	Justification for the Absence of Meta-Analysis- outcome specific reasoning
Table S10	Study-publication map

Table S1. Detailed study characteristics, ejection-fraction inclusion ranges and phenotype transparency

Every study is classified according to contemporary ESC/AHA definitions (HFrEF LVEF ≤ 40%; HFmrEF LVEF 41–49%; HFpEF LVEF ≥ 50%). Mixed cohorts are identified explicitly. "N randomised" and "N analysed" are given separately because several trials analysed substantially fewer participants than they randomised.

Study	First author, year	Design	SGLT2 inhibitor	Comparator	Imaging modality	Duration	N randomised (arms)	N analysed (arms)	EF inclusion range	Baseline LVEF (%) intervention / control	Predominant phenotype	Mixed cohort?	Phenotype-specific analyses available?	Prespecified primary endpoint and whether it was met
EMPA-TROPISM	Santos-Gallego 2021	Double-blind, placebo-controlled, single-centre	Empagliflozin 10 mg	Placebo	CMR	6 months	84 (42/42)	80 (~40/40)	LVEF < 50%	36.2 / 36.5	HFrEF	Yes - LVEF < 50% admits both HFrEF (≤40%) and HFmrEF (41-49%)	No	Change in LVEDV and LVESV (CMR)
SUGAR-DM-HF	Lee 2021	Double-blind, placebo-controlled, multicentre	Empagliflozin 10 mg	Placebo	CMR	36 weeks	105 (52/53)	92 (42/50)	LVEF ≤ 40%	31.7 / 33.0	HFrEF	No	n/a	Co-primary: LVESVi and LV GLS (CMR)
Empire HF (echocardiographic substudy)	Omar 2021	Double-blind, placebo-controlled, multicentre; exploratory post hoc substudy	Empagliflozin 10 mg	Placebo	Echo	12 weeks	190 (95/95)	186 (94/92)	LVEF ≤ 40%	29 (overall)	HFrEF	No	n/a	Parent trial primary endpoint was NT-proBNP; echocardiographic measures were NOT prespecified
Empire HF (haemodynamic substudy)	Omar 2020	Double-blind, placebo-controlled, single-centre substudy	Empagliflozin 10 mg	Placebo	Echo + right heart catheterisation	12 weeks	70 (35/35)	66 (33/33)	LVEF ≤ 40%	26 (overall)	HFrEF	No	n/a	Peak-exercise PCWP/cardiac index ratio - NEUTRAL (0.13; 95% CI -1.60 to 1.34; P=0.86)
REFORM	Singh 2020	Double-blind, placebo-controlled, single-centre	Dapagliflozin 10 mg	Placebo	CMR	12 months	56 (28/28)	56 (28/28 (ITT, single mean imputation))	Documented reduction in LVEF by echocardiography; no numeric threshold stated in the report	Not reported in the primary report	HFrEF	Unclear - EF entry threshold not stated	n/a	Change in LVESV (CMR)
EMPA-VISION (HFrEF cohort)	Hundertmark 2023	Double-blind, placebo-controlled, single-centre mechanistic trial	Empagliflozin 10 mg	Placebo	CMR + 31P-MRS	12 weeks	36 (17/19)	35 (17/18 (per protocol))	LVEF ≤ 40%	36.8 / 39.3	HFrEF	No	Yes - HFrEF and HFpEF cohorts analysed separately	Cardiac PCr/ATP ratio - NEUTRAL; all CMR outcomes were EXPLORATORY
EMPA-VISION (HFpEF cohort)	Hundertmark 2023	Double-blind, placebo-controlled, single-centre mechanistic trial	Empagliflozin 10 mg	Placebo	CMR + 31P-MRS	12 weeks	36 (18/18)	24 (13/11 (per protocol))	LVEF ≥ 50%	52.6 / 59.1 (marked baseline imbalance)	HFpEF	No	Yes	Cardiac PCr/ATP ratio - NEUTRAL; all CMR outcomes were EXPLORATORY
DAPA-ECHO	Pastore 2024	Open-label, single-centre	Dapagliflozin 10 mg	Optimal medical therapy without an SGLT2 inhibitor	Speckle-tracking echo	6 months	88 (44/44)	88 (44/44)	LVEF < 50% (62% HFrEF, 38% HFmrEF)	37 (overall)	HFrEF + HFmrEF	Yes - explicitly mixed reduced and mildly reduced EF	Partly - subgroup analyses by HFrEF/HFmrEF reported	Significant change in LV GLS, LA reservoir strain and RV free-wall strain
Reis 2022 (dapagliflozin and exercise	Reis 2022	Open-label, single-centre	Dapagliflozin 10 mg	Continuation of heart failure medication	Echo + CPET	6 months	40 (20/20)	40 (20/20)	LVEF < 50%	33.5 / 34.5	HFrEF	Yes - LVEF < 50% admits HFmrEF	No	6-month change in peak oxygen uptake

capacity)														
Fu 2023	Fu 2023	Double-blind, placebo-controlled, single-centre	Dapagliflozin 10 mg	Placebo	Echo	12 months	60 (30/30)	60 (30/30)	LVEF ≤ 40%	Not reported	HFrEF	No	n/a	Change in LVEF
EFFORT	Kang 2024	Double-blind, placebo-controlled, multicentre	Ertugliflozin 5 mg	Placebo	Echo	12 months	128 (65/63)	128 (65/63)	LVEF 35% to < 50%	41.9 / 42.4	Mixed reduced and mildly reduced EF	Yes - spans HFrEF (35-40%) and HFmrEF (41-49%); NOT a pure HFmrEF cohort	No	Change in effective regurgitant orifice area of functional mitral regurgitation
CANDLE	Tanaka 2020	Open-label, blinded-endpoint (PROBE), multicentre	Canagliflozin 100 mg	Glimepiride (active comparator)	Echo	24 weeks	233 (113/120)	233 (113/120)	Any LVEF (stratified <40% / ≥40% at screening)	57.6 +/- 14.6 overall; 71% LVEF ≥ 50%	Mixed ejection fraction	Yes - mixed-EF population; predominantly preserved EF	Post hoc subgroups by LVEF < 50% / ≥ 50% for NT-proBNP, E/e`, LVEF	Ratio of percentage change in NT-proBNP at 24 weeks - NON-INFERIORITY NOT MET
CAMEO-DAPA	Borlaug 2023	Double-blind, placebo-controlled, single-centre	Dapagliflozin 10 mg	Placebo	Echo + invasive exercise haemodynamics	24 weeks	43 (23/20)	38 (21/17)	LVEF ≥ 50%	Preserved by protocol	HFpEF	No	n/a	Change in PCWP at rest and during exercise - MET (likelihood ratio test P<0.001)
Ovchinnikov 2025	Ovchinnikov 2025	Open-label, single-centre	Empagliflozin 10 mg	Standard therapy without an SGLT2 inhibitor	Echo at rest and during exercise	6 months	70 (35/35)	70 (35/35)	LVEF ≥ 50%	Preserved by protocol	HFpEF	No	n/a	Change in 6-minute walk distance (not an imaging outcome)
EXCEED	Akasaka 2022	Open-label, multicentre	Ipragliflozin 50 mg	Conventional treatment	Echo	24 weeks	73 (40/33)	68 (36/32)	LVEF ≥ 50%	60.9 / 60.4	HFpEF	No	n/a	Co-primary: change in E/e-prime and e-prime - BOTH NEUTRAL

Participants analysed across the 13 unique trials total 1232 (Empire HF counted once, n = 190). The randomised total across the same 13 trials is 1242.

Table S2. Randomised between-group treatment effects by outcome and study

For every outcome it reports the direction of effect, the point estimate, its 95% confidence interval, the exact p value, the type of contrast the source publication reported, the imaging modality, follow-up duration, heart failure phenotype and the risk-of-bias judgement for that outcome stratum.

Contrast codes: BG = adjusted between-group treatment effect reported by the authors; BGd = between-group difference derived by the review team from arm-level change scores; WG = within-group change in the intervention arm only, no between-group estimate published; INT = treatment-by-time interaction p value only, no point estimate published.

Effect direction (↓ ↑ ↔) is interpreted clinically, not by numeric sign. For strain a more negative value denotes improvement, so the sign convention of each source was checked individually.

Study	Outcome	Effect direction	Point estimate	95% CI	p value	Contrast	Unit	Imaging modality	Follow-up	Phenotype	RoB 2 (this outcome stratum)	Notes on the extracted value
EMPA-TROPISM	LVEDV	↓ / ↑ favours SGLT2i	-23.6	-34.9 to -12.3	<0.001	BGd	mL	CMR	6 months	HFrEF	Some concerns	Derived from arm-level change scores (-25.1 +/- 26.0 vs -1.5 +/- 25.4). No adjusted between-group estimate published.
EMPA-TROPISM	LVESV	↓ / ↑ favours SGLT2i	-26.1	-35.4 to -16.8	<0.001	BGd	mL	CMR	6 months	HFrEF	Some concerns	Derived from arm-level change scores (-26.6 +/- 20.5 vs -0.5 +/- 21.9).
EMPA-TROPISM	LVEF	↓ / ↑ favours SGLT2i	6.1	4.3 to 7.9	<0.001	BGd	percentage points	CMR	6 months	HFrEF	Some concerns	Derived from arm-level change scores (+6.0 +/- 4.2 vs -0.1 +/- 3.9).
EMPA-TROPISM	LV mass	↓ / ↑ favours SGLT2i	-21.9	-32.6 to -11.2	<0.001	BGd	g	CMR	6 months	HFrEF	Some concerns	Derived from arm-level change scores (-17.8 +/- 31.9 vs +4.1 +/- 13.4). LV mass, not indexed.
EMPA-TROPISM	NT-proBNP	↓ / ↑ favours SGLT2i	—	—	0.01	WG	% change	CMR	6 months	HFrEF	Some concerns	Reported as -11.5% with empagliflozin vs +8.5% with placebo; no adjusted between-group estimate.
SUGAR-DM-HF	LVESVi	↓ / ↑ favours SGLT2i	-6	-10.8 to -1.2	0.015	BG	mL/m2	CMR	36 weeks	HFrEF	Some concerns	Co-primary outcome.
SUGAR-DM-HF	LVEDVi	↓ / ↑ favours SGLT2i	-8.2	-13.7 to -2.6	0.004	BG	mL/m2	CMR	36 weeks	HFrEF	Some concerns	Secondary outcome.
SUGAR-DM-HF	LVEF	↔ no difference	0.3	-1.7 to 2.3	0.75	BG	percentage points	CMR	36 weeks	HFrEF	Some concerns	
SUGAR-DM-HF	LVMi	↔ no difference	-1.9	-4.7 to 0.8	0.17	BG	g/m2	CMR	36 weeks	HFrEF	Some concerns	
SUGAR-DM-HF	LAVi	↔ no difference	-2.4	-6.5 to 1.8	0.26	BG	mL/m2	CMR	36 weeks	HFrEF	Some concerns	
SUGAR-DM-HF	GLS	↔ no difference	0.35	-0.25 to 0.95	0.25	BG	percentage points	CMR	36 weeks	HFrEF	Some concerns	CMR feature tracking, not speckle-tracking echocardiography. Co-primary outcome. Positive value = less negative strain = no improvement.
SUGAR-DM-HF	NT-proBNP	↓ / ↑ favours SGLT2i	-28	-47 to -2	0.038	BG	% change	CMR	36 weeks	HFrEF	Low	Ratio of change on the log scale. The absolute arm-level changes (-266 vs +224 pg/mL) are NOT the between-group estimate.
Empire HF (echocardiographic substudy)	LVESVi	↓ / ↑ favours SGLT2i	-4.3	-8.5 to -0.1	0.04	BG	mL/m2	Echocardiography	12 weeks	HFrEF	High	Not prespecified in the parent trial.
Empire HF (echocardiographic substudy)	LVEDVi	↓ / ↑ favours SGLT2i	-5.5	-10.6 to -0.4	0.03	BG	mL/m2	Echocardiography	12 weeks	HFrEF	High	Not prespecified in the parent trial.
Empire HF (echocardiographic substudy)	LAVi	↓ / ↑ favours SGLT2i	-2.5	-4.8 to -0.1	0.04	BG	mL/m2	Echocardiography	12 weeks	HFrEF	High	Not prespecified in the parent trial.
Empire HF (echocardiographic substudy)	LVEF	↔ no difference	1.2	-1.2 to 3.6	0.32	BG	percentage points	Echocardiography	12 weeks	HFrEF	High	

Empire HF (echocardiographic substudy)	LVMi	↓ / ↑ favours SGLT2i	-9	-17.2 to -0.8	0.03	BG	g/m2	Echocardiography	12 weeks	HFrEF	High	
Empire HF (echocardiographic substudy)	GLS	↔ no difference	0.4	-0.3 to 1	0.31	BG	percentage points	Echocardiography	12 weeks	HFrEF	High	Speckle-tracking echocardiography. Positive value = less negative strain = no improvement.
Empire HF (echocardiographic substudy)	NT-proBNP	↔ no difference	—	—	NS	BG		Echocardiography	12 weeks	HFrEF	Low	Parent trial primary endpoint; no significant between-group reduction.
Empire HF (haemodynamic substudy)	PCWP (peak exercise / cardiac index, PRIMARY)	↔ no difference	0.13	-1.6 to 1.34	0.86	BG	mmHg/L/min/m2	Echocardiography + right heart catheterisation	12 weeks	HFrEF	High	PRIMARY ENDPOINT - neutral.
Empire HF (haemodynamic substudy)	PCWP (rest)	↔ no difference	-1.47	-3.86 to 0.82	0.23	BG	mmHg	Echocardiography + right heart catheterisation	12 weeks	HFrEF	High	
Empire HF (haemodynamic substudy)	PCWP (across all exercise loads)	↓ / ↑ favours SGLT2i	-2.4	-3.96 to -0.84	0.003	BG	mmHg	Echocardiography + right heart catheterisation	12 weeks	HFrEF	High	
Empire HF (haemodynamic substudy)	Cardiac index (across exercise loads)	↔ no difference	0.09	-0.14 to 0.32	0.448	BG	L/min/m2	Echocardiography + right heart catheterisation	12 weeks	HFrEF	High	
Empire HF (haemodynamic substudy)	Plasma volume	↓ / ↑ favours SGLT2i	-164.9	-236.14 to -93.65	<0.001	BG	mL	Echocardiography + right heart catheterisation	12 weeks	HFrEF	High	
Empire HF (haemodynamic substudy)	LV chamber stiffness (EDPVR beta)	↓ / ↑ favours SGLT2i	-7.5	-14.3 to -0.64	0.03	WG	beta coefficient	Echocardiography + right heart catheterisation	12 weeks	HFrEF	High	WITHIN-GROUP change in the empagliflozin arm. NO significant between-group treatment effect was demonstrated.
Empire HF (haemodynamic substudy)	Right atrial pressure (rest)	↔ no difference	0.36	-1.41 to 2.13	0.69	BG	mmHg	Echocardiography + right heart catheterisation	12 weeks	HFrEF	High	
REFORM	LVESV	↔ no difference	4.82	-13.28 to 22.93	0.594	BG	mL	CMR	12 months	HFrEF	Some concerns	Primary outcome - neutral.
REFORM	LVESVi	↔ no difference	2.49	-6.3 to 11.28	0.571	BG	mL/m2	CMR	12 months	HFrEF	Some concerns	
REFORM	LVEDV	↔ no difference	7.83	-15.05 to 30.7	0.495	BG	mL	CMR	12	HFrEF	Some	

									months		concerns	
REFORM	LVEDVi	↔ no difference	3.9	-7.05 to 14.85	0.478	BG	mL/m2	CMR	12 months	HFrEF	Some concerns	
REFORM	LVEF	↔ no difference	0.69	-3.32 to 4.69	0.732	BG	percentage points	CMR	12 months	HFrEF	Some concerns	Table 1 reports 0.69; the Results text reports 0.96.
REFORM	LVMi	↔ no difference	2.5	-3.95 to 8.95	0.44	BG	g/m2	CMR	12 months	HFrEF	Some concerns	
REFORM	LAVi	↔ no difference	-2.6	-9.67 to 4.48	0.464	BG	mL/m2	CMR	12 months	HFrEF	Some concerns	
EMPA-VISION (HFrEF cohort)	LVEDV	↔ no difference	-7	-21.98 to 8.85	0.39	BG	mL	CMR + 31P-MRS	12 weeks	HFrEF	High	Exploratory outcome.
EMPA-VISION (HFrEF cohort)	LVEF	↔ no difference	1.87	-1.6 to 5.4	0.29	BG	percentage points	CMR + 31P-MRS	12 weeks	HFrEF	High	Exploratory outcome.
EMPA-VISION (HFrEF cohort)	LV mass	↓ / ↑ favours SGLT2i	-9.65	-17.5 to -1.8	<0.05	BG	g	CMR + 31P-MRS	12 weeks	HFrEF	High	Exploratory outcome.
EMPA-VISION (HFrEF cohort)	LVMi	↓ / ↑ favours SGLT2i	-4.46	-8.42 to -0.5	<0.05	BG	g/m2	CMR + 31P-MRS	12 weeks	HFrEF	High	Exploratory outcome.
EMPA-VISION (HFrEF cohort)	GLS	↔ no difference	1.95	-0.6 to 4.5	0.12	BG	percentage points	CMR + 31P-MRS	12 weeks	HFrEF	High	CMR feature tracking. Positive value = strain became LESS negative with empagliflozin = no improvement.
EMPA-VISION (HFpEF cohort)	LVEDV	↔ no difference	-2.74	-20.44 to 14.95	0.75	BG	mL	CMR + 31P-MRS	12 weeks	HFpEF	High	Exploratory outcome.
EMPA-VISION (HFpEF cohort)	LVEF	↔ no difference	1.47	-2.89 to 5.83	0.49	BG	percentage points	CMR + 31P-MRS	12 weeks	HFpEF	High	Exploratory outcome.
EMPA-VISION (HFpEF cohort)	LV mass	↔ no difference	-0.41	-10.86 to 10.04	0.94	BG	g	CMR + 31P-MRS	12 weeks	HFpEF	High	Exploratory outcome.
EMPA-VISION (HFpEF cohort)	LVMi	↔ no difference	-1.18	-7.06 to 4.71	0.68	BG	g/m2	CMR + 31P-MRS	12 weeks	HFpEF	High	Exploratory outcome.
EMPA-VISION (HFpEF cohort)	GLS	↑ / ↓ favours comparator	2.18	-0.28 to 4.64	0.08	BG	percentage points	CMR + 31P-MRS	12 weeks	HFpEF	High	Longitudinal strain moved from -14.16% to -12.31% with empagliflozin and from -13.53% to -13.84% with placebo. The positive adjusted difference therefore denotes strain becoming LESS negative, i.e. numerically WORSE, with empagliflozin. It is not an improvement, and the result is non-significant.
DAPA-ECHO	LVEDVi	↔ no difference	-0.8	-8.5 to 6.8	0.83	WG	mL/m2	Speckle-tracking echocardiography	6 months	HFrEF + HFmrEF	Some concerns	Within-group change, dapagliflozin arm.
DAPA-ECHO	LVESVi	↔ no difference	-0.8	-6 to 6.1	0.79	WG	mL/m2	Speckle-tracking echocardiography	6 months	HFrEF + HFmrEF	Some concerns	Within-group change, dapagliflozin arm.
DAPA-ECHO	LVEF	↔ no difference	1.2	-1.6 to 4	0.38	WG	percentage points	Speckle-tracking echocardiography	6 months	HFrEF + HFmrEF	Some concerns	Within-group change, dapagliflozin arm.
DAPA-ECHO	LAVi	↔ no difference	-1	-4 to 1.9	0.49	WG	mL/m2	Speckle-tracking echocardiography	6 months	HFrEF + HFmrEF	High	Within-group change, dapagliflozin arm.

DAPA-ECHO	GLS	↓ / ↑ favours SGLT2i	-2.5	-1.8 to -0.4	<0.001	WG	percentage points	Speckle-tracking echocardiography	6 months	HFrEF + HFmrEF	High	Within-group change in the dapagliflozin arm (OMT arm -0.3, P=0.53). The trial reports a significant treatment-by-time effect (P<0.0001) from a linear mixed model but no adjusted between-group mean difference with a confidence interval. Negative value = more negative strain = improvement.
DAPA-ECHO	E/e-prime	↓ / ↑ favours SGLT2i	-1.7	-3.2 to -0.25	0.022	WG	ratio units	Speckle-tracking echocardiography	6 months	HFrEF + HFmrEF	High	Within-group change in the dapagliflozin arm (OMT arm +0.6, 95% CI -0.91 to 2.09, P=0.43). NOT a between-group contrast.
DAPA-ECHO	LA reservoir strain (PALS)	↓ / ↑ favours SGLT2i	5.12	3.5 to 6.7	<0.001	WG	percentage points	Speckle-tracking echocardiography	6 months	HFrEF + HFmrEF	High	Within-group change in the dapagliflozin arm (OMT arm +1.1, P=0.14).
DAPA-ECHO	NT-proBNP	↔ no difference	-560	—	NS	WG	pg/L	Speckle-tracking echocardiography	6 months	HFrEF + HFmrEF	Some concerns	Median change -560 pg/L with dapagliflozin vs -90 pg/L with OMT; not statistically significant.
Reis 2022 (dapagliflozin and exercise capacity)	LVEF	↔ no difference	1.7	—	0.512	INT	percentage points	Echocardiography + CPET	6 months	HFrEF	High	Derived difference in change (+3.4 vs +1.7). Time x treatment interaction P=0.512.
Reis 2022 (dapagliflozin and exercise capacity)	GLS	↓ / ↑ favours SGLT2i	-3.8	—	<0.001	INT	percentage points	Echocardiography + CPET	6 months	HFrEF	High	Derived difference in change (dapagliflozin -2.2 vs control +1.6). Interaction P<0.001. The apparent effect is driven partly by deterioration in the control arm.
Reis 2022 (dapagliflozin and exercise capacity)	E/e-prime	↓ / ↑ favours SGLT2i	-2.9	—	0.026	INT	ratio units	Echocardiography + CPET	6 months	HFrEF	High	Derived difference in change (dapagliflozin -1.4 vs control +1.5). Interaction P=0.026.
Reis 2022 (dapagliflozin and exercise capacity)	LAVi	↓ / ↑ favours SGLT2i	-13.1	—	<0.001	INT	mL/m2	Echocardiography + CPET	6 months	HFrEF	High	Derived difference in change (dapagliflozin -6.9 vs control +6.2). Interaction P<0.001. The control arm deteriorated markedly, which inflates the apparent between-group difference.
Reis 2022 (dapagliflozin and exercise capacity)	NT-proBNP	↓ / ↑ favours SGLT2i	-867.9	—	0.007	INT	pg/mL	Echocardiography + CPET	6 months	HFrEF	High	Derived difference in change (dapagliflozin -217.6 vs control +650.3). Interaction P=0.007.
Reis 2022 (dapagliflozin and exercise capacity)	LV end-diastolic DIAMETER	↓ / ↑ favours SGLT2i	-4.9	—	0.010	INT	mm	Echocardiography + CPET	6 months	HFrEF	High	A linear dimension, not a volume. LVEDV and LVESV were NOT reported.
Fu 2023	LVEF	↓ / ↑ favours SGLT2i	2.5	1 to 4.06	0.002	BG	percentage points	Echocardiography	12 months	HFrEF	Some concerns	Primary outcome.
Fu 2023	LVEDV	↓ / ↑ favours SGLT2i	-6	-8.07 to -3.87	<0.001	BG	mL	Echocardiography	12 months	HFrEF	Some concerns	
Fu 2023	LVESV	↓ / ↑ favours SGLT2i	-8.1	-11.07 to -5.14	<0.001	BG	mL	Echocardiography	12 months	HFrEF	Some concerns	
Fu 2023	LV end-diastolic DIAMETER	↓ / ↑ favours SGLT2i	-1.6	-2.67 to -0.62	0.002	BG	mm	Echocardiography	12 months	HFrEF	Some concerns	A linear dimension.
EFFORT	EROA of functional MR (PRIMARY)	↓ / ↑ favours SGLT2i	-0.077	—	<0.001	BG	cm2	Echocardiography	12 months	Mixed reduced and mildly reduced EF	Some concerns	Primary outcome.
EFFORT	Regurgitant volume	↓ / ↑ favours SGLT2i	-11.2	-16.1 to -6.3	0.009	BG	mL	Echocardiography	12 months	Mixed reduced	Some concerns	

										and mildly reduced EF		
EFFORT	GLS	↓ / ↑ favours SGLT2i	-1.44	-2.42 to -0.46	0.004	BG	percentage points	Echocardiography	12 months	Mixed reduced and mildly reduced EF	Some concerns	Speckle-tracking echocardiography. Negative value = more negative strain = improvement.
EFFORT	LAVi	↓ / ↑ favours SGLT2i	-6	-12.16 to 0.15	0.005	BG	mL/m2	Echocardiography	12 months	Mixed reduced and mildly reduced EF	Some concerns	The published 95% CI (-12.16 to 0.15) crosses zero although P is reported as 0.005. Reported here as published;
EFFORT	LVEDVi	↔ no difference	—	—	NS	BG	mL/m2	Echocardiography	12 months	Mixed reduced and mildly reduced EF	Low	No significant between-group difference reported.
EFFORT	LVESVi	↔ no difference	—	—	NS	BG	mL/m2	Echocardiography	12 months	Mixed reduced and mildly reduced EF	Low	No significant between-group difference reported.
EFFORT	LVEF	↔ no difference	—	—	NS	BG	percentage points	Echocardiography	12 months	Mixed reduced and mildly reduced EF	Low	No significant between-group difference reported.
EFFORT	NT-proBNP	↔ no difference	—	—	NS	BG	pg/mL	Echocardiography	12 months	Mixed reduced and mildly reduced EF	Low	No significant between-group difference reported.
CANDLE	NT-proBNP ratio of % change (PRIMARY)	↔ no difference	0.48	0.13 to 1.59	0.226	BG	ratio	Echocardiography	24 weeks	Mixed ejection fraction	High	PRIMARY ENDPOINT: prespecified non-inferiority margin of 1.1 was NOT met.
CANDLE	NT-proBNP (adjusted mean change, overall)	↔ no difference	-74.2	—	0.087	BGd	pg/mL	Echocardiography	24 weeks	Mixed ejection fraction	High	Derived from arm-level adjusted changes (-78.7 vs -4.5 pg/mL). Non-significant.
CANDLE	NT-proBNP (baseline ≥ 125 pg/mL subgroup)	↓ / ↑ favours SGLT2i	-115.4	—	0.047	BGd	pg/mL	Echocardiography	24 weeks	Mixed ejection fraction	High	Derived from arm-level adjusted changes (-121.5 vs -6.1 pg/mL). SUBGROUP analysis.
CANDLE	NT-proBNP (LVEF ≥ 50% subgroup)	↔ no difference	-58.3	-127.6 to 11	0.098	BGd	pg/mL	Echocardiography	24 weeks	Mixed ejection fraction	High	SUBGROUP analysis; non-significant. Previously described as "borderline" - that wording has been removed.
CANDLE	NT-proBNP (LVEF < 50% subgroup)	↔ no difference	-10	-204.2 to 224.3	0.926	BGd	pg/mL	Echocardiography	24 weeks	Mixed ejection fraction	High	SUBGROUP analysis.
CANDLE	LVEF	↔ no difference	0.93	−1.15 to 3.02	0.377	BG	percentage points	Echocardiography	24 weeks	Mixed ejection fraction	High	Between-group difference in change from baseline extracted from Supplementary Table S5 of the source publication. No statistically significant difference between treatment groups.
CANDLE	E/e-prime	↔ no difference	−0.20	−1.51 to 1.11	0.759	BG	ratio units	Echocardiography	24 weeks	Mixed ejection fraction	High	The reported effect is the between-group difference in change from baseline. Mean change from baseline: Canagliflozin −0.7 ± 3.8 (n=73) versus Glimepiride −0.5 ± 4.6 (n=86), yielding a group difference of −0.20 (95% CI −1.51 to 1.11; P=0.759).
CAMEO-DAPA	PCWP rest	↓ / ↑ favours SGLT2i	-3.5	-6.6 to -0.4	0.029	BG	mmHg	Echocardiography +	24 weeks	HFpEF	Some concerns	Component of the primary endpoint.

								invasive exercise haemodynamics				
CAMEO-DAPA	PCWP peak exercise	↓ / ↑ favours SGLT2i	-5.7	-10.8 to -0.7	0.027	BG	mmHg	Echocardiography + invasive exercise haemodynamics	24 weeks	HFpEF	Some concerns	Component of the primary endpoint.
CAMEO-DAPA	Right atrial pressure (exercise)	↓ / ↑ favours SGLT2i	-4.1	-7.2 to -1	0.011	BG	mmHg	Echocardiography + invasive exercise haemodynamics	24 weeks	HFpEF	Some concerns	Secondary endpoint; EXERCISE value.
CAMEO-DAPA	Mean pulmonary artery pressure (exercise)	↓ / ↑ favours SGLT2i	-5.7	-10.7 to -0.8	0.024	BG	mmHg	Echocardiography + invasive exercise haemodynamics	24 weeks	HFpEF	Some concerns	Secondary endpoint; EXERCISE value.
CAMEO-DAPA	Plasma volume	↓ / ↑ favours SGLT2i	-285	-510 to -60	0.014	BG	mL	Echocardiography + invasive exercise haemodynamics	24 weeks	HFpEF	Some concerns	
CAMEO-DAPA	Body weight	↓ / ↑ favours SGLT2i	-3.5	-5.9 to -1.1	0.006	BG	kg	Echocardiography + invasive exercise haemodynamics	24 weeks	HFpEF	Some concerns	
CAMEO-DAPA	NT-proBNP	↓ / ↑ favours SGLT2i	—	—	0.037	BG	pg/mL	Echocardiography + invasive exercise haemodynamics	24 weeks	HFpEF	Some concerns	EXPLORATORY outcome, Wilcoxon rank sum on median change: -7 pg/mL (IQR -75 to 7) with dapagliflozin versus +22 pg/mL (IQR 1 to 67) with placebo. The between-group difference chiefly reflects a RISE in the placebo arm rather than a fall with dapagliflozin.
Ovchinnikov 2025	E/e-prime (rest)	↓ / ↑ favours SGLT2i	-1.8	-2.6 to -1.1	<0.001	BG	ratio units	Echocardiography at rest and during exercise	6 months	HFpEF	High	Source reports the between-group difference as +1.8 (95% CI 1.1 to 2.6) with the sign convention "control minus empagliflozin"; re-expressed here as the effect of empagliflozin relative to control.
Ovchinnikov 2025	LAVi	↓ / ↑ favours SGLT2i	-3.4	-5.1 to -1.6	<0.001	BG	mL/m2	Echocardiography at rest and during exercise	6 months	HFpEF	High	Sign convention re-expressed as above.
Ovchinnikov 2025	LV GLS	↔ no difference	-0.5	-1.4 to 0.3	0.22	BG	percentage points	Echocardiography at rest and during	6 months	HFpEF	High	

								exercise				
Ovchinnikov 2025	LVMi	↔ no difference	-2.8	-7.9 to 2.3	0.28	BG	g/m2	Echocardiography at rest and during exercise	6 months	HFpEF	Some concerns	
Ovchinnikov 2025	6-minute walk distance (PRIMARY)	↓ / ↑ favours SGLT2i	13	1 to 25	0.033	BG	m	Echocardiography at rest and during exercise	6 months	HFpEF	High	PRIMARY ENDPOINT - a functional, not an imaging, outcome.
EXCEED	E/e-prime (CO-PRIMARY)	↔ no difference	-0.04	-1.3 to 1.2	0.95	BG	ratio units	Echocardiography	24 weeks	HFpEF	Some concerns	CO-PRIMARY ENDPOINT - neutral.
EXCEED	e-prime (CO-PRIMARY)	↔ no difference	-0.3	-0.9 to 0.3	0.33	BG	cm/s	Echocardiography	24 weeks	HFpEF	Some concerns	CO-PRIMARY ENDPOINT - neutral.
EXCEED	LVEF	↔ no difference	1.3	-1.4 to 4	0.34	BG	percentage points	Echocardiography	24 weeks	HFpEF	High	
EXCEED	LVEDV	↔ no difference	-4.9	-14.9 to 5.1	0.33	BG	mL	Echocardiography	24 weeks	HFpEF	High	
EXCEED	LVESV	↔ no difference	-1.7	-6.4 to 3.1	0.48	BG	mL	Echocardiography	24 weeks	HFpEF	High	
EXCEED	Left atrial volume (NOT indexed)	↔ no difference	-7.1	-14.4 to 0.2	0.06	BG	mL	Echocardiography	24 weeks	HFpEF	Some concerns	Absolute left atrial volume, NOT indexed to body surface area, and therefore not directly comparable with the LAVi values reported by other studies. Non-significant.
EXCEED	LVMi (subgroup aged ≥ 70 years)	↓ / ↑ favours SGLT2i	-19.9	-38 to -1.75	0.033	BG	g/m2	Echocardiography	24 weeks	HFpEF	High	SUBGROUP analysis (n=38).
EXCEED	NT-proBNP	↔ no difference	-118.9	-278.4 to 40.6	0.14	BG	pg/mL	Echocardiography	24 weeks	HFpEF	High	
EXCEED	NT-proBNP (baseline ≥ 400 pg/mL subgroup)	↓ / ↑ favours SGLT2i	-690.2	-1224.7 to -155.7	0.016	BG	pg/mL	Echocardiography	24 weeks	HFpEF	High	SUBGROUP analysis (n=16).

Table S3. GRADE evidence profiles by outcome, phenotype and imaging modality

Certainty was reassessed from first principles for every outcome. Evidence profiles are prepared separately for reduced and preserved ejection fraction, and separately for cardiac magnetic resonance and echocardiography wherever both modalities contribute, so that indirectness is not introduced by pooling fundamentally different populations or measurement techniques.

Outcome	Phenotype	Imaging modality	Studies (k)	Participants (n)	Risk of bias	Inconsistency	Indirectness	Imprecision	Publication bias	Certainty	Summary of effect
LVEDV / LVEDVi	Reduced EF (LVEF ≤40% and mixed <50% cohorts)	CMR	4	263	serious	serious	serious	serious	cannot be assessed	Very low	Direction inconsistent. Reduction of roughly 8 to 24 mL (or 8 mL/m2 indexed) in two trials, no change in two others.
LVEDV / LVEDVi	Reduced EF (LVEF ≤40% and mixed <50% cohorts)	Echocardiography	4	466	serious	serious	serious	serious	cannot be assessed	Very low	Direction inconsistent. Reduction of about 5 to 6 mL or mL/m2 in two trials, no change in two.
LVESV / LVESVi	Reduced EF (LVEF ≤ 40% and mixed <50% cohorts)	CMR	3	228	serious	serious	serious	serious	cannot be assessed	Very low	Direction inconsistent. Reduction in two trials, no change in the one trial for which this was the prespecified primary endpoint.
LVESV / LVESVi	Reduced EF (LVEF ≤40% and mixed <50% cohorts)	Echocardiography	4	466	serious	serious	serious	serious	cannot be assessed	Very low	Direction inconsistent.
LVEF	Reduced EF (LVEF ≤ 40% and mixed <50% cohorts)	CMR and echocardiography combined	8	640	serious	very serious	serious	not serious	cannot be assessed	Very low	Probably little or no average effect on ejection fraction, with one strongly discrepant trial.
GLS	Reduced EF (LVEF ≤ 40% and mixed <50% cohorts)	CMR feature tracking	2	127	serious	not serious	not serious	serious	cannot be assessed	Low	Probably little or no effect on CMR-derived longitudinal strain.
GLS	Reduced EF (LVEF ≤ 40% and mixed <50% cohorts)	Speckle-tracking echocardiography	4	446	very serious	serious	serious	serious	cannot be assessed	Very low	Longitudinal strain may improve modestly, but the effect size is strongly confounded with risk of bias and the single low-risk-of-bias estimate is the smallest.
LV mass / LVMi	Reduced EF (LVEF ≤ 40% and mixed <50% cohorts)	CMR	4	263	serious	serious	serious	serious	cannot be assessed	Very low	Direction inconsistent. Reduction in two of four CMR trials.
LAVi	Reduced EF (LVEF ≤ 40% and mixed <50% cohorts)	Echocardiography	4	446	very serious	serious	serious	serious	cannot be assessed	Very low	Left atrial volume index may fall, but no estimate is both precise and free of serious bias.
E/e-prime	Reduced EF (LVEF ≤ 40% and mixed <50% cohorts)	Echocardiography	2	128	very serious	not serious	serious	very serious	cannot be assessed	Very low	Very uncertain. E/e-prime may fall, but the evidence comes only from unblinded trials without between-group estimates.
PCWP	Reduced EF (LVEF ≤ 40%)	Invasive right heart catheterisation	1	66	very serious	not applicable	not serious	serious	cannot be assessed	Very low	Very uncertain. The prespecified haemodynamic primary endpoint was neutral; the reported PCWP reduction derives from a post hoc pooled analysis.
NT-proBNP	Reduced EF (LVEF ≤ 40% and mixed <50% cohorts)	Central laboratory assay	6	588	serious	serious	serious	not serious	cannot be assessed	Very low	Direction inconsistent. NT-proBNP fell in three of six reports, including the one trial at low risk of bias with a prespecified adjusted estimate, but the trial in which it was the registered primary endpoint was neutral.
LVEDV / LVEDVi	Preserved EF (LVEF ≥ 50%)	CMR and echocardiography	3	162	very serious	not serious	serious	serious	cannot be assessed	Very low	Probably little or no effect on left ventricular volumes in HFpEF, but the certainty is very low.
LVEF	Preserved EF (LVEF ≥ 50%)	CMR and echocardiography	4	306	very serious	not serious	serious	serious	cannot be assessed	Very low	Probably little or no effect.
GLS	Preserved EF (LVEF ≥ 50%)	CMR feature tracking and speckle tracking	2	94	very serious	not serious	serious	very serious	cannot be assessed	Very low	No evidence of improvement in longitudinal strain in HFpEF. The previously reported favourable trend rests on a direction error.
LV mass / LVMi	Preserved EF (LVEF ≥ 50%)	CMR and echocardiography	3	162	very serious	not serious	serious	serious	cannot be assessed	Very low	Probably little or no effect overall; an isolated subgroup finding in older patients.
LAVi	Preserved EF (LVEF ≥ 50%)	Echocardiography	2	138	very	serious	serious	serious	cannot be	Very low	Left atrial volume index may fall in HFpEF, but the

					serious				assessed		evidence rests on one open-label trial.
E/e-prime	Preserved EF (LVEF ≥ 50%)	Echocardiography	3	259	very serious	very serious	serious	serious	cannot be assessed	Very low	Very uncertain and conflicting. The one trial in which E/e-prime was a prespecified co-primary endpoint found no effect.
PCWP	Preserved EF (LVEF ≥ 50%)	Invasive exercise haemodynamics	1	38	not serious	not applicable	not serious	serious	cannot be assessed	Low	Dapagliflozin probably reduces PCWP at rest by about 3.5 mmHg and during exercise by about 5.7 mmHg in HFpEF. This is the most secure single finding in the review.
NT-proBNP	Preserved EF (LVEF ≥ 50%)	Central laboratory assay	4	325	very serious	serious	serious	serious	cannot be assessed	Very low	Direction inconsistent and very uncertain. NT-proBNP may fall, but the significant results are exploratory or post hoc, and the largest overall estimates are non-significant.

Across the 20 evidence profiles the revised certainty is Very low for 18 and Low for 2. No outcome retains a High certainty rating. The two Low ratings are CMR-derived GLS in reduced ejection fraction and PCWP in HFpEF (CAMEO-DAPA).

Table S3b. GRADE appendix: full written rationale for every domain judgement

LVEDV/LVEDVi — Reduced EF (LVEF ≤ 40% and mixed <50% cohorts) — CMR

Studies contributing (k = 4, n = 263): EMPA-TROPISM; SUGAR-DM-HF; REFORM; EMPA-VISION (HFrEF cohort)

Domain	Judgement	Rationale
Risk of bias	serious	One of the four CMR trials is at high risk of bias (EMPA-VISION HFrEF: exploratory outcome, per-protocol analysis) and the two positive trials have reporting limitations - EMPA-TROPISM publishes no adjusted between-group estimate or confidence interval at all, and SUGAR-DM-HF has 12% unevenly distributed attrition. Only SUGAR-DM-HF provides a prespecified, adjusted, confidence-interval-bearing estimate.
Inconsistency	serious	The four estimates are not compatible in magnitude or direction: EMPA-TROPISM -23.6 mL (derived) and SUGAR-DM-HF -8.2 mL/m2 (95% CI -13.7 to -2.6) versus REFORM +7.83 mL (95% CI -15.05 to 30.70) and EMPA-VISION -7.00 mL (95% CI -21.98 to 8.85). REFORM point estimate is in the opposite direction and its confidence interval barely overlaps that of EMPA-TROPISM. Reported as absolute volumes in two trials and indexed volumes in two others, which is itself a source of incompatibility.
Indirectness	serious	Two of the four cohorts are not restricted to LVEF ≤ 40%: EMPA-TROPISM enrolled LVEF < 50% and REFORM states no numerical entry threshold, so the population is a mixture of reduced and mildly reduced ejection fraction rather than HFrEF as defined by current guidelines. Follow-up ranges from 12 weeks to 12 months, and 12 weeks is short for volumetric remodelling.
Imprecision	serious	Total analysed n = 263 across four trials, well below any conventional threshold for an adequately powered continuous outcome. Individual confidence intervals are wide and, for REFORM and EMPA-VISION, include both clinically important benefit and clinically important harm.
Publication bias	cannot be assessed	With four or fewer small trials, neither a funnel plot nor a formal test for small-study effects can be interpreted, so publication bias cannot be assessed. It cannot be assumed absent: mechanistic imaging substudies with neutral results are known to be reported late or only within composite mechanistic papers.

Certainty of evidence: Very low. Summary: Direction inconsistent. Reduction of roughly 8 to 24 mL (or 8 mL/m2 indexed) in two trials, no change in two others.

LVEDV/LVEDVi — Reduced EF (LVEF ≤ 40% and mixed <50% cohorts) — Echocardiography

Studies contributing (k = 4, n = 466): Empire HF (echocardiographic substudy); DAPA-ECHO; Fu 2023; EFFORT

Domain	Judgement	Rationale
Risk of bias	serious	The largest contributor (Empire HF, n=190) is at high risk of bias because the echocardiographic outcomes were explicitly not prespecified and their significance is model-dependent. DAPA-ECHO reports within-group change scores only. EFFORT is at low risk of bias and neutral. Fu 2023 is at some concerns with apparently retrospective registration.
Inconsistency	serious	Two trials report reductions (Empire HF -5.5 mL/m2, 95% CI -10.6 to -0.4; Fu -6.0 mL, 95% CI -8.07 to -3.87) and two report no change (DAPA-ECHO -0.8 mL/m2, P=0.83; EFFORT non-significant). The two neutral trials include the only adequately powered, low-risk trial in the set.
Indirectness	serious	DAPA-ECHO (LVEF < 50%, 38% mildly reduced) and EFFORT (LVEF 35% to < 50%) are mixed reduced and mildly reduced ejection fraction cohorts, not HFrEF cohorts. EFFORT additionally required substantial functional mitral regurgitation, a population in which loading conditions and therefore volumes behave differently.
Imprecision	serious	Although the combined analysed n is 466, no single trial exceeds 190 and the two positive estimates come from a post hoc analysis and from a trial whose confidence intervals are implausibly narrow for its size.
Publication bias	cannot be assessed	With four or fewer small trials, neither a funnel plot nor a formal test for small-study effects can be interpreted, so publication bias cannot be assessed. It cannot be assumed absent: mechanistic imaging substudies with neutral results are known to be reported late or only within composite mechanistic papers.

Certainty of evidence: Very low. Summary: Direction inconsistent. Reduction of about 5 to 6 mL or mL/m2 in two trials, no change in two.

LVESV / LVESVi — Reduced EF (LVEF ≤ 40% and mixed <50% cohorts) — CMR

Studies contributing (k = 3, n = 228): EMPA-TROPISM; SUGAR-DM-HF; REFORM

Domain	Judgement	Rationale
Risk of bias	serious	As for LVEDV. EMPA-VISION did not report end-systolic volume at all, so only three CMR trials contribute. SUGAR-DM-HF is the only trial in which LVESVi was a prespecified co-primary endpoint.
Inconsistency	serious	EMPA-TROPISM -26.1 mL (derived) and SUGAR-DM-HF -6.0 mL/m2 (95% CI -10.8 to -1.2) versus REFORM +4.82 mL (95% CI -13.28 to 22.93), whose primary endpoint this was and which was frankly neutral.
Indirectness	serious	As for LVEDV: mixed ejection-fraction cohorts and absolute versus indexed reporting.
Imprecision	serious	Three trials, 228 participants analysed. REFORM confidence interval spans a 36 mL range.
Publication bias	cannot be assessed	With four or fewer small trials, neither a funnel plot nor a formal test for small-study effects can be interpreted, so publication bias cannot be assessed. It cannot be assumed absent: mechanistic imaging substudies with neutral results are known to be reported late or only within composite mechanistic papers.

Certainty of evidence: Very low. Summary: Direction inconsistent. Reduction in two trials, no change in the one trial for which this was the prespecified primary endpoint.

LVESV / LVESVi — Reduced EF (LVEF ≤ 40% and mixed <50% cohorts) — Echocardiography

Studies contributing (k = 4, n = 466): Empire HF (echocardiographic substudy); DAPA-ECHO; Fu 2023; EFFORT

Domain	Judgement	Rationale
Risk of bias	serious	As for echocardiographic LVEDV.
Inconsistency	serious	Empire HF -4.3 mL/m2 (95% CI -8.5 to -0.1, and -4.0, 95% CI -8.2 to 0.2 after further adjustment) and Fu -8.1 mL (95% CI -11.07 to -5.14) versus neutral results in DAPA-ECHO and EFFORT.
Indirectness	serious	As for echocardiographic LVEDV.
Imprecision	serious	The Empire HF estimate loses statistical significance under an alternative prespecified adjustment, which is the definition of a fragile result.
Publication bias	cannot be assessed	With four or fewer small trials, neither a funnel plot nor a formal test for small-study effects can be interpreted, so publication bias cannot be assessed. It cannot be assumed absent: mechanistic imaging substudies with neutral results are known to be reported late or only within composite mechanistic papers.

Certainty of evidence: Very low. Summary: Direction inconsistent.

LVEF — Reduced EF (LVEF ≤ 40% and mixed <50% cohorts) — CMR and echocardiography combined

Studies contributing (k = 9, n = 640): EMPA-TROPISM; SUGAR-DM-HF; REFORM; EMPA-VISION (HFrEF); Empire HF (echo); DAPA-ECHO; Reis 2022; Fu 2023; EFFORT

Domain	Judgement	Rationale
Risk of bias	serious	Three contributing reports are at high risk of bias (Empire HF echocardiographic substudy, EMPA-VISION HFrEF, Reis 2022) and none of the remaining reports is at low risk for this outcome.
Inconsistency	very serious	Seven of nine estimates are null and mutually consistent (SUGAR-DM-HF +0.3, 95% CI -1.7 to 2.3; REFORM +0.69, 95% CI -3.32 to 4.69; EMPA-VISION +1.87, 95% CI -1.60 to 5.40; Empire HF +1.2, 95% CI -1.2 to 3.6; DAPA-ECHO +1.2, P=0.38; Reis +1.7, P=0.512; EFFORT non-significant), whereas EMPA-TROPISM reports +6.1 percentage points and Fu +2.5 (95% CI 1.00 to 4.06). The EMPA-TROPISM estimate lies far outside the confidence intervals of every other trial and cannot be reconciled with them; this is genuine unexplained heterogeneity, not imprecision.
Indirectness	serious	Mixed reduced and mildly reduced ejection-fraction cohorts; CMR and echocardiographic ejection fraction have different normative ranges and reproducibility, and are combined here only because the estimates are null in both modalities.
Imprecision	not serious	The pooled information size is adequate to exclude a clinically important average effect: seven independent confidence intervals exclude an improvement of more than about 5 percentage points. Imprecision is therefore not the limiting factor for this outcome.
Publication bias	cannot be assessed	With four or fewer small trials, neither a funnel plot nor a formal test for small-study effects can be interpreted, so publication bias cannot be assessed. It cannot be assumed absent: mechanistic imaging substudies with neutral results are known to be reported late or only within composite mechanistic papers.

Certainty of evidence: Very low. Summary: Probably little or no average effect on ejection fraction, with one strongly discrepant trial.

GLS — Reduced EF (LVEF ≤ 40% and mixed <50% cohorts) — CMR feature tracking

Studies contributing (k = 2, n = 127): SUGAR-DM-HF; EMPA-VISION (HFrEF cohort)

Domain	Judgement	Rationale
Risk of bias	serious	EMPA-VISION HFrEF is at high risk of bias (exploratory, per-protocol, multiplicity). SUGAR-DM-HF is at some concerns and is the only trial in which strain was a prespecified co-primary endpoint.
Inconsistency	not serious	Both estimates are null and in the same direction: SUGAR-DM-HF +0.35 (95% CI -0.25 to 0.95) and EMPA-VISION +1.95 (95% CI -0.60 to 4.50). In this sign convention a positive value denotes strain becoming less negative, that is, no improvement. The confidence intervals overlap substantially.
Indirectness	not serious	Both are CMR feature-tracking measurements in reduced ejection-fraction populations; the assessment is internally coherent.
Imprecision	serious	Only 127 participants. The EMPA-VISION confidence interval is wide enough to include a clinically meaningful change in either direction.
Publication bias	cannot be assessed	Two trials only; assessment impossible.

Certainty of evidence: Low. Summary: Probably little or no effect on CMR-derived longitudinal strain.

GLS — Reduced EF (LVEF ≤ 40% and mixed <50% cohorts) — Speckle-tracking echocardiography

Studies contributing (k = 4, n = 446): Empire HF (echocardiographic substudy); DAPA-ECHO; Reis 2022; EFFORT

Domain	Judgement	Rationale
Risk of bias	very serious	Three of the four contributing reports are at high risk of bias for this outcome. DAPA-ECHO and Reis 2022 are open-label with NO STATEMENT that strain readers were blinded, and Reis 2022 is additionally unregistered with an implausibly deteriorating control arm. Empire HF strain is an unprespecified post hoc outcome. Only EFFORT, which used a blinded core laboratory, is at low risk of bias for strain.
Inconsistency	serious	EFFORT, the one low-risk trial, reports a modest -1.44 percentage points (95% CI -2.42 to -0.46). Empire HF reports no effect (+0.4, 95% CI -0.3 to 1.0). DAPA-ECHO and Reis 2022 report effects of -2.5 and -3.8 percentage points, two to three times larger than in the only blinded trial. The gradient in effect size tracks the gradient in risk of bias almost exactly.
Indirectness	serious	DAPA-ECHO and EFFORT are mixed reduced and mildly reduced ejection-fraction cohorts; EFFORT required significant functional mitral regurgitation. Two of the four trials provide no adjusted between-group

		estimate at all, only within-group change scores or interaction p values, so the quantity being synthesised is not the same across trials.
Imprecision	serious	Only EFFORT contributes an interpretable confidence interval; its lower bound (-0.46 percentage points) is of uncertain clinical importance.
Publication bias	cannot be assessed	With four or fewer small trials, neither a funnel plot nor a formal test for small-study effects can be interpreted, so publication bias cannot be assessed. It cannot be assumed absent: mechanistic imaging substudies with neutral results are known to be reported late or only within composite mechanistic papers.

Certainty of evidence: Very low. Summary: Longitudinal strain may improve modestly, but the effect size is strongly confounded with risk of bias and the single low-risk-of-bias estimate is the smallest.

LV mass / LVMi — Reduced EF (LVEF ≤ 40% and mixed <50% cohorts) — CMR

Studies contributing (k = 4, n = 263): EMPA-TROPISM; SUGAR-DM-HF; REFORM; EMPA-VISION (HFrEF cohort)

Domain	Judgement	Rationale
Risk of bias	serious	EMPA-VISION HFrEF, which contributes one of the two positive estimates, is at high risk of bias (exploratory outcome, per-protocol set, large multiplicity). EMPA-TROPISM publishes no adjusted estimate.
Inconsistency	serious	EMPA-TROPISM -21.9 g (derived) and EMPA-VISION -4.46 g/m2 (95% CI -8.42 to -0.50) versus SUGAR-DM-HF -1.9 g/m2 (95% CI -4.7 to 0.8) and REFORM +2.5 g/m2 (95% CI -3.95 to 8.95). Two trials show reduction, two show none, and the point estimates differ several-fold.
Indirectness	serious	Mixed cohorts; mass reported as absolute grams in two trials and indexed in two others.
Imprecision	serious	Only 263 participants across four trials; the EMPA-VISION confidence interval nearly touches zero.
Publication bias	cannot be assessed	With four or fewer small trials, neither a funnel plot nor a formal test for small-study effects can be interpreted, so publication bias cannot be assessed. It cannot be assumed absent: mechanistic imaging substudies with neutral results are known to be reported late or only within composite mechanistic papers.

Certainty of evidence: Very low. Summary: Direction inconsistent. Reduction in two of four CMR trials.

LAVi — Reduced EF (LVEF ≤ 40% and mixed <50% cohorts) — Echocardiography

Studies contributing (k = 4, n = 446): Empire HF (echocardiographic substudy); DAPA-ECHO; Reis 2022; EFFORT

Domain	Judgement	Rationale
Risk of bias	very serious	The Empire HF estimate is post hoc and loses significance under alternative prespecified adjustment (-2.5, 95% CI -4.8 to -0.1 becoming -2.2, 95% CI -4.6 to 0.2). Reis 2022 is at high risk of bias throughout. The EFFORT estimate carries an unresolved source-level reporting error: the published 95% CI (-12.16 to 0.15) crosses zero although P is given as 0.005.
Inconsistency	serious	Estimates range from -2.5 mL/m2 (Empire HF) through no change (DAPA-ECHO -1.0, P=0.49) to -6.0 (EFFORT) and -13.1 (Reis 2022, derived). The Reis 2022 estimate is an order of magnitude larger than the others and is produced almost entirely by a rise in its control arm.
Indirectness	serious	Mixed ejection-fraction cohorts; EFFORT population defined by functional mitral regurgitation, in which atrial volume is mechanically determined by the regurgitant lesion rather than by ventricular remodelling.
Imprecision	serious	Every interpretable confidence interval either touches or crosses zero.
Publication bias	cannot be assessed	With four or fewer small trials, neither a funnel plot nor a formal test for small-study effects can be interpreted, so publication bias cannot be assessed. It cannot be assumed absent: mechanistic imaging substudies with neutral results are known to be reported late or only within composite mechanistic papers.

Certainty of evidence: Very low. Summary: Left atrial volume index may fall, but no estimate is both precise and free of serious bias.

E/e-prime — Reduced EF (LVEF ≤ 40% and mixed <50% cohorts) — Echocardiography

Studies contributing (k = 2, n = 128): DAPA-ECHO; Reis 2022

Domain	Judgement	Rationale
Risk of bias	very serious	Both contributing trials are open-label, single-centre, without placebo, and neither states that echocardiographic readers were blinded. Reis 2022 is unregistered. E/e-prime is strongly load-dependent and therefore highly susceptible to differential diuretic management in an unblinded trial. Neither trial reports an adjusted between-group estimate.
Inconsistency	not serious	Both point in the same direction and are of broadly similar size (DAPA-ECHO -1.7 within group; Reis 2022 -2.9 derived).
Indirectness	serious	Both are mixed reduced and mildly reduced ejection-fraction cohorts; in neither is E/e-prime a prespecified endpoint.
Imprecision	very serious	Only 128 participants in two small single-centre trials, with no confidence interval for the between-group contrast in either.
Publication bias	cannot be assessed	Two trials only; assessment impossible.

Certainty of evidence: Very low. Summary: Very uncertain. E/e-prime may fall, but the evidence comes only from unblinded trials without between-group estimates.

PCWP — Reduced EF (LVEF ≤ 40%) — Invasive right heart catheterisation

Studies contributing (k = 1, n = 66): Empire HF (haemodynamic substudy)

Domain	Judgement	Rationale
Risk of bias	very serious	The registered primary endpoint (peak-exercise PCWP/cardiac index) was NEUTRAL (0.13, 95% CI -1.60 to 1.34, P=0.86). Resting PCWP showed no significant between-group effect (-1.47, 95% CI -3.86 to 0.82, P=0.23). The only significant result, PCWP pooled across all exercise loads (-2.40, 95% CI -3.96 to -0.84), comes from an analysis the trialists themselves describe as post hoc, and the trial reports significant baseline imbalance between arms in exercise PCWP.
Inconsistency	not applicable	A single trial; inconsistency cannot be assessed. This alone precludes a rating above Low.
Indirectness	not serious	The population (LVEF ≤ 40%, stable ambulatory HFrEF) and the measurement (invasive high-fidelity micromanometry) match the review question directly.
Imprecision	serious	A single trial with 66 evaluable participants and no replication.
Publication bias	cannot be assessed	A single trial; assessment impossible.

Certainty of evidence: Very low (previously Moderate (pooled with CAMEO-DAPA)). Summary: Very uncertain. The prespecified haemodynamic primary endpoint was neutral; the reported PCWP reduction derives from a post hoc pooled analysis.

NT-proBNP — Reduced EF (LVEF ≤ 40% and mixed <50% cohorts) — Central laboratory assay

Studies contributing (k = 6, n = 588): EMPA-TROPISM; SUGAR-DM-HF; Empire HF (parent trial); DAPA-ECHO; Reis 2022; EFFORT

Domain	Judgement	Rationale
Risk of bias	serious	Only SUGAR-DM-HF (low risk of bias for this outcome, adjusted between-group ratio -28%, 95% CI -47 to -2) and EFFORT (low risk, non-significant) provide reliable estimates. Reis 2022 is at high risk of bias and its effect is driven by a rise in the control arm. EMPA-TROPISM reports percentage change per arm with no adjusted estimate.
Inconsistency	serious	Three trials report reductions and three do not. Critically, the two trials at LOW risk of bias disagree with each other: SUGAR-DM-HF is positive and EFFORT is null, and the Empire HF parent trial, for which NT-proBNP was the registered primary endpoint, was also null.
Indirectness	serious	Mixed ejection-fraction cohorts; NT-proBNP was a prespecified primary endpoint in only one trial and an exploratory outcome elsewhere. Reported variously as absolute change, percentage change and log-ratio, which are not interchangeable.
Imprecision	not serious	588 participants with several interpretable confidence intervals; imprecision is not the limiting factor.
Publication bias	cannot be assessed	With four or fewer small trials, neither a funnel plot nor a formal test for small-study effects can be interpreted, so publication bias cannot be assessed. It cannot be assumed absent: mechanistic imaging substudies with neutral results are known to be reported late or only within composite mechanistic papers.

Certainty of evidence: Very low. Summary: Direction inconsistent. NT-proBNP fell in three of six reports, including the one trial at low risk of bias with a prespecified adjusted estimate, but the trial in which it was the registered primary endpoint was neutral.

LVEDV / LVEDVi — Preserved EF (LVEF ≥ 50%) — CMR and echocardiography

Studies contributing (k = 3, n = 162): EMPA-VISION (HFpEF cohort); Ovchinnikov 2025; EXCEED

Domain	Judgement	Rationale
Risk of bias	very serious	EMPA-VISION HFpEF is at high risk of bias in three domains: only 24 of 36 randomised participants were analysed, on a per-protocol basis, with marked baseline imbalance in LVEDV between the analysed arms (174 versus 122 mL). Ovchinnikov 2025 is open-label without stated reader blinding. EXCEED used site-based rather than core-laboratory echocardiography.
Inconsistency	not serious	All three estimates are null and mutually compatible: EMPA-VISION -2.74 mL (95% CI -20.44 to 14.95), Ovchinnikov -0.2 mL (P=0.93), EXCEED -4.9 mL (95% CI -14.9 to 5.1).
Indirectness	serious	CMR and echocardiographic volumes are combined out of necessity; all three cohorts are diabetic or largely diabetic, which limits applicability to non-diabetic HFpEF.
Imprecision	serious	Only 162 participants. The EMPA-VISION confidence interval spans 35 mL.
Publication bias	cannot be assessed	Three small trials; assessment impossible.

Certainty of evidence: Very low. Summary: Probably little or no effect on left ventricular volumes in HFpEF, but the certainty is very low.

LVEF — Preserved EF (LVEF ≥ 50%) — CMR and echocardiography

Studies contributing (k = 3, n = 162): EMPA-VISION (HFpEF cohort); Ovchinnikov 2025; EXCEED

Domain	Judgement	Rationale

Risk of bias	very serious	The same three trials and the same three defects apply as for LVEDV in this phenotype: the EMPA-VISION HFpEF cohort analysed only 24 of 36 randomised participants on a per-protocol basis with marked baseline imbalance in the very variable being measured (baseline LVEF 52.6% versus 59.1%); Ovchinnikov 2025 is open-label without any statement that readers were blinded; and EXCEED measured ejection fraction at each participating site rather than in a core laboratory, which its own authors identify as a limitation.
Inconsistency	not serious	All null and compatible: EMPA-VISION +1.47 (95% CI -2.89 to 5.83), Ovchinnikov -0.1 (P=0.58), EXCEED +1.3 (95% CI -1.4 to 4.0).
Indirectness	serious	As for LVEDV in HFpEF; a ceiling effect is expected when baseline ejection fraction is normal.
Imprecision	serious	Only 162 participants across three trials, the largest contributing 68. The confidence intervals span roughly 6 to 9 percentage points, wide enough to include a clinically important change in either direction even though every point estimate lies close to zero.
Publication bias	cannot be assessed	Three trials contribute, none exceeding 68 participants. A funnel plot cannot be interpreted with fewer than about ten comparisons and no formal test for small-study effects is applicable. Publication bias is therefore impossible to assess rather than demonstrably absent.

Certainty of evidence: Very low. Summary: Probably little or no effect.

GLS — Preserved EF (LVEF ≥ 50%) — CMR feature tracking and speckle tracking

Studies contributing (k = 2, n = 94): EMPA-VISION (HFpEF cohort); Ovchinnikov 2025

Domain	Judgement	Rationale
Risk of bias	very serious	EMPA-VISION HFpEF is at high risk of bias in three domains AND the source publication misstates the direction of its own strain result: longitudinal strain moved from -14.16% to -12.31% with empagliflozin and from -13.53% to -13.84% with placebo, so the adjusted difference of +2.18% denotes strain becoming LESS negative, that is numerically worse, with treatment. It was described in the source, and repeated in the submitted manuscript, as a trend toward improvement. Ovchinnikov 2025 is open-label without stated reader blinding.
Inconsistency	not serious	Both estimates are non-significant. Correctly oriented, both are compatible with no benefit: EMPA-VISION +2.18 (95% CI -0.28 to 4.64), which is directionally unfavourable, and Ovchinnikov -0.5 (95% CI -1.4 to 0.3), which is directionally favourable but null.
Indirectness	serious	Two different strain techniques in two small diabetic or largely diabetic cohorts.
Imprecision	very serious	Only 94 participants, of whom 24 in the EMPA-VISION cohort. No estimate excludes clinically important change in either direction.
Publication bias	cannot be assessed	Only two trials contribute, so neither a funnel plot nor a test for small-study effects can be interpreted. Publication bias is impossible to assess and cannot be assumed absent, particularly for a strain outcome that was exploratory in one trial and secondary in the other.

Certainty of evidence: Very low. Summary: No evidence of improvement in longitudinal strain in HFpEF. The previously reported favourable trend rests on a direction error.

LV mass / LVMi — Preserved EF (LVEF ≥ 50%) — CMR and echocardiography

Studies contributing (k = 3, n = 162): EMPA-VISION (HFpEF cohort); Ovchinnikov 2025; EXCEED

Domain	Judgement	Rationale
Risk of bias	very serious	The same three defects apply as for the other structural outcomes in this phenotype: per-protocol analysis of 24 of 36 randomised participants with baseline imbalance in EMPA-VISION; absence of any statement of reader blinding in the open-label Ovchinnikov trial; and site-based rather than core-laboratory measurement in EXCEED. Left ventricular mass is additionally the structural outcome most sensitive to measurement technique and to loading conditions at the time of imaging.
Inconsistency	not serious	All three overall estimates are null and compatible: EMPA-VISION -1.18 g/m2 (95% CI -7.06 to 4.71), Ovchinnikov -2.8 (95% CI -7.9 to 2.3), EXCEED non-significant overall.
Indirectness	serious	The only positive result is a subgroup of 38 participants aged 70 years or over within EXCEED (-19.9 g/m2, 95% CI -38.0 to -1.75), which is not the review population and is a post hoc subgroup in a trial with two neutral co-primary endpoints.
Imprecision	serious	Only 162 participants in the overall analyses.
Publication bias	cannot be assessed	Three trials contribute, none exceeding 68 participants, so no funnel plot or small-study test is interpretable. The only positive result is a post hoc subgroup analysis, which is itself a recognised marker of selective reporting but cannot be assessed formally with so few comparisons.

Certainty of evidence: Very low. Summary: Probably little or no effect overall; an isolated subgroup finding in older patients.

LAVi — Preserved EF (LVEF ≥ 50%) — Echocardiography

Studies contributing (k = 2, n = 138): Ovchinnikov 2025; EXCEED

Domain	Judgement	Rationale
Risk of bias	very serious	Ovchinnikov 2025, the only trial with a significant result (-3.4 mL/m2, 95% CI -5.1 to -1.6), is open-label without stated reader blinding, and left atrial volume is load-dependent and therefore susceptible to unblinded diuretic adjustment. EXCEED reports absolute left atrial volume, NOT indexed to body surface area, so the two are not the same measure.
Inconsistency	serious	Ovchinnikov reports a clear reduction; EXCEED reports a non-significant change in a differently defined variable (-7.1 mL, 95% CI -14.4 to 0.2, P=0.06). Pooling an indexed and a non-indexed measure would be

		inappropriate.
Indirectness	serious	Both cohorts are entirely or almost entirely diabetic; EXCEED excluded atrial fibrillation and valvular disease, which materially restricts the HFpEF population.
Imprecision	serious	Only 138 participants; a single significant estimate.
Publication bias	cannot be assessed	Only two trials contribute and they report different measures, indexed and non-indexed atrial volume, so neither a funnel plot nor a test for small-study effects is applicable. Publication bias is impossible to assess.

Certainty of evidence: Very low. Summary: Left atrial volume index may fall in HFpEF, but the evidence rests on one open-label trial.

E/e-prime — Preserved EF (LVEF ≥ 50%) — Echocardiography

Studies contributing (k = 2, n = 138): Ovchinnikov 2025; EXCEED

Domain	Judgement	Rationale
Risk of bias	very serious	The two trials disagree, and the disagreement aligns with study design. EXCEED, in which E/e-prime was a REGISTERED CO-PRIMARY endpoint analysed with a multivariable-adjusted between-group estimate, was frankly neutral (-0.04, 95% CI -1.3 to 1.2, P=0.95). Ovchinnikov 2025, open-label and without stated reader blinding, reports -1.8 (95% CI -2.6 to -1.1, P<0.001) for a secondary endpoint.
Inconsistency	very serious	The two estimates are frankly incompatible: the Ovchinnikov confidence interval (-2.6 to -1.1) lies entirely outside the EXCEED confidence interval (-1.3 to 1.2). One trial finds a large effect and the other excludes it.
Indirectness	serious	Both cohorts are diabetic; EXCEED excluded atrial fibrillation and used site-based echocardiography, while Ovchinnikov required exercise-induced elevation of E/e-prime for entry, which enriches for reversibility.
Imprecision	serious	Only 138 participants in total. More importantly, the two confidence intervals do not overlap, so the available data do not identify a single plausible range for the effect; the uncertainty is not merely wide but internally contradictory.
Publication bias	cannot be assessed	Only two trials contribute, so publication bias cannot be assessed by any formal method. It is worth noting that the trial reporting no effect prespecified this outcome as co-primary while the trial reporting a large effect treated it as secondary, which is the direction of asymmetry that selective reporting would produce.

Certainty of evidence: Very low. Summary: Highly uncertain and frankly conflicting. The one trial in which E/e-prime was a prespecified co-primary endpoint found no effect.

PCWP — Preserved EF (LVEF ≥ 50%) — Invasive exercise haemodynamics

Studies contributing (k = 1, n = 38): CAMEO-DAPA

Domain	Judgement	Rationale
Risk of bias	not serious	CAMEO-DAPA is at low risk of bias in Domains 1, 2, 4 and 5: double-blind, placebo-controlled, prespecified composite rest-and-exercise PCWP primary endpoint that was MET, measured by invasive high-fidelity micromanometry. The only reservation, recorded as some concerns in Domain 3, is that per-outcome denominators fall to 14 of 20 randomised placebo participants for some pressures. This does not reach the threshold for downgrading certainty.
Inconsistency	not applicable	A single trial; inconsistency cannot be assessed. This limitation alone prevents a rating above Low, irrespective of the trial quality.
Indirectness	not serious	The population (LVEF ≥ 50% with invasively confirmed exercise-induced elevation of PCWP), the intervention, the comparator and the outcome all match the review question directly, and the measurement is the reference standard.
Imprecision	serious	A single trial with 38 treated participants. The confidence intervals are wide relative to the point estimates (rest -3.5 mmHg, 95% CI -6.6 to -0.4; peak exercise -5.7 mmHg, 95% CI -10.8 to -0.7) and their lower bounds approach zero, so although the effect is statistically significant its magnitude is poorly determined.
Publication bias	cannot be assessed	A single trial; assessment impossible. Note that CAMEO-DAPA is the only invasive HFpEF trial identified, so a neutral counterpart, if one existed, might not be visible.

Certainty of evidence: Low. Summary: Dapagliflozin probably reduces PCWP at rest by about 3.5 mmHg and during exercise by about 5.7 mmHg in HFpEF. This is the most secure single finding in the review.

NT-proBNP — Preserved EF (LVEF ≥ 50%) — Central laboratory assay

Studies contributing (k = 4, n = 325): CAMEO-DAPA; Ovchinnikov 2025; EXCEED; CANDLE (LVEF ≥ 50% subgroup)

Domain	Judgement	Rationale
Risk of bias	very serious	The CAMEO-DAPA result is an explicitly EXPLORATORY outcome analysed as a median change with a rank-sum test, and the between-group difference reflects mainly a RISE in the placebo arm (+22 pg/mL) rather than a fall with dapagliflozin (-7 pg/mL). The CANDLE subgroup is post hoc in a trial that did not meet its prespecified non-inferiority primary endpoint, and its subgroup boundary (LVEF 50%) differs from the randomisation stratification boundary (40%). Ovchinnikov 2025 is open-label. EXCEED overall result was neutral and only a 16-patient subgroup was significant.
Inconsistency	serious	Two reports are significant and two are not, and the significant ones are the two at higher risk of selective-reporting bias. Magnitudes are not comparable: a median change of -7 pg/mL (CAMEO-DAPA) against -118.9 pg/mL (EXCEED, non-significant) and -58.3 pg/mL (CANDLE subgroup, non-significant).
Indirectness	serious	Three of the four cohorts are entirely or almost entirely diabetic, and one contributes only a post hoc subgroup rather than a randomised comparison in the review population.
Imprecision	serious	Although 325 participants contribute, no single trial exceeds 149 and the HFpEF-specific denominators are much smaller; every confidence interval that is reported includes no effect except in small subgroups.

Publication bias	cannot be assessed	With four or fewer small trials, neither a funnel plot nor a formal test for small-study effects can be interpreted, so publication bias cannot be assessed. It cannot be assumed absent: mechanistic imaging substudies with neutral results are known to be reported late or only within composite mechanistic papers.

Certainty of evidence: Very low. Summary: Direction inconsistent and very uncertain. NT-proBNP may fall, but the significant results are exploratory or post hoc, and the largest overall estimates are non-significant.

Table S4. Discrepancy log — all independent reviewer disagreements before consensus

#	Title	First author	Year	DOI	PMID	Screening stage	Reviewer 1 decision	Reviewer 2 decision	Eligibility criterion involved	Reason for the disagreement	Resolution method	Third reviewer involved	Final consensus decision
1	Effects of canagliflozin in patients with type 2 diabetes and chronic heart failure: a randomized trial (CANDLE)	Tanaka	2020	10.1002/ehf2.12707	32349193	Title/abstract	Include	Exclude	Comparator (PICO element C)	Reviewer 2 considered that an active comparator (glimepiride) did not satisfy the comparator criterion; reviewer 1 considered that the protocol admitted an active control, including another glucose-lowering agent.	Discussion, then third reviewer	Yes (M.A.)	Include — retained for full-text assessment
2	Left Ventricular Systolic Function After 3 Months of SGLT2 Inhibitor Therapy in Heart Failure Patients with Reduced Ejection Fraction	Mustapic	2023	10.1007/s12265-023-10389-3	37155137	Title/abstract	Include	Exclude	Study design (RCT-only criterion)	The abstract describes prospective inclusion of 59 outpatients allocated 41 to SGLT2 inhibitor and 18 to optimal medical therapy without stating that allocation was random. Reviewer 2 judged the design ineligible on that basis; reviewer 1 judged the design unclear and favoured full-text assessment.	Third reviewer	Yes (M.A.)	Include at title/abstract — retained for full-text assessment
3	Acute effects of empagliflozin on left atrial and ventricular filling parameters using echocardiography — a subanalysis of the EMPAG-HF trial	Bogoviku	2025	10.1093/ehjcvp/pvaf003	40037298	Title/abstract	Exclude	Include	Follow-up duration and population acuity	Reviewer 2 considered the echocardiographic remodelling endpoints relevant; reviewer 1 noted that treatment lasted 5 days in acute decompensated heart failure, failing both the 3-month minimum follow-up and the requirement for stable chronic heart failure.	Discussion	No	Exclude
4	Dapagliflozin reduces systemic inflammation in patients with type 2 diabetes without known heart failure	Wang	2024	10.1186/s12933-024-02294-z	38849829	Full text	Include	Exclude	Population (PICO element P)	Reviewer 1 initially retained the report because cardiac magnetic resonance strain, extracellular volume and T2 mapping were measured; reviewer 2 identified that the enrolled population had type 2 diabetes WITHOUT known heart failure and therefore failed the population criterion.	Discussion	No	Exclude
5	Effect of dapagliflozin on ventricular arrhythmias, resuscitated cardiac arrest, or sudden death in DAPA-HF	Curtain	2021	10.1093/eurheartj/ehab560	34448003	Full text	Exclude	Include	Outcome (PICO element O)	Reviewer 2 retained the report as a randomised analysis within a heart failure population; reviewer 1 noted that it is a post hoc analysis of arrhythmic events with no imaging, strain or haemodynamic remodelling endpoint.	Third reviewer	Yes (M.A.)	Exclude

Table S5. PRISMA S Checklist

Section/topic	#	Checklist item	Location(s) Reported
**INFORMATION SOURCES AND METHODS**			
Database name	1	Name each individual database searched, stating the platform for each.	Manuscript, Section 2.2 (Search Strategy); Supplementary Material, Table S6a (A1-A3: database and platform).
Multi-database searching	2	If databases were searched simultaneously on a single platform, state the name of the platform, listing all of the databases searched.	Not applicable. PubMed/MEDLINE, Embase and Scopus were searched separately on their

			respective platforms; no simultaneous multi-database platform search is described.
Study registries	3	List any study registries searched.	Not reported. PROSPERO is reported as the review registration (Methods), but no study registry has been searched.
Online resources and browsing	4	Describe any online or print source purposefully searched or browsed (e.g., tables of contents, print conference proceedings, web sites), and how this was done.	Not appliable
Citation searching	5	Indicate whether cited references or citing references were examined, and describe any methods used for locating cited/citing references (e.g., browsing reference lists, using a citation index, setting up email alerts for references citing included studies).	Manuscript, Section 2.2; Supplementary Material, Table S6b (Citation searching log). Reference lists of included articles and relevant systematic reviews were screened;
Contacts	6	Indicate whether additional studies or data were sought by contacting authors, experts, manufacturers, or others.	Manuscript, Section 4.8/Strengths and Limitations: missing or individual-participant data could not be obtained from original study authors. No search-specific author/expert/manufacture r contact method has been used.
Other methods	7	Describe any additional information sources or search methods used.	Supplementary Material, Table S6b (reference-list searching) and Table S6c (database retrieval validation). No other information source/search method is reported.
**SEARCH STRATEGIES**			
Full search strategies	8	Include the search strategies for each database and information source, copied and pasted exactly as run.	Supplementary Material, Table S6a (A1 PubMed/MEDLINE, A2 Embase, A3 Scopus): complete line-by-line strategies as documented for each database.

Limits and restrictions	9	Specify that no limits were used, or describe any limits or restrictions applied to a search (e.g., date or time period, language, study design) and provide justification for their use.	Manuscript, Sections 2.1-2.2; Supplementary Material, Table S6a. Restrictions: publication period 1 Feb 2016-31 Mar 2026; English language; randomized-trial filter; non-human records excluded. Eligibility rationale is described in Section 2.1.
Search filters	10	Indicate whether published search filters were used (as originally designed or modified), and if so, cite the filter(s) used.	Manuscript, Section 2.2; Supplementary Material, Table S6a. Cochrane Highly Sensitive Search Strategy (sensitivity-maximising version) is stated for PubMed and a Cochrane Handbook Embase RCT filter for Embase.
Prior work	11	Indicate when search strategies from other literature reviews were adapted or reused for a substantive part or all of the search, citing the previous review(s).	Not applicable / not reported. The strategy is described as constructed around three concepts and adapted across databases; no previous review search strategy has been reused.
Updates	12	Report the methods used to update the search(es) (e.g., rerunning searches, email alerts).	The manuscript reports a reconstructed search covering the prespecified period through 31 March 2026.
Dates of searches	13	For each search strategy, provide the date when the last search occurred.	Supplementary Material, Table S6a: PubMed/MEDLINE, Embase and Scopus search dates. Current file reports 31 July 2026 for Embase, Scopus and PubMed
**PEER REVIEW**			
Peer review	14	Describe any search peer review process.	Not reported. No formal peer review of the search strategy (e.g., PRESS or information-specialist review) was executed.
**MANAGING RECORDS**			

Total Records	15	Document the total number of records identified from each database and other information sources.	Manuscript, Section 3.1 (PRISMA flow); Supplementary Material, Table S6a (database yields)
Deduplication	16	Describe the processes and any software used to deduplicate records from multiple database searches and other information sources.	Manuscript, Section 3.1 reports 2,878 duplicates removed. Supplementary Material. The deduplication software used is Rayyan.

PRISMA-S: An Extension to the PRISMA Statement for Reporting Literature Searches in Systematic Reviews

Rethlefsen ML, Kirtley S, Waffenschmidt S, Ayala AP, Moher D, Page MJ, Koffel JB, PRISMA-S Group.

Last updated February 27, 2020.

Table S6a. Search appendix — complete line-by-line search strategies

The search was reconstructed rather than updated: the objective was to reproduce the original search over the reported period using the reported eligibility criteria. The verbatim PubMed string published in the submitted manuscript retrieves 425 records (419 English) over 1 February 2016 to 31 March 2026 and cannot account for the reported yield of 1749 PubMed records. The strategy below preserves the original three-concept structure while adding controlled vocabulary, truncation and an appropriate sensitivity-maximising randomised controlled trial filter.

A1. PubMed / MEDLINE

Field	Detail
Database and platform	PubMed / MEDLINE, National Center for Biotechnology Information web interface (https://pubmed.ncbi.nlm.nih.gov), searched programmatically through NCBI E-utilities
Date of last search	31 July 2026
Publication date limits	1 February 2016 to 31 March 2026 (datetype = publication date)
Language restriction	english[la]
Controlled vocabulary	MeSH: Sodium-Glucose Transporter 2 Inhibitors; Heart Failure; Ventricular Dysfunction, Left; Cardiomyopathies[noexp]; Ventricular Remodeling; Atrial Remodeling; Echocardiography; Magnetic Resonance Imaging, Cine; Stroke Volume; Diastole; Ventricular Function, Left; Natriuretic Peptide, Brain; Peptide Fragments; Cardiac Catheterization; Pulmonary Wedge Pressure; Hypertrophy, Left Ventricular; Heart Ventricles. Supplementary Concept records [nm] for each agent.
Free-text fields	Title/abstract [tiab] with truncation (*)
Boolean operators	OR within each concept; AND across the three concepts; NOT for the non-human limit
RCT filter	Cochrane Highly Sensitive Search Strategy, sensitivity-maximising version (2008 revision), PubMed format, combined with NOT (animals[mh] NOT humans[mh])
Total records retrieved	1236 before the language limit; 1203 after english[la]

Line	Search string	Records retrieved
1	"Sodium-Glucose Transporter 2 Inhibitors"[Mesh]	8939
2	("sodium glucose cotransporter 2 inhibitor*"[tiab] OR "sodium-glucose cotransporter 2 inhibitor*"[tiab] OR "sodium glucose co-transporter 2 inhibitor*"[tiab] OR "sodium-glucose co-transporter-2 inhibitor*"[tiab] OR "SGLT2 inhibitor*"[tiab] OR "SGLT-2 inhibitor*"[tiab] OR SGLT2i[tiab] OR "SGLT2-i"[tiab] OR gliflozin*[tiab])	15782
3	(empagliflozin[tiab] OR empagliflozin[nm] OR dapagliflozin[tiab] OR dapagliflozin[nm] OR canagliflozin[tiab] OR canagliflozin[nm] OR ertugliflozin[tiab] OR ertugliflozin[nm] OR ipragliflozin[tiab] OR ipragliflozin[nm] OR luseogliflozin[tiab] OR luseogliflozin[nm] OR tofogliflozin[tiab] OR tofogliflozin[nm] OR sotagliflozin[tiab] OR sotagliflozin[nm] OR bexagliflozin[tiab] OR bexagliflozin[nm] OR remogliflozin[tiab] OR henagliflozin[tiab])	8916
4	#1 OR #2 OR #3 — SGLT2 inhibitor concept	19261
5	("Heart Failure"[Mesh] OR "Ventricular Dysfunction, Left"[Mesh] OR "Cardiomyopathies"[Mesh:NoExp])	86231
6	("heart failure"[tiab] OR "cardiac failure"[tiab] OR "cardiac insufficiency"[tiab] OR HFrEF[tiab] OR HFmrEF[tiab] OR HFpEF[tiab] OR "reduced ejection fraction"[tiab] OR "preserved ejection fraction"[tiab] OR "mildly reduced	146873

	ejection fraction"[tiab] OR "mid-range ejection fraction"[tiab] OR "midrange ejection fraction"[tiab] OR "left ventricular dysfunction"[tiab] OR "cardiac decompensation"[tiab])	
7	#5 OR #6 — heart failure concept	169397
8	("Ventricular Remodeling"[Mesh] OR "Atrial Remodeling"[Mesh] OR "Echocardiography"[Mesh] OR "Magnetic Resonance Imaging, Cine"[Mesh] OR "Stroke Volume"[Mesh] OR "Diastole"[Mesh] OR "Ventricular Function, Left"[Mesh] OR "Natriuretic Peptide, Brain"[Mesh] OR "Peptide Fragments"[Mesh] OR "Cardiac Catheterization"[Mesh] OR "Pulmonary Wedge Pressure"[Mesh] OR "Hypertrophy, Left Ventricular"[Mesh] OR "Heart Ventricles"[Mesh])	134494
9	("cardiac remodeling"[tiab] OR "cardiac remodelling"[tiab] OR "ventricular remodeling"[tiab] OR "ventricular remodelling"[tiab] OR "reverse remodeling"[tiab] OR "reverse remodelling"[tiab] OR "atrial remodeling"[tiab] OR "atrial remodelling"[tiab] OR "global longitudinal strain"[tiab] OR GLS[tiab] OR "longitudinal strain"[tiab] OR "myocardial strain"[tiab] OR "speckle tracking"[tiab] OR "speckle-tracking"[tiab] OR "myocardial work"[tiab] OR "diastolic function"[tiab] OR "diastolic dysfunction"[tiab] OR "E/e prime"[tiab] OR "E/e'"[tiab] OR "left atrial volume"[tiab] OR LAVI[tiab] OR "left atrial strain"[tiab] OR "pulmonary capillary wedge pressure"[tiab] OR PCWP[tiab] OR "wedge pressure"[tiab] OR "filling pressure*"[tiab] OR "end-diastolic volume"[tiab] OR "end diastolic volume"[tiab] OR "end-systolic volume"[tiab] OR "end systolic volume"[tiab] OR LVEDV[tiab] OR LVESV[tiab] OR LVEDVi[tiab] OR LVESVi[tiab] OR "ejection fraction"[tiab] OR LVEF[tiab] OR "left ventricular mass"[tiab] OR LVMI[tiab] OR "LV mass"[tiab] OR "cardiac magnetic resonance"[tiab] OR "cardiovascular magnetic resonance"[tiab] OR echocardiograph*[tiab] OR "NT-proBNP"[tiab] OR "N-terminal pro-B-type natriuretic peptide"[tiab] OR "natriuretic peptide"[tiab] OR "right heart catheterization"[tiab] OR "cardiac catheterisation"[tiab] OR hemodynamic*[tiab] OR haemodynamic*[tiab])	227829
10	#8 OR #9 — remodelling / imaging / haemodynamic outcome concept	287884
11	#4 AND #7 AND #10	3011
12	("randomized controlled trial"[pt] OR "controlled clinical trial"[pt])	246111
13	(randomized[tiab] OR randomised[tiab] OR placebo[tiab] OR randomly[tiab] OR trial[ti] OR groups[tiab])	1997424
14	"clinical trials as topic"[MeSH Terms:noexp]	30053
15	#12 OR #13 OR #14 — Cochrane HSSS, sensitivity-maximising version	2035258
16	(animals[mh] NOT humans[mh])	1191326
17	#15 NOT #16	1859365
18	#11 AND #17	1,327
19	#18 AND english[la] — FINAL SET carried into screening	1285

Reproduction of the originally published string, for comparison: ("sodium-glucose cotransporter 2 inhibitors" OR "SGLT2 inhibitors" OR SGLT2i OR empagliflozin OR dapagliflozin OR canagliflozin OR ertugliflozin OR ipragliflozin) AND ("heart failure" OR HFrEF OR HFpEF OR "reduced ejection fraction" OR "preserved ejection fraction") AND ("cardiac remodeling" OR "ventricular remodeling" OR "reverse remodeling" OR "global longitudinal strain" OR GLS OR "diastolic function" OR "E/e'" OR "left atrial volume" OR LAVI OR "pulmonary capillary wedge pressure" OR PCWP) — 425 records; 419 after english[la].

A2. Embase

Field	Detail
Database and platform	Embase, Elsevier embase.com interface (alternatively Ovid Embase; syntax below is given for embase.com)
Date of last search	31 July 2026
Publication date limits	[2016-2026]/py, then manually limited to 1 February 2016 to 31 March 2026
Language restriction	AND [english]/lim
Controlled vocabulary (Emtree)	'sodium glucose cotransporter 2 inhibitor'/exp; 'heart failure'/exp; 'heart left ventricle failure'/exp; 'heart ventricle remodeling'/exp; 'echocardiography'/exp; 'heart left ventricle ejection fraction'/exp; 'brain natriuretic peptide'/exp; 'pulmonary artery wedge pressure'/exp; 'nuclear magnetic resonance imaging'/exp
Free-text fields	:ti,ab,kw with truncation (*)
RCT filter	Cochrane Handbook Embase filter: 'crossover procedure'/exp OR 'double blind procedure'/exp OR 'randomized controlled trial'/exp OR 'single blind procedure'/exp OR random*:ti,ab,kw OR factorial*:ti,ab,kw OR placebo*:ti,ab,kw OR (doubl* NEAR/1 blind*):ti,ab,kw OR (singl* NEAR/1 blind*):ti,ab,kw OR assign*:ti,ab,kw OR allocat*:ti,ab,kw OR crossover*:ti,ab,kw, then NOT ('animal'/exp NOT 'human'/exp)
Total records retrieved	to be inserted

Line	Search string (embase.com syntax)	Records
1	'sodium glucose cotransporter 2 inhibitor'/exp	50,734
2	(sglt2 NEAR/2 inhibitor*):ti,ab,kw OR 'sglt2i':ti,ab,kw OR gliflozin*:ti,ab,kw OR (sodium NEAR/2 glucose NEAR/2 cotransporter*):ti,ab,kw	31,999

3	empagliflozin:ti,ab,kw OR dapagliflozin:ti,ab,kw OR canagliflozin:ti,ab,kw OR ertugliflozin:ti,ab,kw OR ipragliflozin:ti,ab,kw OR luseogliflozin:ti,ab,kw OR tofogliflozin:ti,ab,kw OR sotagliflozin:ti,ab,kw OR bexagliflozin:ti,ab,kw	19,240
4	#1 OR #2 OR #3	55,455
5	'heart failure'/exp OR 'heart left ventricle failure'/exp	838,438
6	'heart failure':ti,ab,kw OR hfref:ti,ab,kw OR hfmref:ti,ab,kw OR hfpef:ti,ab,kw OR (reduced NEAR/2 'ejection fraction'):ti,ab,kw OR (preserved NEAR/2 'ejection fraction'):ti,ab,kw OR (mildly NEAR/3 'ejection fraction'):ti,ab,kw	473,322
7	#5 OR #6	911,307
8	'heart ventricle remodeling'/exp OR 'echocardiography'/exp OR 'heart left ventricle ejection fraction'/exp OR 'brain natriuretic peptide'/exp OR 'pulmonary artery wedge pressure'/exp OR 'nuclear magnetic resonance imaging'/exp	2,160,904
9	remodel*:ti,ab,kw OR remodell*:ti,ab,kw OR ('global longitudinal strain'):ti,ab,kw OR 'longitudinal strain':ti,ab,kw OR 'speckle tracking':ti,ab,kw OR 'myocardial work':ti,ab,kw OR 'diastolic function':ti,ab,kw OR 'e/e prime':ti,ab,kw OR 'left atrial volume':ti,ab,kw OR lavi:ti,ab,kw OR 'wedge pressure':ti,ab,kw OR pcwp:ti,ab,kw OR 'filling pressure*':ti,ab,kw OR lvedv*:ti,ab,kw OR lvesv*:ti,ab,kw OR 'ventricular mass':ti,ab,kw OR lvmi:ti,ab,kw OR 'nt-probnp':ti,ab,kw OR echocardiograph*:ti,ab,kw OR 'cardiac magnetic resonance':ti,ab,kw	735,293
10	#8 OR #9	2,799,138
11	#4 AND #7 AND #10	3,377
12	Cochrane Embase RCT filter (see above)	3,626,418
13	#11 AND #12 NOT ('animal'/exp NOT 'human'/exp)	952
14	#13 AND [english]/lim AND [2016-2026]/py	939

A3. Scopus

Field	Detail
Database and platform	Scopus, Elsevier (https://www.scopus.com), Advanced Search
Date of last search	31 July 2026
Publication date limits	AND PUBYEAR > 2015 AND PUBYEAR < 2027, then manually limited to 1 February 2016 to 31 March 2026
Language restriction	AND LANGUAGE(english)
Controlled vocabulary	Not applicable — Scopus does not index with a thesaurus
Field restrictions	TITLE-ABS-KEY( ) throughout; DOCTYPE(ar) OR DOCTYPE(re) excluded as appropriate
RCT filter	TITLE-ABS-KEY(random* OR placebo OR "double blind" OR "single blind" OR trial OR allocat* OR assign* OR crossover)
Total records retrieved	to be inserted

Line	Search string (Scopus syntax)	Records
1	TITLE-ABS-KEY("SGLT2 inhibitor*" OR "SGLT-2 inhibitor*" OR SGLT2i OR gliflozin* OR "sodium glucose cotransporter 2" OR "sodium-glucose co-transporter 2" OR empagliflozin OR dapagliflozin OR canagliflozin OR ertugliflozin OR ipragliflozin OR luseogliflozin OR tofogliflozin OR sotagliflozin OR bexagliflozin)	41,198
2	TITLE-ABS-KEY("heart failure" OR "cardiac failure" OR HFrEF OR HFmrEF OR HFpEF OR "reduced ejection fraction" OR "preserved ejection fraction" OR "mildly reduced ejection fraction" OR "left ventricular dysfunction")	511,098
3	TITLE-ABS-KEY(remodel* OR remodell* OR "global longitudinal strain" OR "longitudinal strain" OR "speckle tracking" OR "myocardial work" OR "diastolic function" OR "E/e'" OR "left atrial volume" OR LAVI OR "wedge pressure" OR PCWP OR "filling pressure*" OR LVEDV* OR LVESV* OR "ejection fraction" OR "left ventricular mass" OR LVMI OR "NT-proBNP" OR echocardiograph* OR "cardiac magnetic resonance")	921,640
4	TITLE-ABS-KEY(random* OR placebo OR "double blind" OR "single blind" OR trial OR allocat* OR assign* OR crossover)	7,595,281
5	#1 AND #2 AND #3 AND #4	3,648
6	#5 AND PUBYEAR > 2015 AND PUBYEAR < 2027 AND LANGUAGE(english)	3,490

Table S6b. Citation searching log

Every one of the 14 publications retained in the synthesis was retrieved by the reconstructed PubMed search, verified PMID by PMID against the 1203-record set (Table S5). No included publication therefore entered the review through citation searching.

Publication	Source article whose reference list identified it	Stage at which it entered screening	Documented evidence supporting classification as identified through reference searching	Final classification
— none —	Not applicable	Not applicable	no publication has been classified as identified through reference searching	Identified through database searching

Table S6c. Validation table — retrieval of each included study by each database

Study	First author, year	PMID	DOI	Retrieved in PubMed	Retrieved in Embase	Retrieved in Scopus	Evidence
EMPA-TROPISM	Santos-Gallego 2021	33197559	10.1016/j.jacc.2020.11.008	Yes	yes	yes	Retrieved by line #19 of the reconstructed PubMed strategy
SUGAR-DM-HF	Lee 2021	33186500	10.1161/CIRCULATIONAHA.120.052186	Yes	yes	yes	Retrieved by line #19 of the reconstructed PubMed strategy
Empire HF	Omar 2021	33404637	10.1001/jamacardio.2020.6827	Yes	yes	yes	Retrieved by line #19 of the reconstructed PubMed strategy
Empire HF	Omar 2020	33272368	10.1016/j.jacc.2020.10.005	Yes	yes	yes	Retrieved by line #19 of the reconstructed PubMed strategy
REFORM	Singh 2020	32245746	10.2337/dc19-2187	Yes	no	no	Retrieved by line #19 of the reconstructed PubMed strategy
EMPA-VISION	Hundertmark 2023	37070436	10.1161/CIRCULATIONAHA.122.062021	Yes	yes	yes	Retrieved by line #19 of the reconstructed PubMed strategy
DAPA-ECHO	Pastore 2024	39023499	10.1016/j.jcmg.2024.05.014	Yes	yes	yes	Retrieved by line #19 of the reconstructed PubMed strategy
Reis 2022	Reis 2022	35629062	10.3390/jcm11102935	Yes	yes	yes	Retrieved by line #19 of the reconstructed PubMed strategy
Fu 2023	Fu 2023	37940879	10.1186/s12872-023-03591-3	Yes	yes	yes	Retrieved by line #19 of the reconstructed PubMed strategy
EFFORT	Kang 2024	38690659	10.1161/CIRCULATIONAHA.124.069144	Yes	no	yes	Retrieved by line #19 of the reconstructed PubMed strategy
CANDLE	Tanaka 2020	32349193	10.1002/ehf2.12707	Yes	yes	yes	Retrieved by line #19 of the reconstructed PubMed strategy
CAMEO-DAPA	Borlaug 2023	37534453	10.1161/CIRCULATIONAHA.123.065134	Yes	yes	yes	Retrieved by line #19 of the reconstructed PubMed strategy
Ovchinnikov 2025	Ovchinnikov 2025	40346546	10.1186/s12933-025-02756-y	Yes	yes	yes	Retrieved by line #19 of the reconstructed PubMed strategy
EXCEED	Akasaka 2022	35212104	10.1111/ggi.14363	Yes	yes	yes	Retrieved by line #19 of the reconstructed PubMed strategy

All 14 retained publications were retrieved in PubMed, 13 retained from Scopus, 12 from EMBASE.

Table S7. Cohen's kappa with bounded 95% confidence intervals

Handling of "Maybe" decisions. Screening decisions were recorded as Include, Exclude or Maybe. Because the operational consequence at the title/abstract stage is binary, decisions were dichotomised as "retain for full-text assessment" (Include or Maybe) versus "exclude" before computing kappa, consistent with the protocol rule that a Maybe from either reviewer sends the record to full text. No record was discarded from the calculation.

Confidence interval method. Non-parametric bootstrap with 20 000 resamples of the paired reviewer decisions (seed 20260730); percentile and bias-corrected accelerated (BCa) intervals are both reported, with BCa used in the manuscript. Every bootstrap replicate of kappa is itself bounded above by 1.00, so the interval cannot exceed 1.00 and no truncation is applied. This replaces the Fleiss large-sample normal approximation used in the submitted version, which produced an upper limit of 1.069 at the full-text stage — an impossible value for a bounded statistic.

Screening stage	n	a both retain	b R1 retain / R2 exclude	c R1 exclude / R2 retain	d both exclude	Discordant (b + c)	Observed agreement Po	Expected agreement Pe	Cohen's kappa	95% CI percentile	95% CI BCa	Interpretation
Title/abstract screening	2836	281	2	1	2552	3	0.9989	0.8206	0.994	0.986 to 1.000	0.983 to 0.998	Almost perfect
Full-text eligibility assessment	283	16	1	1	265	2	0.9929	0.8871	0.937	0.829 to 1.000	0.758 to 1.000	Almost perfect

The discordant cell count at each stage now equals exactly the number of rows recorded for that stage in Table S4: three at title/abstract and two at full text. In the submitted version the title/abstract table reported five discordant decisions, which conflated all five rows of the discrepancy log across both stages, while the full-text table reported only one although two full-text disagreements were logged.

Provenance. The concordant cells are derived from the reconstructed consensus decisions and the discordant cells from the documented discrepancy log, which is the primary surviving record of pre-consensus disagreement. If the original Rayyan export remains available, the review team should re-derive both agreement tables directly from it and substitute those values.

Table S8. RoB 2 signalling-question responses and written justifications

Target of measurement. For every study and every outcome the effect of interest is the EFFECT OF ASSIGNMENT to intervention (the intention-to-treat effect). This target was chosen because the review question concerns whether starting an SGLT2 inhibitor changes cardiac structure, function and loading, not whether perfect adherence would do so. Where a trial analysed only participants with complete paired imaging, that departure is recorded under Domain 3 and, where the analysis set cannot estimate the assignment effect, also under Domain 2.

Risk of bias was assessed separately for three outcome strata because the domains that threaten validity differ by outcome type: volumetric and structural imaging outcomes; functional and loading outcomes (strain, E/e-prime, LAVi, PCWP), which are operator-dependent and load-dependent; and the centrally assayed neurohormonal biomarker.

EMPA-TROPISM — Santos-Gallego 2021 (PMID 33197559)

Domain	Signalling-question responses
D1 Bias arising from the randomisation process	1.1 Yes - randomised allocation stated. 1.2 No information on the concealment mechanism; the trial was double-blind and placebo-controlled, which makes subversion unlikely. 1.3 No - baseline CMR and clinical variables were balanced.
D2 Bias due to deviations from intended interventions	2.1 No - participants were blinded. 2.2 No - investigators were blinded. 2.3 No evidence of context-driven deviations. 2.6 Yes - analysis compared randomised groups. 2.7 Four participants were not analysable because of implantable-device artefact, a small and non-differential loss.
D3 Bias due to missing outcome data	3.1 No - 80 of 84 randomised participants (95%) contributed CMR data. 3.2 Yes - loss was for a technical imaging reason unrelated to outcome. 3.3 No.
D4 Bias in measurement of the outcome	4.1 No - CMR is the reference standard for volumes and mass. 4.2 No - identical protocol in both arms. 4.3 No - readers were blinded. 4.4/4.5 Not applicable.
D5 Bias in selection of the reported result	5.1 Probably yes - LVEDV and LVESV were the registered primary endpoints (NCT03485222). 5.2 Probably no for the primary volumes. 5.3 Some concern: the publication reports only arm-level change scores with a between-arm p value and no adjusted treatment effect or confidence interval, so the reader cannot verify which analysis model produced the p value.

Outcome stratum	D1	D2	D3	D4	D5	Overall	Justification
Volumetric and structural imaging outcomes (LVEDV/i, LVESV/i, LVEF, LV mass / LVMi)	Low	Low	Low	Low	Some concerns	Some concerns	Well-conducted double-blind placebo-controlled CMR trial with blinded core-laboratory reading and near-complete follow-up. The single reservation is reporting: no adjusted between-group effect estimate or confidence interval is published for any outcome, only arm-level change scores with a between-arm p value, which limits verification of the analysis actually performed.
Functional and loading outcomes (GLS, E/e-prime, LAVi, PCWP, atrial strain)	Low	Low	Low	Low	Some concerns	Some concerns	As for volumetric outcomes. Strain was not reported.
Neurohormonal biomarker (NT-proBNP)	Low	Low	Low	Low	Some concerns	Some concerns	NT-proBNP was a secondary outcome reported as a percentage change per arm without an adjusted between-group estimate.

SUGAR-DM-HF — Lee 2021 (PMID 33186500)

Domain	Signalling-question responses
D1 Bias arising from the randomisation process	1.1 Yes. 1.2 Yes - central web-based randomisation with concealed allocation. 1.3 No - groups were balanced on baseline CMR and clinical variables.
D2 Bias due to deviations from intended interventions	2.1 No - double-blind. 2.2 No - double-blind. 2.3 No. 2.6 Yes - analysis of covariance comparing randomised groups, adjusted for baseline value, age and diabetes status.
D3 Bias due to missing outcome data	3.1 Probably no - 92 of 105 randomised participants (88%) had evaluable paired CMR (42 of 52 empagliflozin, 50 of 53 placebo). 3.2 Probably yes - reasons were technical or withdrawal, reported and broadly similar. 3.3 Probably no, although the loss was somewhat greater in the empagliflozin arm (19% versus 6%), which is the reason this domain is not rated Low.
D4 Bias in measurement of the outcome	4.1 No - CMR with core-laboratory analysis. 4.2 No. 4.3 No - blinded analysts. 4.4/4.5 Not applicable.
D5 Bias in selection of the reported result	5.1 Yes - LVESVi and LV GLS were registered co-primary endpoints with a published statistical analysis plan (NCT03485092). 5.2 No. 5.3 No - a prespecified sensitivity analysis is also reported.

Outcome stratum	D1	D2	D3	D4	D5	Overall	Justification
Volumetric and structural imaging outcomes (LVEDV/i, LVESV/i, LVEF, LV mass / LVMi)	Low	Low	Some concerns	Low	Low	Some concerns	Methodologically the strongest CMR trial in the review: concealed central randomisation, double-blind placebo control, prespecified co-primary endpoints, blinded core-laboratory reading and adjusted between-group estimates with confidence intervals. Attrition of 12% overall, unevenly distributed (19% versus 6%), prevents a Low rating for missing data.
Functional and loading outcomes (GLS, E/e-prime, LAVi, PCWP, atrial strain)	Low	Low	Some concerns	Low	Low	Some concerns	As above. GLS was a co-primary endpoint measured by CMR feature tracking.
Neurohormonal biomarker (NT-proBNP)	Low	Low	Low	Low	Low	Low	NT-proBNP was a prespecified secondary endpoint available in all 94 participants with blood sampling and analysed on the log scale with an adjusted between-group ratio.

Empire HF (echocardiographic substudy) — Omar 2021 (PMID 33404637)

Domain	Signalling-question responses
D1 Bias arising from the randomisation process	1.1 Yes. 1.2 Yes - the parent Empire HF trial used concealed central randomisation. 1.3 No.
D2 Bias due to deviations from intended interventions	2.1 No - double-blind. 2.2 No. 2.3 No. 2.6 Yes.
D3 Bias due to missing outcome data	3.1 No - 186 of 190 randomised participants completed; echocardiographic data were available in 87 to 95 participants per arm depending on the measurement. 3.2 Probably yes. 3.3 Probably no. Denominators differ by measurement and are reported.
D4 Bias in measurement of the outcome	4.1 No - two-dimensional echocardiography with core-laboratory analysis. 4.2 No. 4.3 No - blinded readers. 4.5 No.
D5 Bias in selection of the reported result	5.1 NO - the publication states explicitly that echocardiographic measurements were not prespecified in the parent trial and describes itself as an exploratory post hoc analysis. 5.2 Probably yes - several volumetric, mass, strain and geometric measures were available and reported. 5.3 Probably yes - unadjusted and adjusted models are both presented, with the significance of LVESVI and LAVI depending on which model is chosen.

Outcome stratum	D1	D2	D3	D4	D5	Overall	Justification
Volumetric and structural imaging outcomes (LVEDV/i, LVESV/i, LVEF, LV mass / LVMi)	Low	Low	Some concerns	Low	High	High	This is an explicitly EXPLORATORY POST HOC analysis of a trial whose registered primary endpoint was NT-proBNP; the publication states that the echocardiographic measurements were not prespecified. The nominal significance of LVESVI and LAVI is model-dependent (LVESVI -4.3 mL/m2, P=0.04 in the primary model but -4.0 mL/m2, P=0.06 after further adjustment; LAVI -2.5, P=0.04 falling to -2.2, P=0.07). Domain 5 is therefore High risk and drives the overall judgement. The submitted manuscript rated this report low risk overall, which is not defensible.
Functional and loading outcomes (GLS, E/e-prime, LAVi, PCWP, atrial strain)	Low	Low	Some concerns	Low	High	High	As above; applies to GLS and LAVi.
Neurohormonal biomarker (NT-proBNP)	Low	Low	Low	Low	Low	Low	NT-proBNP was the registered primary endpoint of the parent trial and is not affected by the post hoc nature of the imaging substudy.

Empire HF (haemodynamic substudy) — Omar 2020 (PMID 33272368)

Domain	Signalling-question responses
D1 Bias arising from the randomisation process	1.1 Yes. 1.2 Yes. 1.3 Probably yes - the publication notes significant baseline differences between arms in peak-exercise diastolic pulmonary artery pressure and PCWP, the very variables that form the outcome.
D2 Bias due to deviations from intended interventions	2.1 No - double-blind. 2.2 No. 2.3 No. 2.6 Yes - linear mixed model on randomised groups.
D3 Bias due to missing outcome data	3.1 No - 66 of 70 randomised participants had paired right-heart catheterisation (94%). 3.2 Yes. 3.3 No.
D4 Bias in measurement of the outcome	4.1 No - invasive high-fidelity pressure measurement. 4.2 No. 4.3 No. 4.5 No.
D5 Bias in selection of the reported result	5.1 Yes for the primary endpoint (peak-exercise PCWP/cardiac index). 5.2 Probably yes for the secondary haemodynamic outcomes: PCWP is reported at rest, at peak exercise and pooled across all exercise loads, and only the pooled analysis is significant. 5.3 Probably yes - the pooled across-load analysis is described in the paper as a post hoc analysis using exercise load as a covariate.

Outcome stratum	D1	D2	D3	D4	D5	Overall	Justification
Volumetric and structural imaging outcomes (LVEDV/i, LVESV/i, LVEF, LV mass / LVMi)	Some concerns	Low	Low	Low	High	High	The registered primary endpoint (peak-exercise PCWP/cardiac index) was NEUTRAL. The PCWP reduction quoted in the review (-2.40 mmHg across all exercise loads) comes from an analysis the authors themselves label post hoc, while resting PCWP (-1.47 mmHg, P=0.23) and peak-exercise PCWP were not significant between groups. Baseline imbalance in exercise PCWP compounds the concern. Domain 5 is High risk for the haemodynamic outcomes.
Functional and loading outcomes (GLS, E/e-prime, LAVi, PCWP, atrial strain)	Some concerns	Low	Low	Low	High	High	As above. The end-diastolic pressure-volume relationship result is a WITHIN-GROUP change with no significant between-group treatment effect and cannot support an inference about treatment.
Neurohormonal biomarker (NT-proBNP)	Some concerns	Low	Low	Low	Some concerns	Some concerns	Plasma volume and haematocrit were prespecified exploratory endpoints with adjusted between-group estimates.

REFORM — Singh 2020 (PMID 32245746)

Domain	Signalling-question responses
D1 Bias arising from the randomisation process	1.1 Yes - 1:1 randomisation. 1.2 No information on the concealment mechanism. 1.3 No information - baseline echocardiographic and CMR values are relegated to a supplementary table that is not reproduced in the four-page report, so baseline comparability cannot be verified.
D2 Bias due to deviations from intended interventions	2.1 No - placebo-controlled. 2.2 No. 2.3 No. 2.6 Yes - intention-to-treat analysis.

D3 Bias due to missing outcome data	3.1 Yes for the analysis as performed, but missing values were handled by SINGLE MEAN IMPUTATION, which understates uncertainty and is not a defensible method for a continuous primary outcome. 3.2 No. 3.3 No information on the amount or pattern of missingness.
D4 Bias in measurement of the outcome	4.1 No - CMR. 4.2 No. 4.3 Probably no. 4.5 No.
D5 Bias in selection of the reported result	5.1 Probably yes - LVESV was the registered primary endpoint (NCT02397421). 5.2 No. 5.3 No. The report does, however, contain an internal numerical inconsistency: the LVEF treatment effect is 0.69 in Table 1 and 0.96 in the Results text.

Outcome stratum	D1	D2	D3	D4	D5	Overall	Justification
Volumetric and structural imaging outcomes (LVEDV/i, LVESV/i, LVEF, LV mass / LVMi)	Some concerns	Low	Some concerns	Low	Some concerns	Some concerns	A neutral double-blind CMR trial. Concerns arise from the very brief report: no allocation-concealment detail, no baseline imaging values, single mean imputation for missing data, no stated ejection-fraction entry threshold, and an internal inconsistency in the reported LVEF effect. None of these is likely to have manufactured a false negative, so the rating is Some concerns rather than High.
Functional and loading outcomes (GLS, E/e-prime, LAVi, PCWP, atrial strain)	Some concerns	Low	Some concerns	Low	Some concerns	Some concerns	As above; applies to LAVi. Strain and E/e-prime were not reported.
Neurohormonal biomarker (NT-proBNP)	Some concerns	Low	Some concerns	Low	Some concerns	Some concerns	NT-proBNP outcome data are not reported in the primary publication.

EMPA-VISION (HFrEF cohort) — Hundertmark 2023 (PMID 37070436)

Domain	Signalling-question responses
D1 Bias arising from the randomisation process	1.1 Yes. 1.2 Probably yes - double-blind placebo-controlled design. 1.3 No for the HFrEF cohort.
D2 Bias due to deviations from intended interventions	2.1 No - double-blind. 2.2 No. 2.3 No. 2.6 NO for the analysis presented: the CMR outcomes were analysed on the PER-PROTOCOL SET, which estimates neither the effect of assignment nor a properly defined per-protocol effect. 2.7 The impact is likely small in this cohort (35 of 36 analysed) but the analysis population is not the randomised population.
D3 Bias due to missing outcome data	3.1 Probably yes - 35 of 36 randomised HFrEF participants. 3.2 Yes. 3.3 No.
D4 Bias in measurement of the outcome	4.1 No - CMR with feature tracking for strain. 4.2 No. 4.3 No. 4.5 No.
D5 Bias in selection of the reported result	5.1 Yes for the primary endpoint (cardiac PCr/ATP, NCT03332212). 5.2 Probably yes - the CMR volumetric, mass and multidirectional strain outcomes are all labelled EXPLORATORY in the tables and are numerous (three strain directions, two strain-rate directions, torsion, volumes, mass, mass index, tissue characterisation), with no adjustment for multiplicity. 5.3 Probably yes.

Outcome stratum	D1	D2	D3	D4	D5	Overall	Justification
Volumetric and structural imaging outcomes (LVEDV/i, LVESV/i, LVEF, LV mass / LVMi)	Low	Some concerns	Low	Low	High	High	A mechanistic trial whose registered primary endpoint (PCr/ATP) was neutral. Every CMR outcome used by this review is explicitly labelled exploratory, analysed on the per-protocol set, and drawn from a large panel of correlated measures without multiplicity control. The nominally significant LV mass and mass index results must be read in that light.
Functional and loading outcomes (GLS, E/e-prime, LAVi, PCWP, atrial strain)	Low	Some concerns	Low	Low	High	High	As above; applies to CMR feature-tracking strain.
Neurohormonal biomarker (NT-proBNP)	Low	Some concerns	Low	Low	Some concerns	Some concerns	Serum biomarkers were prespecified secondary outcomes; most were unchanged.

EMPA-VISION (HFpEF cohort) — Hundertmark 2023 (PMID 37070436)

Domain	Signalling-question responses
D1 Bias arising from the randomisation process	1.1 Yes. 1.2 Probably yes. 1.3 YES - a material baseline imbalance is present in the HFpEF per-protocol cohort: LVEF 52.6% (empagliflozin) versus 59.1% (placebo) and LVEDV 174 mL versus 122 mL. Because the analysed cohort is only 13 versus 11, this imbalance plausibly reflects the attrition rather than the randomisation, but it undermines comparability either way.
D2 Bias due to deviations from intended interventions	2.1 No - double-blind. 2.2 No. 2.3 No. 2.6 NO - per-protocol analysis set. 2.7 The impact is substantial: only 24 of 36 randomised HFpEF participants (67%) are analysed.
D3 Bias due to missing outcome data	3.1 NO - only 13 of 18 and 11 of 18 randomised participants contributed CMR data (67% overall). 3.2 No - the report does not demonstrate that the result is unaffected. 3.3 Probably yes - loss of a third of the cohort in a mechanistic imaging trial may relate to tolerability or disease severity. 3.4 Cannot be excluded.
D4 Bias in measurement of the outcome	4.1 No - CMR. 4.2 No. 4.3 No. 4.5 No.
D5 Bias in selection of the reported result	5.1 Yes for PCr/ATP. 5.2 Probably yes - exploratory panel as for the HFrEF cohort. 5.3 Probably yes.

Outcome stratum	D1	D2	D3	D4	D5	Overall	Justification

Volumetric and structural imaging outcomes (LVEDV/i, LVESV/i, LVEF, LV mass / LVMi)	High	High	High	Low	High	High	The HFpEF cohort is the weakest evidence in the review and was previously rated as low risk or some concerns. Only 24 of 36 randomised participants were analysed, on a per-protocol basis, with marked baseline imbalance in LVEF and LVEDV between the analysed arms, and every CMR outcome is exploratory. High risk of bias in three domains.
Functional and loading outcomes (GLS, E/e-prime, LAVi, PCWP, atrial strain)	High	High	High	Low	High	High	As above. The longitudinal-strain result additionally carries a direction error in the source publication: strain became LESS negative with empagliflozin, so the positive adjusted difference of +2.18% denotes numerically worse, not improved, strain, and it is not significant.
Neurohormonal biomarker (NT-proBNP)	High	High	High	Low	Some concerns	High	As above.

DAPA-ECHO — Pastore 2024 (PMID 39023499)

Domain	Signalling-question responses
D1 Bias arising from the randomisation process	1.1 Yes - block randomisation, block size 4. 1.2 Probably yes - allocation generated by the statistical software. 1.3 No - clinical and echocardiographic characteristics were comparable.
D2 Bias due to deviations from intended interventions	2.1 YES - open-label; participants knew they were receiving dapagliflozin, and the comparator received no placebo. 2.2 YES - treating clinicians were aware. 2.3 Probably yes - in an open-label trial of a guideline-recommended therapy, awareness may influence adherence to background therapy, up-titration and follow-up intensity; none of this is reported. 2.4 Possibly, for strain outcomes that are load-dependent. 2.5 Not reported. 2.6 Yes - linear mixed model on randomised groups.
D3 Bias due to missing outcome data	3.1 Yes - all 88 randomised participants analysed. 3.2 Yes. 3.3 No.
D4 Bias in measurement of the outcome	4.1 No - speckle-tracking echocardiography with excellent reported reproducibility (intraclass correlation 0.95 to 0.99). 4.2 No. 4.3 NOT REPORTED - the publication does not state that the strain analysts were blinded to allocation. 4.4 Yes - strain analysis involves operator decisions about tracking and segment acceptance and is susceptible to expectation. 4.5 Cannot be excluded.
D5 Bias in selection of the reported result	5.1 Probably yes - GLS and atrial strain were the registered primary endpoints (NCT05179668). 5.2 No. 5.3 Some concern - the tables present within-group change scores with within-group p values and no adjusted between-group mean difference or confidence interval; the headline P<0.0001 is a mixed-model main treatment effect that cannot be checked against a point estimate.

Outcome stratum	D1	D2	D3	D4	D5	Overall	Justification
Volumetric and structural imaging outcomes (LVEDV/i, LVESV/i, LVEF, LV mass / LVMi)	Low	Some concerns	Low	Some concerns	Some concerns	Some concerns	Volumetric outcomes were neutral, so the open-label design and unstated reader blinding matter less.
Functional and loading outcomes (GLS, E/e-prime, LAVi, PCWP, atrial strain)	Low	High	Low	High	Some concerns	High	The strain findings are the review largest reported functional effects and they come from an open-label single-centre trial with no placebo, no statement that strain readers were blinded, and no adjusted between-group estimate. Domain 4 is therefore High risk for strain and diastolic outcomes.
Neurohormonal biomarker (NT-proBNP)	Low	Some concerns	Low	Low	Some concerns	Some concerns	NT-proBNP is laboratory-measured and less susceptible to assessor expectation; the between-group difference was not significant.

Reis 2022 (dapagliflozin and exercise capacity) — Reis 2022 (PMID 35629062)

Domain	Signalling-question responses
D1 Bias arising from the randomisation process	1.1 Yes - 1:1 randomisation stratified by sex, LVEF and heart-failure aetiology. 1.2 No information on concealment. 1.3 No - baseline LVEF, NT-proBNP and peak oxygen uptake were similar.
D2 Bias due to deviations from intended interventions	2.1 YES - open-label, control arm continued existing therapy with no placebo. 2.2 YES. 2.3 Probably yes and of particular concern here: the control arm DETERIORATED markedly over six months (GLS -8.4% to -6.8%, LAVi 41.9 to 48.1 mL/m2, pulmonary artery systolic pressure 29.8 to 39.4 mmHg), a pattern not expected in stable ambulatory heart failure on optimal therapy and consistent with differential management or measurement between arms. 2.6 Analysis compared randomised groups by repeated-measures ANOVA.
D3 Bias due to missing outcome data	3.1 Yes - 40 of 40 analysed. 3.2 Yes. 3.3 No.
D4 Bias in measurement of the outcome	4.1 No - echocardiography and speckle tracking. 4.2 Not reported. 4.3 NOT REPORTED - no statement that echocardiographic readers were blinded. 4.4 Yes. 4.5 Likely, given the implausible control-arm trajectory.
D5 Bias in selection of the reported result	5.1 NO - the trial was not prospectively registered. 5.2 Probably yes - a large panel of clinical, echocardiographic, biomarker and exercise variables is reported without multiplicity control. 5.3 Probably yes - results are presented as time, group and interaction p values with no effect estimates or confidence intervals for any outcome.

Outcome stratum	D1	D2	D3	D4	D5	Overall	Justification
Volumetric and structural imaging outcomes (LVEDV/i, LVESV/i, LVEF, LV mass / LVMi)	Some concerns	High	Low	High	High	High	Small (n=40), single-centre, open-label, unregistered trial with no placebo, no statement of reader blinding, no effect estimates or confidence intervals, and a control arm that deteriorated implausibly across nearly every measure. LV volumes were not reported at all - only linear dimensions.

Functional and loading outcomes (GLS, E/e-prime, LAVi, PCWP, atrial strain)	Some concerns	High	Low	High	High	High	As above. The nominally very large GLS, E/e-prime and LAVi interaction effects are substantially driven by control-arm deterioration rather than by improvement in the treated arm, and should carry very little weight.
Neurohormonal biomarker (NT-proBNP)	Some concerns	High	Low	Some concerns	High	High	The NT-proBNP interaction is likewise driven by a rise in the control arm (+650 pg/mL) rather than a fall in the treated arm (-218 pg/mL).

Fu 2023 — Fu 2023 (PMID 37940879)

Domain	Signalling-question responses
D1 Bias arising from the randomisation process	1.1 Yes - 1:1 randomisation. 1.2 Yes - block randomisation, block size 4, with CONCEALED allocation explicitly described. 1.3 No information - baseline echocardiographic values by arm are not fully tabulated.
D2 Bias due to deviations from intended interventions	2.1 No - double-blind, placebo-controlled. 2.2 No. 2.3 No. 2.6 Yes - all randomised participants who received at least one dose were analysed.
D3 Bias due to missing outcome data	3.1 Yes - 60 of 60 analysed. 3.2 Yes. 3.3 No.
D4 Bias in measurement of the outcome	4.1 No - transthoracic echocardiography with Doppler. 4.2 No. 4.3 Probably no - double-blind design. 4.5 No.
D5 Bias in selection of the reported result	5.1 Probably yes - change in LVEF was the stated primary outcome and the trial is registered (ChiCTR2300072707). 5.2 No. 5.3 No - adjusted between-group mean differences with confidence intervals are reported for every outcome. However, registration POSTDATES enrolment (registered 21 June 2023 for a 12-month trial reported in 2023), so prospective specification cannot be confirmed.

Outcome stratum	D1	D2	D3	D4	D5	Overall	Justification
Volumetric and structural imaging outcomes (LVEDV/i, LVESV/i, LVEF, LV mass / LVMi)	Some concerns	Low	Low	Low	Some concerns	Some concerns	Double-blind placebo-controlled trial with concealed block randomisation and properly reported adjusted between-group estimates with confidence intervals. Two reservations: baseline echocardiographic values by arm are incompletely reported, and the trial registration appears retrospective.
Functional and loading outcomes (GLS, E/e-prime, LAVi, PCWP, atrial strain)	Some concerns	Low	Low	Low	Some concerns	Some concerns	Strain, E/e-prime and LAVi were not reported.
Neurohormonal biomarker (NT-proBNP)	Some concerns	Low	Low	Low	High	High	NT-proBNP was an entry criterion (>600 pg/mL) and a baseline characteristic, but NO NT-proBNP outcome result is reported.

EFFORT — Kang 2024 (PMID 38690659)

Domain	Signalling-question responses
D1 Bias arising from the randomisation process	1.1 Yes. 1.2 Probably yes - multicentre double-blind placebo-controlled trial. 1.3 Probably no, though hypertension (51.6% versus 38.1%) and the mechanism of mitral regurgitation were somewhat unevenly distributed.
D2 Bias due to deviations from intended interventions	2.1 No - double-blind. 2.2 No. 2.3 No. 2.6 Yes.
D3 Bias due to missing outcome data	3.1 Yes - 128 of 128 randomised participants analysed. 3.2 Yes. 3.3 No.
D4 Bias in measurement of the outcome	4.1 No - echocardiography with core-laboratory quantification of regurgitant orifice area and strain. 4.2 No. 4.3 No - blinded core laboratory. 4.5 No.
D5 Bias in selection of the reported result	5.1 Yes - change in effective regurgitant orifice area was the registered primary endpoint (NCT04739215) with between-group differences prespecified in the protocol. 5.2 No. 5.3 No. A source-level reporting inconsistency exists for LAVi, where the published 95% CI (-12.16 to 0.15) crosses zero although P is given as 0.005.

Outcome stratum	D1	D2	D3	D4	D5	Overall	Justification
Volumetric and structural imaging outcomes (LVEDV/i, LVESV/i, LVEF, LV mass / LVMi)	Low	Low	Low	Low	Low	Low	A well-conducted, adequately powered, multicentre, double-blind, placebo-controlled trial with complete follow-up, blinded core-laboratory reading, a registered primary endpoint and prespecified between-group analyses. Volumetric outcomes were neutral.
Functional and loading outcomes (GLS, E/e-prime, LAVi, PCWP, atrial strain)	Low	Low	Low	Low	Some concerns	Some concerns	The GLS result is robust. The LAVi result is downgraded because the published confidence interval is incompatible with the published p value, an unresolved reporting error in the source.
Neurohormonal biomarker (NT-proBNP)	Low	Low	Low	Low	Low	Low	NT-proBNP was a prespecified secondary endpoint; no significant between-group difference.

CANDLE — Tanaka 2020 (PMID 32349193)

Domain	Signalling-question responses
D1 Bias arising from the randomisation process	1.1 Yes - randomised with stratification by LVEF (<40% / ≥ 40%) at screening. 1.2 Probably yes - multicentre PROBE design with central registration (UMIN000017669). 1.3 No.
D2 Bias due to deviations from intended interventions	2.1 YES - open-label; the comparator was active (glimepiride), so both arms received a glucose-lowering drug, which mitigates but does not remove performance bias. 2.2 YES. 2.3 Probably no - a PROBE design with protocolised therapy. 2.6 Yes - full analysis set.
D3 Bias due to missing outcome data	3.1 Yes for NT-proBNP - 233 of 233 analysed. 3.2 Yes. 3.3 No.
D4 Bias in measurement of the outcome	4.1 No - central laboratory NT-proBNP; blinded endpoint adjudication by design. 4.2 No. 4.3 No for the primary endpoint. 4.4. Probably Yes; LVEF and particularly E/e′ involve acquisition and measurement decisions and are more operator-dependent than a centrally measured laboratory biomarker. 4.5 No. No information<br><br>The publication provides no evidence that knowledge of allocation actually influenced echocardiographic measurements.
D5 Bias in selection of the reported result	5.1 Yes - the non-inferiority primary endpoint and margin were prespecified. 5.2 No for the primary endpoint. 5.3 YES for the results actually used by this review: the NT-proBNP subgroup analyses by baseline concentration and by LVEF stratum are post hoc, are numerous, and are the only nominally significant findings in a trial whose primary endpoint was not met. Note also that randomisation was stratified at LVEF 40% whereas the reported subgroups are split at 50%.

Outcome stratum	D1	D2	D3	D4	D5	Overall	Justification
Volumetric and structural imaging outcomes (LVEDV/i, LVESV/i, LVEF, LV mass / LVMi)	Low	Some concerns	Low	Some concerns	High	High	The trial did not meet its prespecified non-inferiority primary endpoint. Every result carried forward into this review is a post hoc subgroup analysis, and the LVEF subgroup boundary (50%) differs from the randomisation stratification boundary (40%).
Functional and loading outcomes (GLS, E/e-prime, LAVi, PCWP, atrial strain)	Low	Some concerns	Low	Some concerns	High	High	Echocardiographic assessment of LVEF and E/e′ was performed at each participating site using the same assessment schedule for both treatment groups, and the measurement methods were appropriate. However, although CANDLE was described as a PROBE (open-label, blinded-endpoint) trial, the publication does not explicitly state that echocardiographers or image readers were blinded to treatment allocation. Because LVEF and particularly E/e′ involve operator-dependent acquisition and measurement, some potential for assessor-related bias cannot be excluded.
Neurohormonal biomarker (NT-proBNP)	Low	Some concerns	Low	Low	High	High	Primary NT-proBNP endpoint not met (ratio 0.48, 95% CI 0.13 to 1.59, P=0.226); the overall adjusted between-group difference was also non-significant (P=0.087). Only post hoc subgroups reached nominal significance.

CAMEO-DAPA — Borlaug 2023 (PMID 37534453)

Domain	Signalling-question responses
D1 Bias arising from the randomisation process	1.1 Yes. 1.2 Probably yes - double-blind placebo-controlled single-centre trial. 1.3 No - baseline haemodynamics and characteristics were comparable.
D2 Bias due to deviations from intended interventions	2.1 No - double-blind. 2.2 No. 2.3 No. 2.6 Yes - mixed model on randomised groups.
D3 Bias due to missing outcome data	3.1 Probably no - 38 of 43 randomised participants were treated and analysed (88%), and the per-outcome denominators vary appreciably (placebo n=14 to 17 versus dapagliflozin n=21) because invasive measurements were not obtainable in every participant. 3.2 Probably yes - reasons are documented (failure to meet haemodynamic eligibility, COVID-19, withdrawal). 3.3 Probably no, but the differential loss falls entirely on the placebo arm.
D4 Bias in measurement of the outcome	4.1 No - invasive high-fidelity micromanometry, the reference standard for filling pressure. 4.2 No. 4.3 No. 4.5 No.
D5 Bias in selection of the reported result	5.1 Yes - the composite rest-and-exercise PCWP primary endpoint and its analysis were prespecified (NCT04730947). 5.2 No. 5.3 No for the primary endpoint. NT-proBNP is explicitly labelled exploratory.

Outcome stratum	D1	D2	D3	D4	D5	Overall	Justification
Volumetric and structural imaging outcomes (LVEDV/i, LVESV/i, LVEF, LV mass / LVMi)	Low	Low	Some concerns	Low	Low	Some concerns	Volumetric outcomes were not a focus. Differential loss of invasive measurements in the placebo arm prevents a Low rating for missing data.
Functional and loading outcomes (GLS, E/e-prime, LAVi, PCWP, atrial strain)	Low	Low	Some concerns	Low	Low	Some concerns	The strongest haemodynamic evidence in the review: prespecified primary endpoint, met, measured invasively under double-blind conditions. The only reservation is that per-outcome denominators fall to 14 of 20 randomised placebo participants for some pressures.
Neurohormonal biomarker (NT-proBNP)	Low	Low	Some concerns	Low	Some concerns	Some concerns	NT-proBNP was an exploratory outcome analysed as a median change with a rank-sum test; the between-group difference reflects mainly a rise in the placebo arm (+22 pg/mL) rather than a fall with dapagliflozin (-7 pg/mL).

Ovchinnikov 2025 — Ovchinnikov 2025 (PMID 40346546)

Domain	Signalling-question responses

D1 Bias arising from the randomisation process	1.1 Yes - randomised 1:1. 1.2 No information on the concealment mechanism. 1.3 No - baseline clinical and echocardiographic variables were comparable.
D2 Bias due to deviations from intended interventions	2.1 YES - open-label; the control arm received standard therapy without placebo. 2.2 YES. 2.3 Probably yes - awareness may influence diuretic and background-therapy adjustment, which is directly relevant to filling-pressure surrogates such as E/e-prime and LAVi, and to the 6-minute walk primary endpoint. 2.6 Yes - randomised groups compared with adjusted between-group differences and confidence intervals.
D3 Bias due to missing outcome data	3.1 Yes - 70 of 70 randomised participants analysed. 3.2 Yes. 3.3 No.
D4 Bias in measurement of the outcome	4.1 No - resting and exercise echocardiography with strain. 4.2 No. 4.3 NOT REPORTED - the publication does not state that readers were blinded to allocation. 4.4 Yes - E/e-prime, atrial strain and exercise reserve measures all involve operator judgement. 4.5 Cannot be excluded. The primary endpoint (6-minute walk distance) is additionally effort-dependent and unblinded.
D5 Bias in selection of the reported result	5.1 Probably yes - a registered protocol with a prespecified primary endpoint and prespecified secondary endpoints is described. 5.2 Probably yes - a very large panel of resting and exercise measures and reserves is reported without multiplicity control. 5.3 No - adjusted between-group differences with confidence intervals are reported throughout.

Outcome stratum	D1	D2	D3	D4	D5	Overall	Justification
Volumetric and structural imaging outcomes (LVEDV/i, LVESV/i, LVEF, LV mass / LVMi)	Some concerns	Some concerns	Low	Some concerns	Some concerns	Some concerns	Volumetric outcomes and GLS were neutral, so the risk of bias matters less for them.
Functional and loading outcomes (GLS, E/e-prime, LAVi, PCWP, atrial strain)	Some concerns	High	Low	High	Some concerns	High	The E/e-prime, LAVi and exercise-reserve findings are the principal HFpEF diastolic evidence in the review, and they come from an open-label single-centre trial with no placebo and no statement of reader blinding, in which the treating team could adjust diuretics with knowledge of allocation. Domain 2 and Domain 4 are both High risk for these load-dependent outcomes.
Neurohormonal biomarker (NT-proBNP)	Some concerns	Some concerns	Low	Low	Some concerns	Some concerns	NT-proBNP is laboratory-measured.

EXCEED — Akasaka 2022 (PMID 35212104)

Domain	Signalling-question responses
D1 Bias arising from the randomisation process	1.1 Yes - web-based randomisation with stratification by age, NT-proBNP and glycated haemoglobin. 1.2 Yes - central electronic randomisation. 1.3 Probably no - the report states that groups did not differ except for height.
D2 Bias due to deviations from intended interventions	2.1 YES - open-label with conventional-treatment comparator. 2.2 YES. 2.3 Probably no evidence of differential management is presented. 2.6 Yes - multivariable-adjusted between-group comparison.
D3 Bias due to missing outcome data	3.1 Probably no - 68 of 73 randomised participants were analysed (93%); 40 were randomised to ipragliflozin and 36 analysed, 33 randomised to control and 32 analysed. 3.2 Probably yes. 3.3 Probably no.
D4 Bias in measurement of the outcome	4.1 No - echocardiography, but performed AT EACH PARTICIPATING INSTITUTION rather than in a core laboratory, which the authors themselves identify as a limitation and a source of measurement variability. 4.2 Probably no. 4.3 NOT REPORTED. 4.4 Yes for E/e-prime. 4.5 Possible.
D5 Bias in selection of the reported result	5.1 Yes - co-primary endpoints (E/e-prime and e-prime) were registered (jRCTs051180040). 5.2 No for the co-primary endpoints. 5.3 YES for the results the review emphasises: the LVMi finding in participants aged 70 years or over (n=38) and the NT-proBNP finding in participants with baseline NT-proBNP of 400 pg/mL or more (n=16) are subgroup analyses in a trial whose co-primary endpoints were both neutral and which was underpowered against its own target of 50 per arm.

Outcome stratum	D1	D2	D3	D4	D5	Overall	Justification
Volumetric and structural imaging outcomes (LVEDV/i, LVESV/i, LVEF, LV mass / LVMi)	Low	Some concerns	Some concerns	Some concerns	High	High	Both co-primary endpoints were neutral and every secondary echocardiographic outcome was non-significant. The only positive results are two small subgroup analyses (n=38 and n=16) in an underpowered trial, with site-based rather than core-laboratory echocardiography. Domain 5 is High risk for those subgroup results.
Functional and loading outcomes (GLS, E/e-prime, LAVi, PCWP, atrial strain)	Low	Some concerns	Some concerns	Some concerns	Low	Some concerns	The co-primary E/e-prime and e-prime results were prespecified, neutral and reported with confidence intervals; Domain 5 is Low for these. Site-based echocardiography and unstated reader blinding remain concerns.
Neurohormonal biomarker (NT-proBNP)	Low	Some concerns	Some concerns	Low	High	High	Overall NT-proBNP was non-significant; only the baseline-stratified subgroup (n=16) was significant.

Table S9. Justification for the Absence of Meta-Analysis- outcome specific reasoning

**LVEDV / LVEDVi and LVESV / LVESVi**	Quantitative synthesis was considered separately for CMR and for echocardiography, and within each modality for reduced ejection fraction. It remained inappropriate in both cases. Two trials report absolute volumes and two report volumes indexed to body surface area, which are different quantities on different scales and cannot be combined without an assumption about body surface area that the publications do not support. Point estimates within the CMR set differ by a factor of three and REFORM lies in the opposite direction, so a random-effects estimate would carry a between-study variance large enough to make the summary uninterpretable. Two of the four CMR trials publish no adjusted estimate or confidence interval at all, so their variance cannot be recovered without reconstructing it from arm-level change scores under an independence assumption that the paired design violates.
**LVEF**	A phenotype-specific and modality-specific random-effects meta-analysis was considered and rejected for a different reason: seven of nine estimates are null and mutually consistent, while EMPA-TROPISM lies far outside the confidence intervals of every other trial. Pooling would therefore average one strongly discrepant result with seven concordant nulls, producing a small significant summary effect driven entirely by the outlier. The scientifically informative statement is that seven independent trials exclude an average improvement of more than about five percentage points while one trial reports six, and that this inconsistency is unexplained.
**GLS**	Meta-analysis was considered separately for CMR feature tracking and for speckle-tracking echocardiography, because the two techniques measure different quantities with different normative ranges. Within the speckle-tracking set it remained inappropriate because effect size correlates almost perfectly with risk of bias: the only estimate from a blinded core laboratory is the smallest, and two of the four trials publish no adjusted between-group estimate. A pooled estimate would be dominated by the trials least able to exclude measurement bias. Sign conventions also differ between publications, so pooling requires manual reorientation of each estimate.
**LV mass / LVMi**	As for volumes, absolute and indexed mass are reported in different trials and are not interchangeable. Within the CMR set two trials report reductions and two report none, with point estimates differing several fold, and the larger of the two positive estimates is an exploratory outcome analysed on a per-protocol set from a trial whose primary endpoint was neutral.
**LAVI**	Estimates span an order of magnitude, from -1.0 to -13.1 mL/m2, and the extreme value arises from a trial in which the control arm deteriorated markedly, so the heterogeneity is attributable to a specific identifiable design problem rather than to chance. One contributing estimate carries a published confidence interval incompatible with its published p value, and one trial reports non-indexed atrial volume, which cannot enter an indexed synthesis. In EFFORT atrial volume is additionally determined by the regurgitant lesion defining the population.

**E/e'**	Only two trials contributed in each phenotype. In reduced ejection fraction neither published an adjusted between-group estimate, so there is nothing to pool. In preserved ejection fraction the two confidence intervals do not overlap at all: the trial in which the outcome was co-primary excludes the effect that the open-label trial reports. Pooling two mutually exclusive estimates would generate a spurious intermediate value and conceal precisely the conflict that matters.
**PCWP**	Only two trials measured filling pressure invasively, one in each phenotype, so no within-phenotype pooling is possible. Pooling across phenotypes was rejected because resting and exercise physiology differ fundamentally between reduced and preserved ejection fraction, and because one of the two trials did not meet its prespecified haemodynamic primary endpoint while the other did.
**NT-proBNP**	Meta-analysis was considered separately by phenotype and rejected in both. Results are reported variously as absolute change, percentage change and log-transformed ratios, which are not interchangeable without access to the underlying distributions. The direction of effect is inconsistent, and critically the two trials at low risk of bias for this outcome disagree with each other, while the two trials in which NT-proBNP was the registered primary endpoint were both neutral. Several of the significant results are post hoc subgroups defined by baseline concentration, which cannot be pooled with overall estimates.

Table S10. Study-publication map

Distinguishing unique trials from publications, and identifying overlapping populations and phenotype-specific cohorts.

Unique clinical trial	Registration	Associated publication(s)	Overlapping populations	Phenotype-specific cohorts and EF range	Outcomes extracted from each publication	Synthesis in which each publication contributed
EMPA-TROPISM (ATRU-4)	NCT03485222	Santos-Gallego 2021, J Am Coll Cardiol 77:243-255 (PMID 33197559)	None	Single cohort, LVEF < 50% (mixed reduced and mildly reduced)	LVEDV, LVESV, LVEF, LV mass, NT-proBNP	Reduced-EF volumetric, LVEF, LV mass and NT-proBNP syntheses
SUGAR-DM-HF	NCT03485092	Lee 2021, Circulation 143:516-525 (PMID 33186500)	None	Single cohort, LVEF ≤ 40%	LVESVi, LVEDVi, LVEF, LVMi, LAVi, GLS (CMR feature tracking), NT-proBNP	Reduced-EF CMR volumetric, LVEF, LV mass, LAVi, CMR-GLS and NT-proBNP syntheses
Empire HF	NCT03198585	TWO PUBLICATIONS: (1) Omar 2021, JAMA Cardiol 6:836-840 (PMID 33404637) — echocardiographic substudy; (2) Omar 2020, J Am Coll Cardiol 76:2740-2751 (PMID 33272368) — haemodynamic substudy	YES — OVERLAPPING POPULATIONS. The echocardiographic substudy reports the full randomised cohort (n = 190); the haemodynamic substudy reports a single-centre subset (n = 70) drawn from the same trial. Participants are counted ONCE (n = 190) in the participant total.	Single cohort, LVEF ≤ 40%	Echocardiographic substudy: LVESVi, LVEDVi, LAVi, LVEF, LVMi, GLS. Haemodynamic substudy: PCWP at rest and exercise, cardiac index, plasma volume, EDPVR	Reduced-EF echocardiographic volumetric, LVEF, LV mass, LAVi, GLS syntheses (echo substudy) and the reduced-EF PCWP synthesis (haemodynamic substudy). No outcome is double-counted.
REFORM	NCT02397421	Singh 2020, Diabetes Care 43:1356-1359 (PMID 32245746)	None	Single cohort, documented reduced LVEF (no numeric threshold stated)	LVESV/i, LVEDV/i, LVEF, LVMi, indexed LA volume	Reduced-EF CMR volumetric, LVEF, LV mass and LAVi syntheses
EMPA-VISION	NCT03332212	ONE PUBLICATION reporting TWO SEPARATELY ANALYSED COHORTS: Hundertmark 2023, Circulation	NO overlap between the two cohorts — they are distinct participants (36 HFrEF and 36	HFrEF cohort LVEF ≤ 40% (n = 36 randomised, 35 analysed); HFpEF cohort	Both cohorts: PCr/ATP (primary, neutral), LVEDV, LVEF, LV mass, LVMi, multidirectional strain. LVESV	HFrEF cohort contributes to the reduced-EF CMR syntheses;

		147:1654-1669 (PMID 37070436)	HFpEF) randomised within one trial and analysed separately, so they contribute to different phenotype syntheses.	LVEF ≥ 50% (n = 36 randomised, 24 analysed)	was NOT reported.	HFpEF cohort contributes to the preserved-EF syntheses. Counted as ONE trial but TWO cohorts.
DAPA-ECHO	NCT05179668	Pastore 2024, JACC Cardiovasc Imaging 17:1399-1408 (PMID 39023499)	None	MIXED: LVEF < 50%, of whom 62% HFrEF (n = 55) and 38% HFmrEF (n = 33). Subgroup analyses by phenotype were reported.	LV GLS, LA reservoir and contractile strain, RV free-wall strain, E/e-prime, LVEDVi, LVESVi, LVEF, LAVi, NT-proBNP	Reduced-EF speckle-tracking GLS, E/e-prime, volumetric, LVEF and LAVi syntheses
Reis 2022	Not registered	Reis 2022, J Clin Med 11:2935 (PMID 35629062)	None	Single cohort, LVEF < 50% (mixed reduced and mildly reduced)	Peak VO2 (primary), GLS, E/e-prime, indexed LA volume, LVEF, LV end-diastolic and end-systolic DIAMETERS (volumes not reported), NT-proBNP	Reduced-EF GLS, E/e-prime, LAVi, LVEF and NT-proBNP syntheses
Fu 2023	ChiCTR2300072707	Fu 2023, BMC Cardiovasc Disord 23:544 (PMID 37940879)	None	Single cohort, LVEF ≤ 40%	LVEF (primary), LVEDV, LVESV, LV end-diastolic diameter, VTI	Reduced-EF echocardiographic volumetric and LVEF syntheses
EFFORT	NCT04739215	Kang 2024, Circulation 149:1865-1874 (PMID 38690659)	None	MIXED: LVEF 35% to < 50%, spanning HFrEF (35-40%) and HFmrEF (41-49%). NOT a pure HFmrEF cohort. All participants additionally had substantial functional mitral regurgitation.	EROA (primary), regurgitant volume, GLS, LAVi, LV volume indices, LVEF, NT-proBNP	Reduced-EF speckle-tracking GLS, LAVi, volumetric, LVEF and NT-proBNP syntheses
CANDLE	UMIN000017669	Tanaka 2020, ESC Heart Fail 7:1585-1594 (PMID 32349193)	None	MIXED EJECTION FRACTION: any LVEF, mean 57.6 ± 14.6%, 71% with LVEF ≥ 50%. Randomisation was stratified at LVEF 40%, whereas the reported subgroups are split at 50%. Assigned to neither phenotype exclusively.	NT-proBNP ratio of percentage change (primary, non-inferiority NOT met), post hoc NT-proBNP subgroups by baseline concentration and by LVEF stratum, echocardiographic LVEF and E/e-prime (supplementary table, not available to the review team)	Preserved-EF NT-proBNP synthesis, through its LVEF ≥ 50% post hoc subgroup only. Contributes no verified imaging outcome.
CAMEO-DAPA	NCT04730947	Borlaug 2023, Circulation 148:834-844 (PMID 37534453)	None	Single cohort, LVEF ≥ 50% with invasively confirmed exercise-induced elevation of PCWP	PCWP at rest and peak exercise (composite primary, MET), right atrial and mean pulmonary artery pressure, plasma volume, body weight, NT-proBNP (exploratory)	Preserved-EF PCWP and NT-proBNP syntheses
Ovchinnikov 2025	Reported in source	Ovchinnikov 2025, Cardiovasc Diabetol 24:196 (PMID 40346546)	None	Single cohort, LVEF ≥ 50%, all with type 2 diabetes	6-minute walk distance (primary), E/e-prime at rest and exercise, LAVi, LV GLS, LVMi, LVEDV, LVEF, e-prime, LA reservoir strain, cardiac reserves, NT-proBNP	Preserved-EF E/e-prime, LAVi, GLS, LV mass, volumetric, LVEF and NT-proBNP syntheses
EXCEED	jRCTs051180040	Akasaka 2022, Geriatr Gerontol Int 22:298-304 (PMID 35212104)	None	Single cohort, LVEF ≥ 50%, all with type 2 diabetes; atrial fibrillation and valvular disease excluded	E/e-prime and e-prime (co-primary, both NEUTRAL), LVEF, LVEDV, LVESV, absolute LA volume (not indexed), LVMi, NT-proBNP, plus subgroup analyses in participants aged ≥ 70 years and with baseline NT-proBNP ≥ 400 pg/mL	Preserved-EF E/e-prime, volumetric, LVEF, LV mass and NT-proBNP syntheses

Thirteen unique randomised trials are represented by fourteen publications.
